# Supplementary figures and images for: Chromosome length is constrained by spindle scaling to ensure faithful mitosis in mammals (part 1 of 2)
Source: Mol Syst Biol. 2026 Jan 15;22(4):480–96. doi: 10.1038/s44320-026-00188-8 (PMC13047055; doi:10.1038/s44320-026-00188-8)

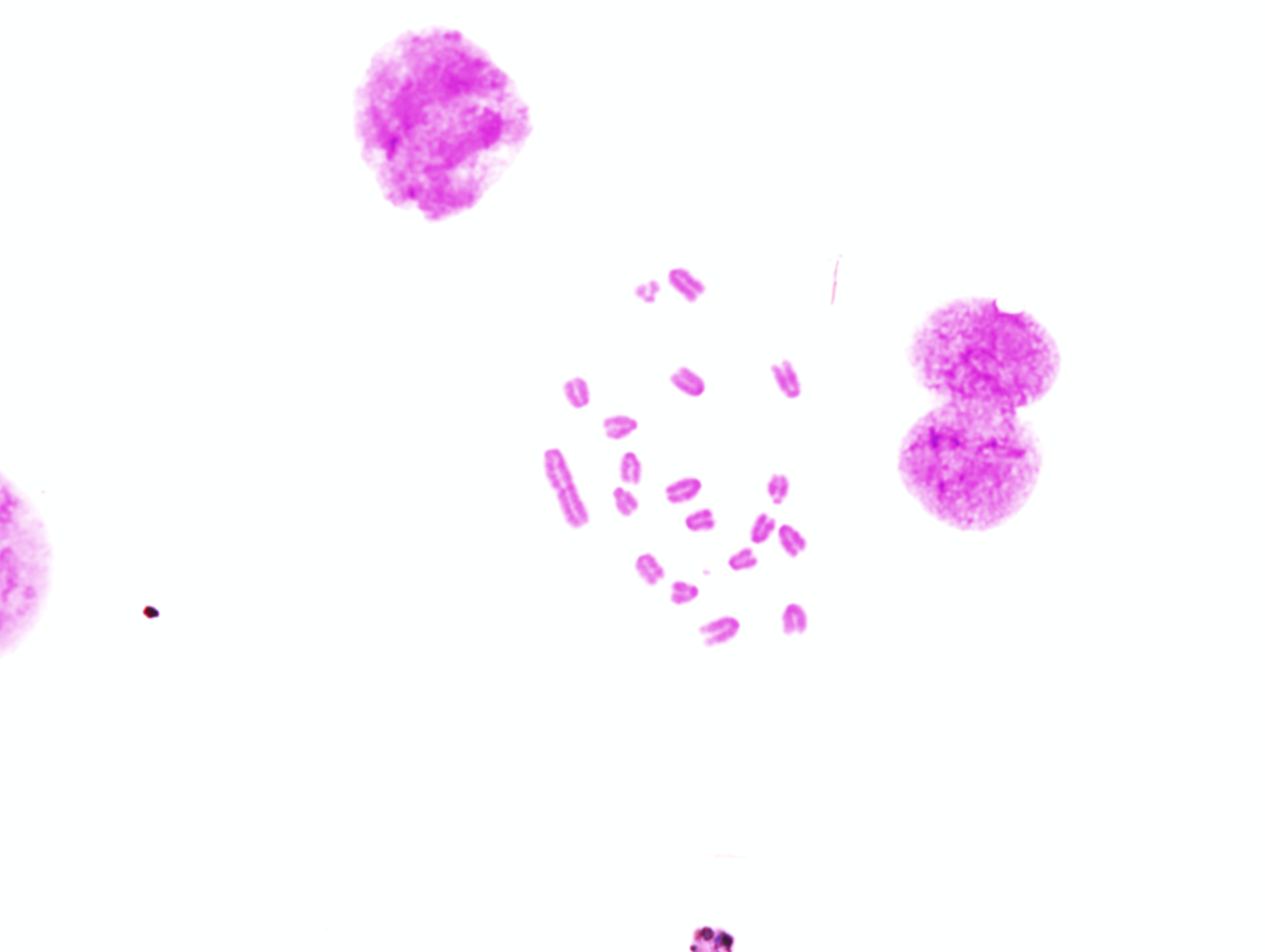

Supplement: Supplementary file 15 — Source data Fig. 1 [file 44320_2026_188_MOESM15_ESM.zip › Figure 1/1A/G-banding Karyotype Chr2+1 haESC.tif]

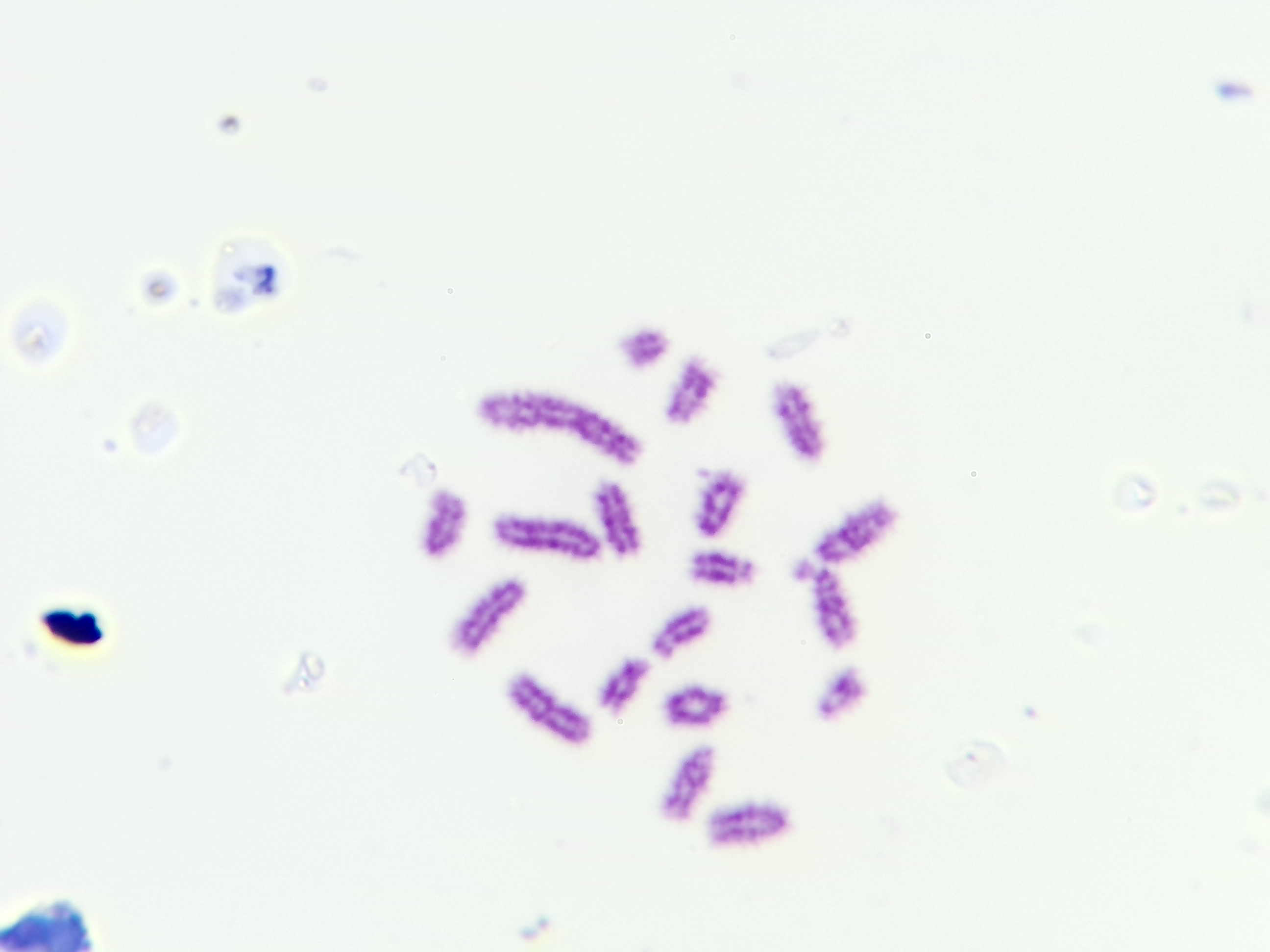

Supplement: Supplementary file 15 — Source data Fig. 1 [file 44320_2026_188_MOESM15_ESM.zip › Figure 1/1A/G-banding Karyotype Chr2+3 haESC.tif]

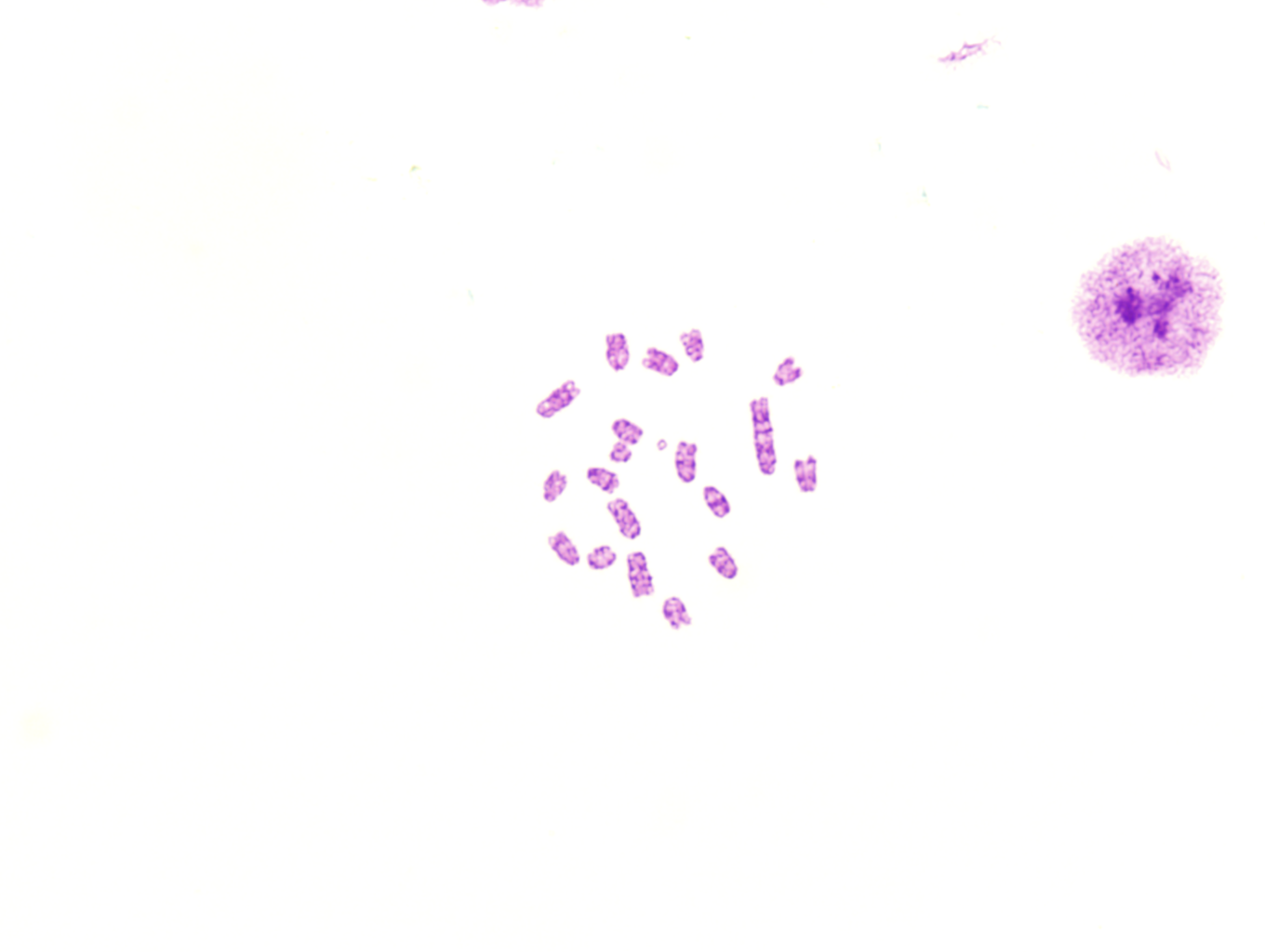

Supplement: Supplementary file 15 — Source data Fig. 1 [file 44320_2026_188_MOESM15_ESM.zip › Figure 1/1A/G-banding Karyotype Chr4+5 haESC.tif]

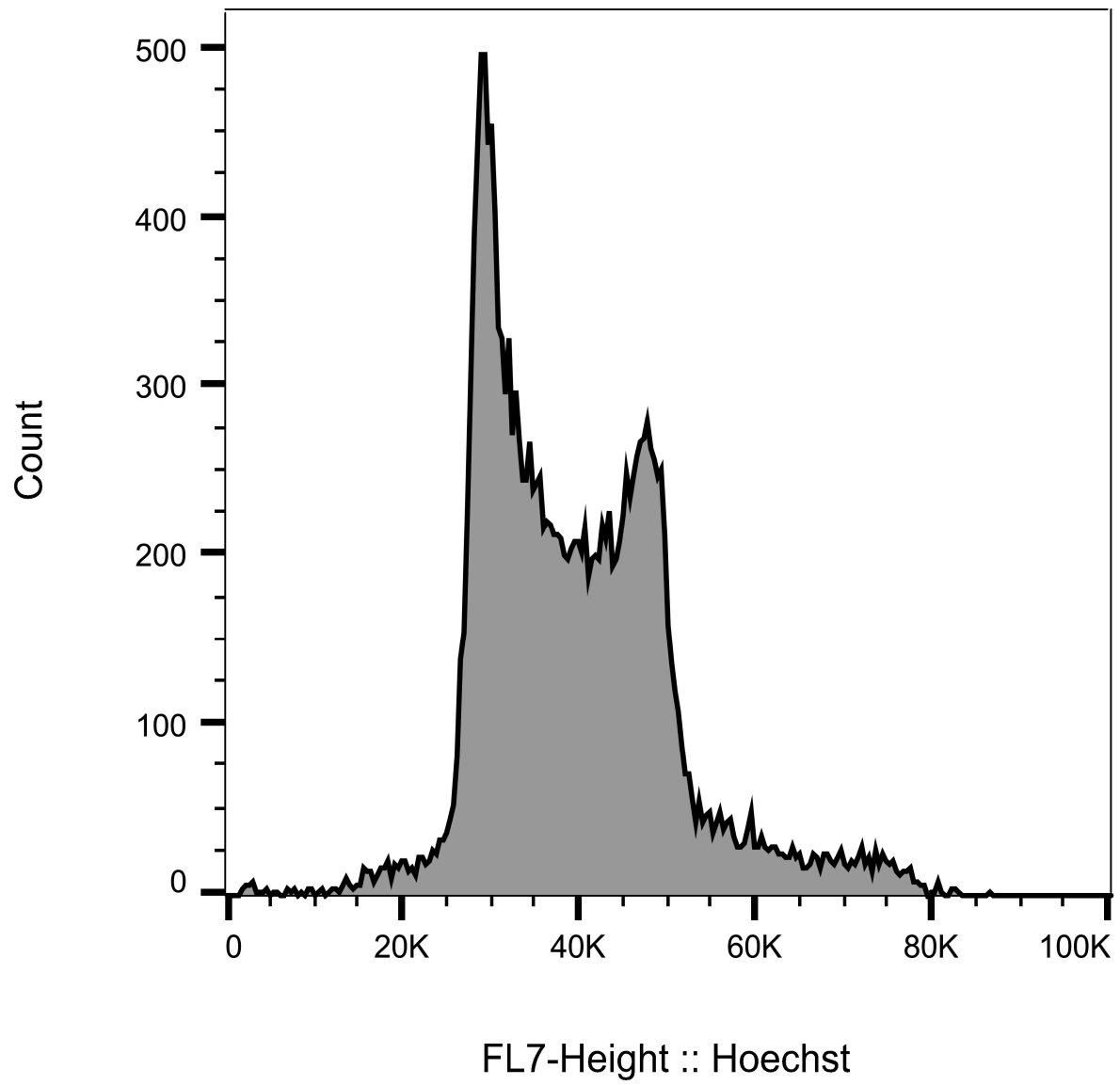

Supplement: Supplementary file 15 — Source data Fig. 1 [file 44320_2026_188_MOESM15_ESM.zip › Figure 1/1B/FACS Chr1+2‘ Day0.pdf]

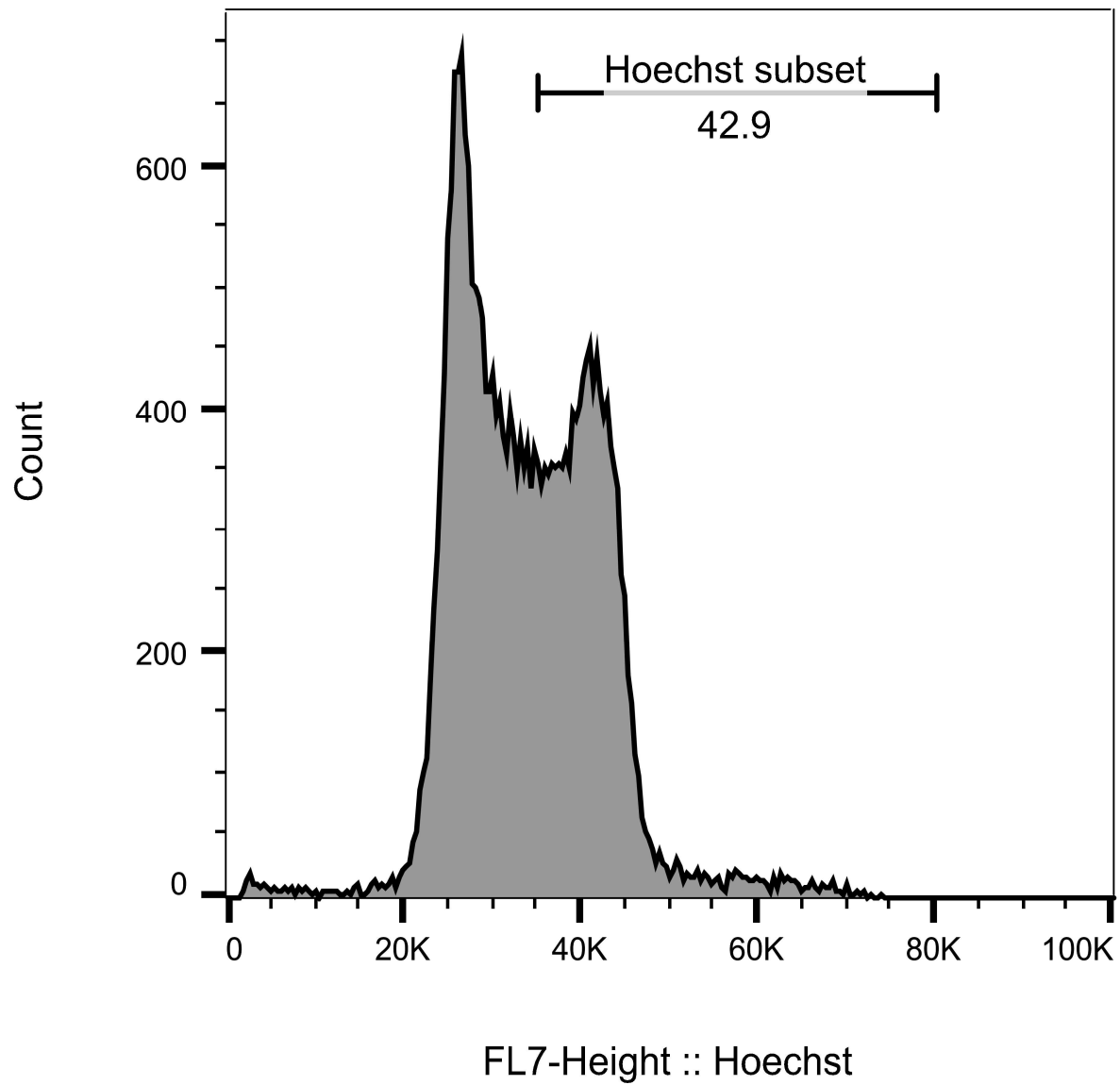

Supplement: Supplementary file 15 — Source data Fig. 1 [file 44320_2026_188_MOESM15_ESM.zip › Figure 1/1B/FACS Chr1+2‘ Day26.pdf]

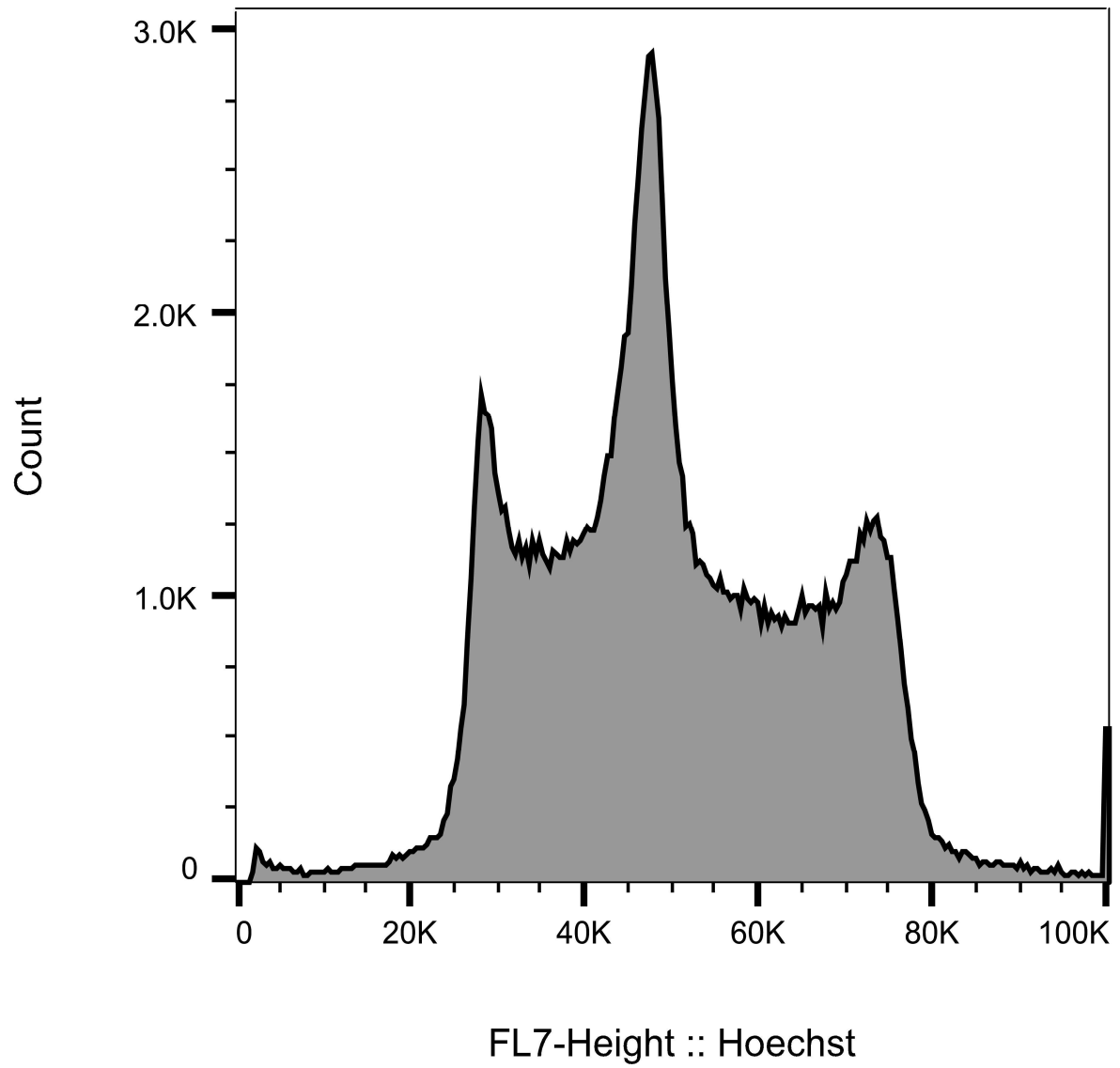

Supplement: Supplementary file 15 — Source data Fig. 1 [file 44320_2026_188_MOESM15_ESM.zip › Figure 1/1B/FACS Chr2+1 Day0.pdf]

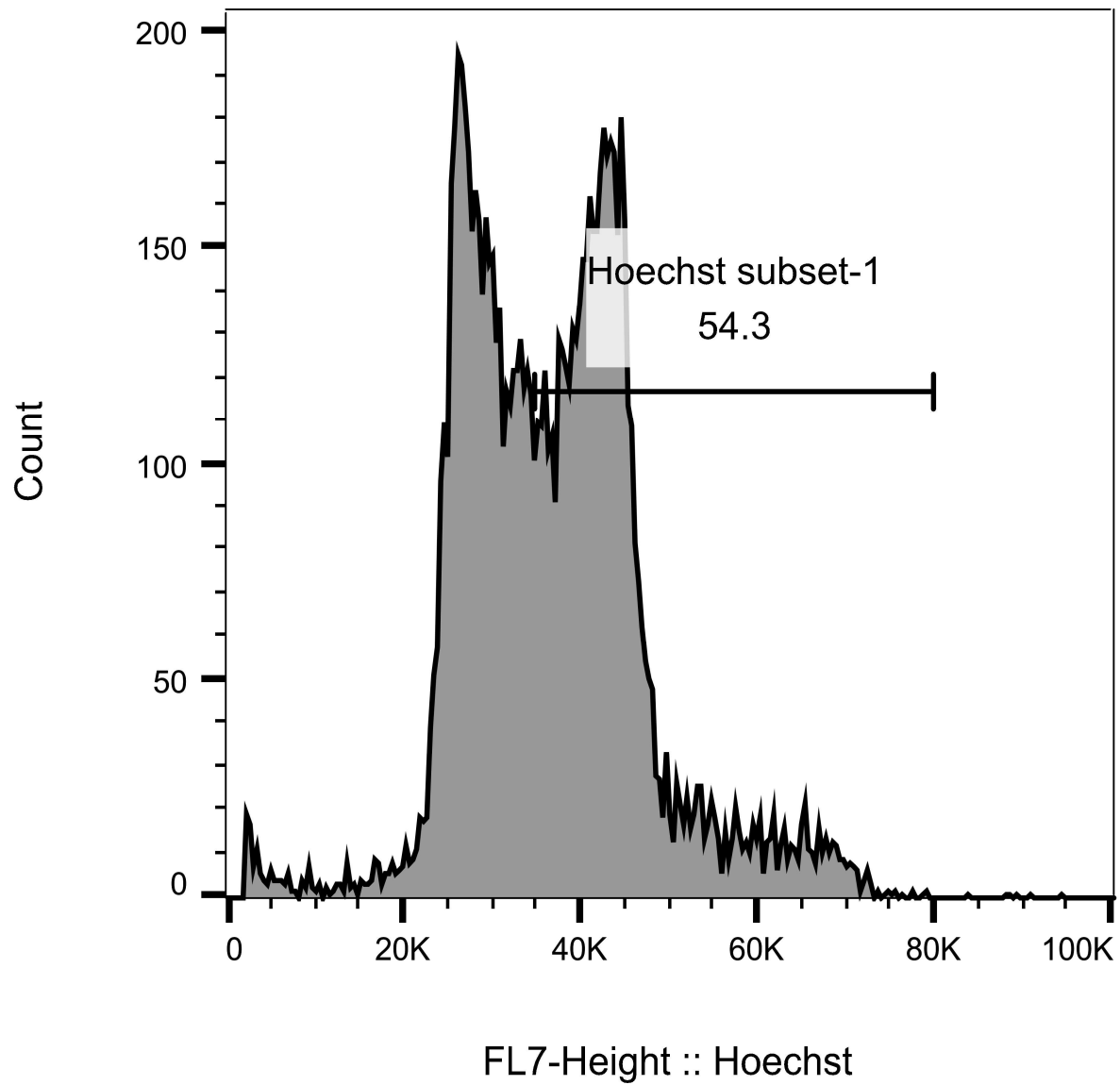

Supplement: Supplementary file 15 — Source data Fig. 1 [file 44320_2026_188_MOESM15_ESM.zip › Figure 1/1B/FACS Chr2+1 Day26.pdf]

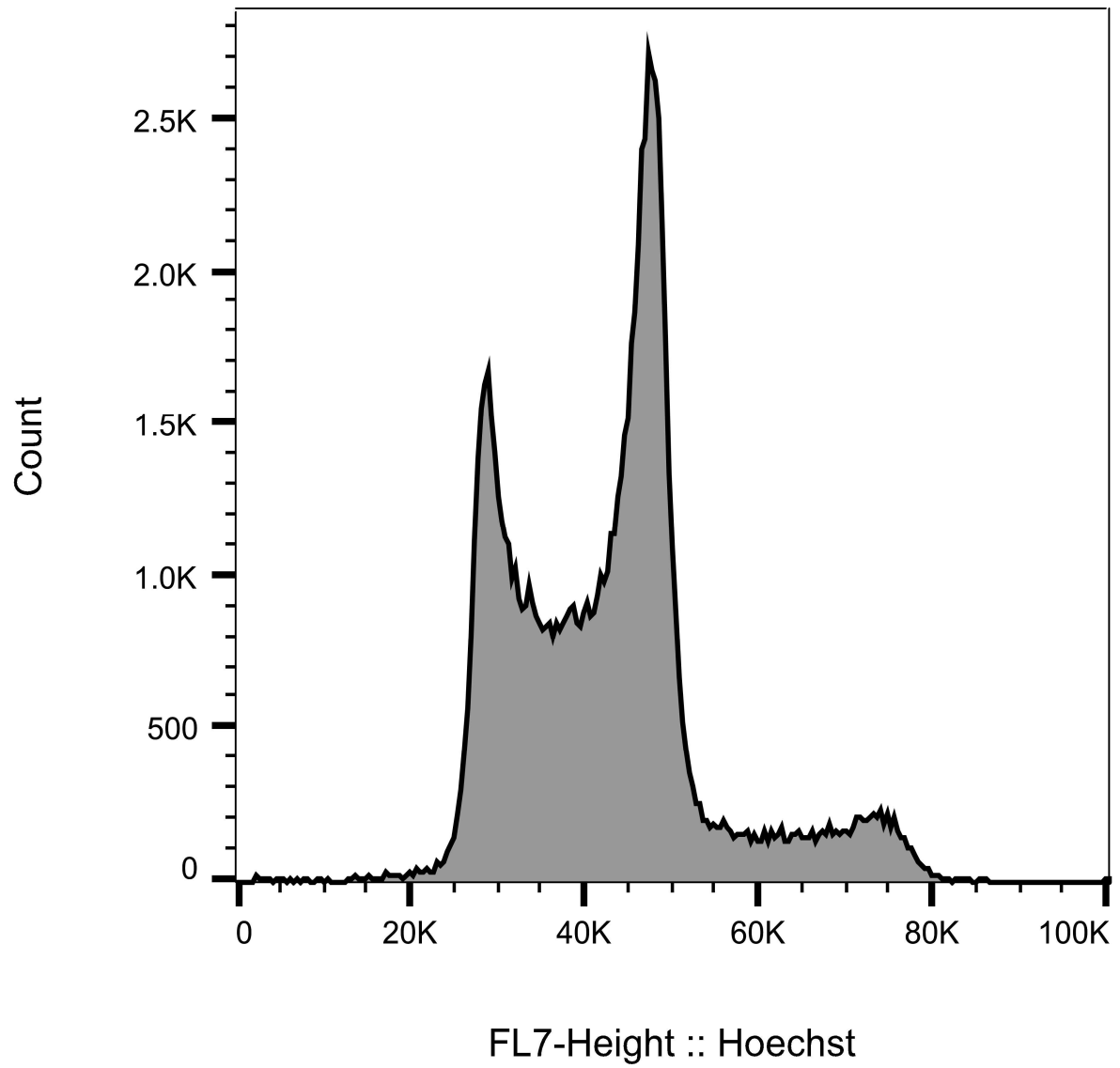

Supplement: Supplementary file 15 — Source data Fig. 1 [file 44320_2026_188_MOESM15_ESM.zip › Figure 1/1B/FACS Chr2+3 Day0.pdf]

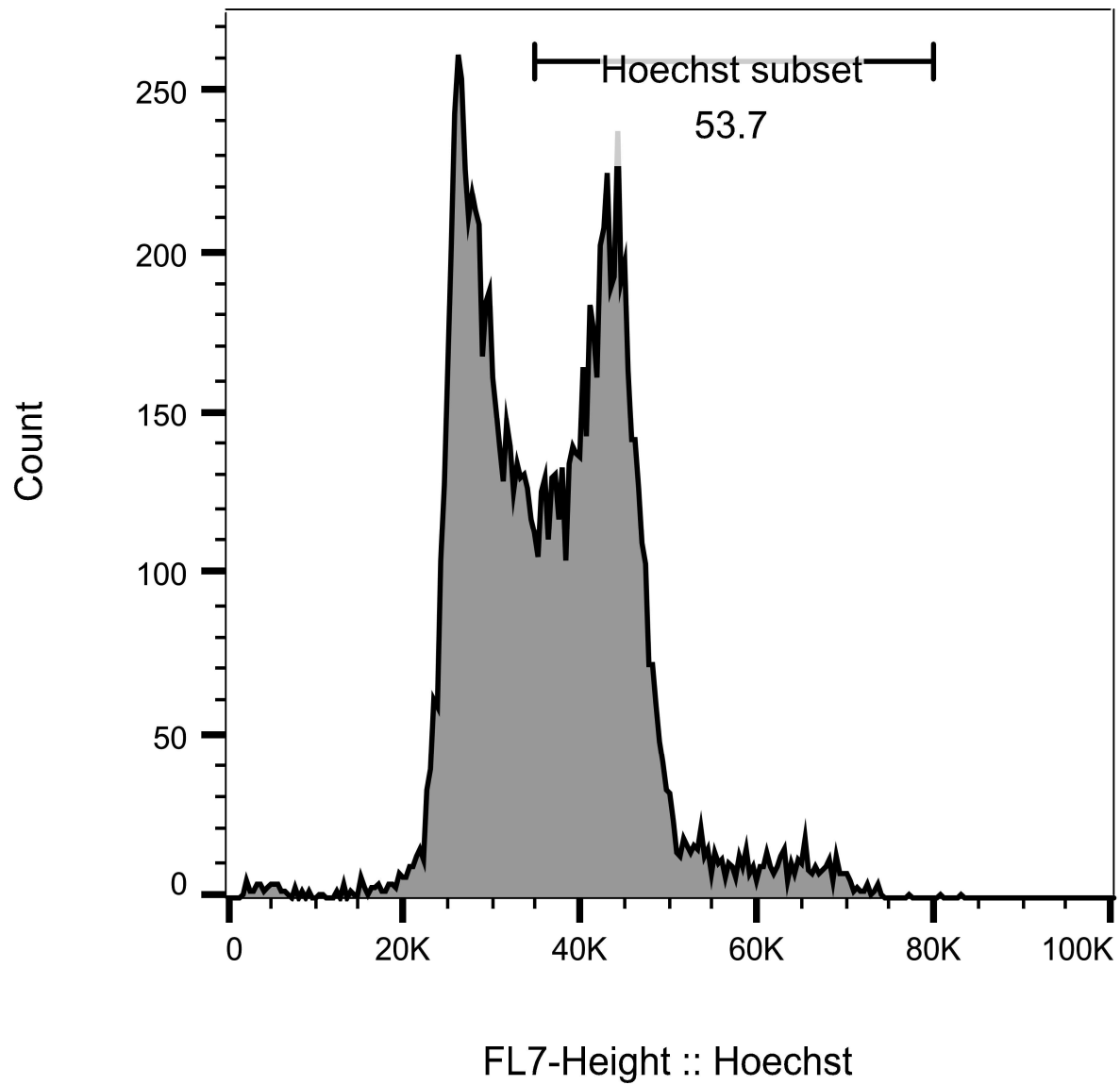

Supplement: Supplementary file 15 — Source data Fig. 1 [file 44320_2026_188_MOESM15_ESM.zip › Figure 1/1B/FACS Chr2+3 Day26.pdf]

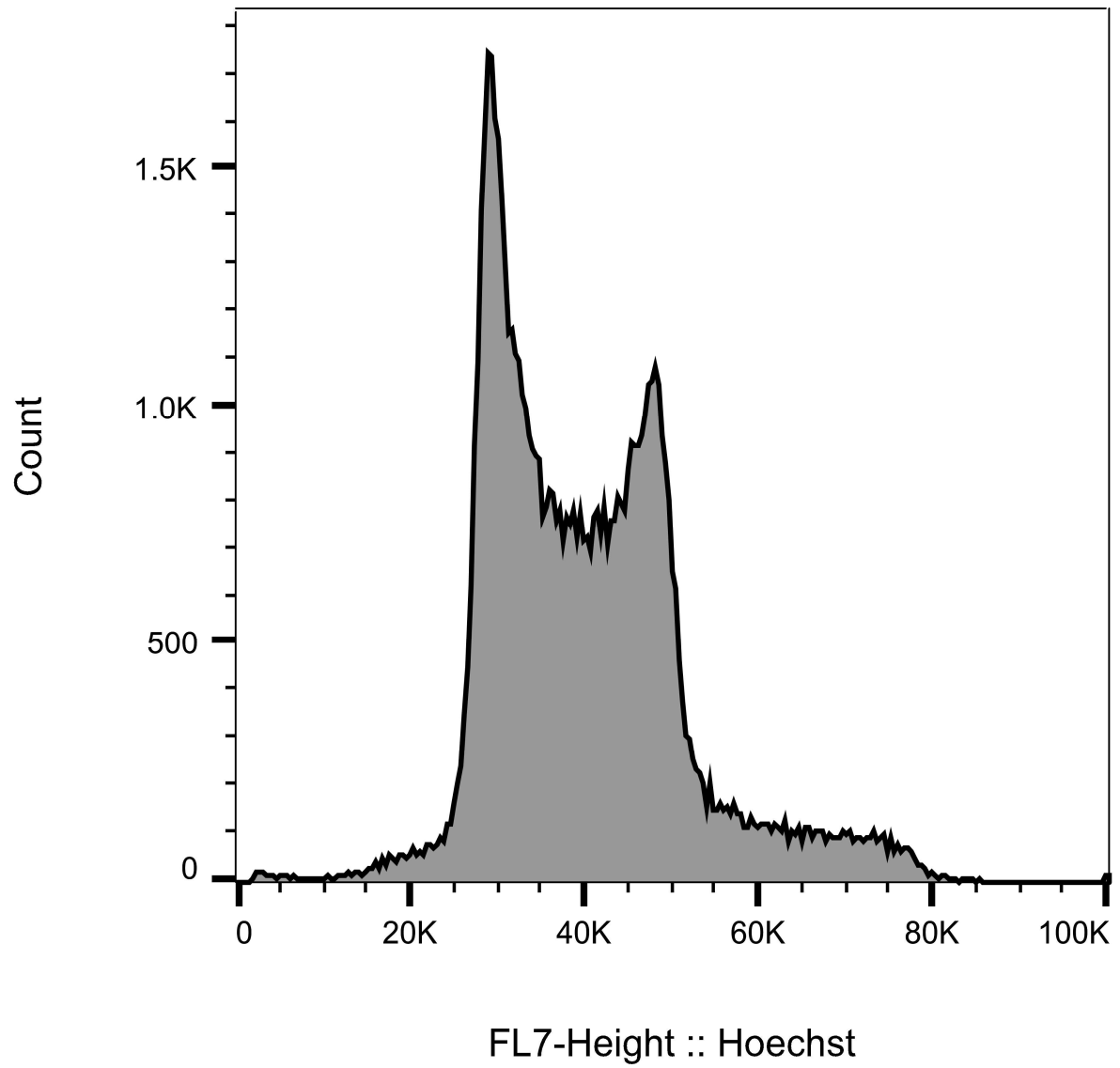

Supplement: Supplementary file 15 — Source data Fig. 1 [file 44320_2026_188_MOESM15_ESM.zip › Figure 1/1B/FACS Chr4+5 Day0.pdf]

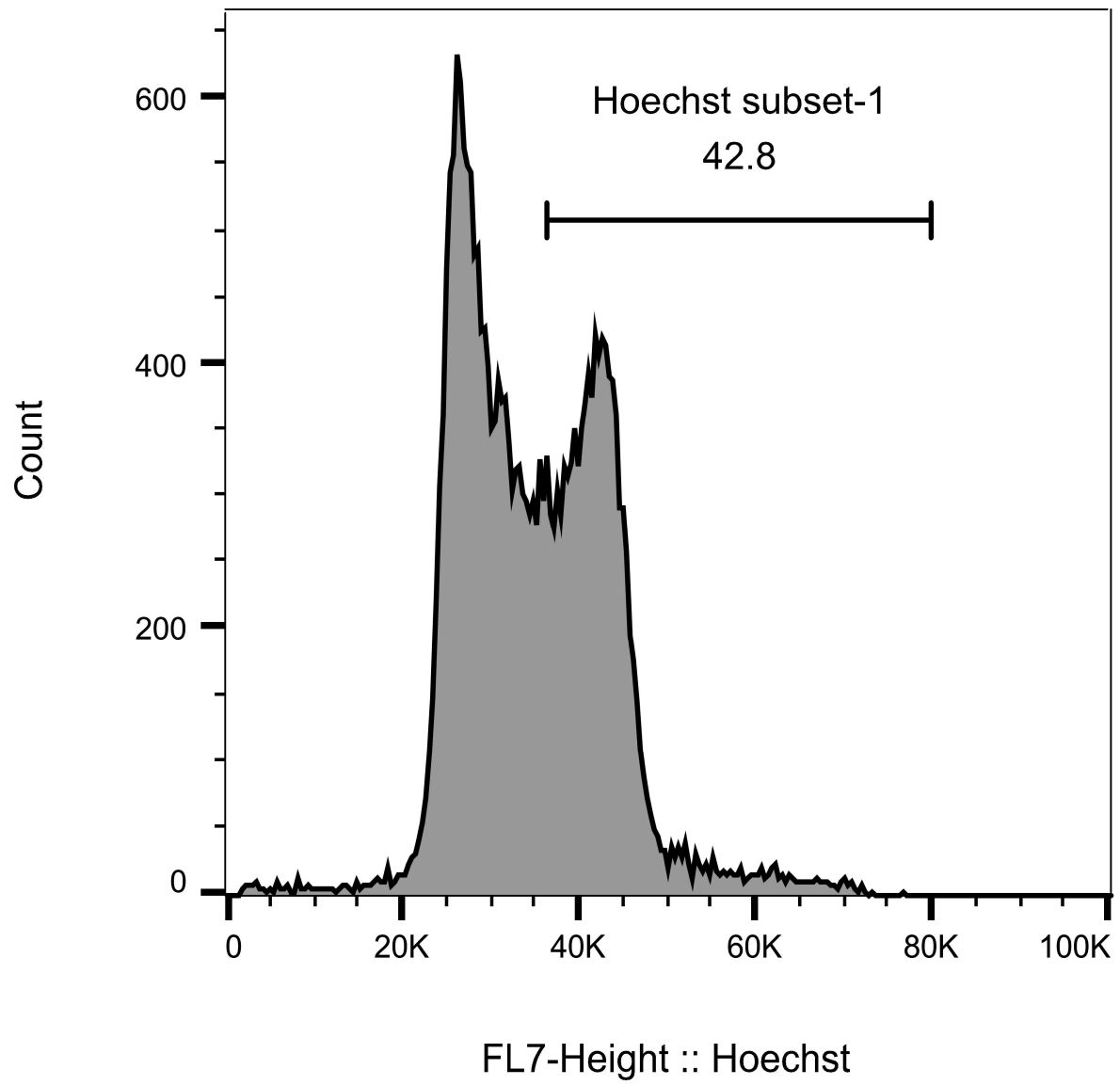

Supplement: Supplementary file 15 — Source data Fig. 1 [file 44320_2026_188_MOESM15_ESM.zip › Figure 1/1B/FACS Chr4+5 Day26.pdf]

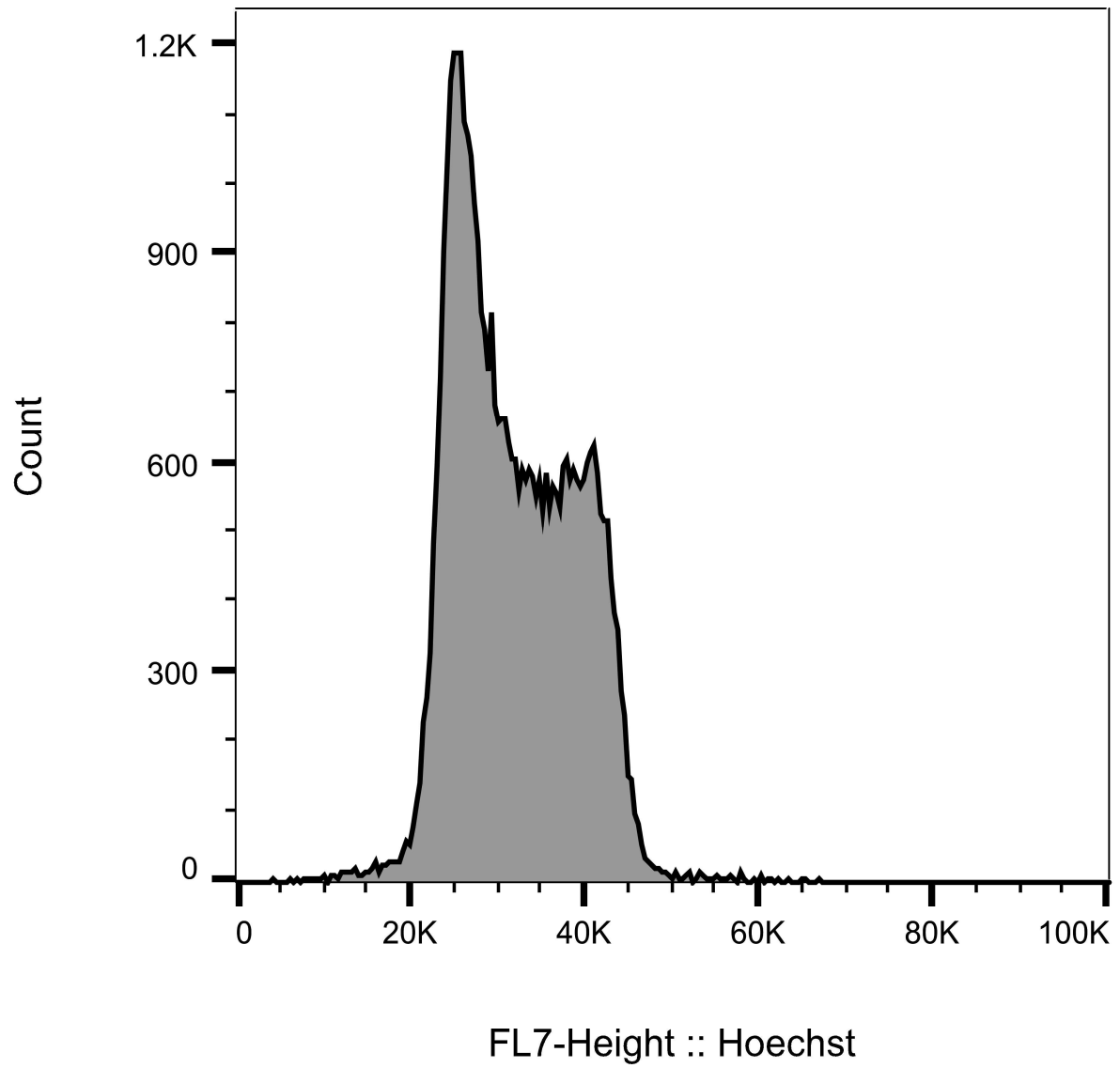

Supplement: Supplementary file 15 — Source data Fig. 1 [file 44320_2026_188_MOESM15_ESM.zip › Figure 1/1B/FACS WT Day0.pdf]

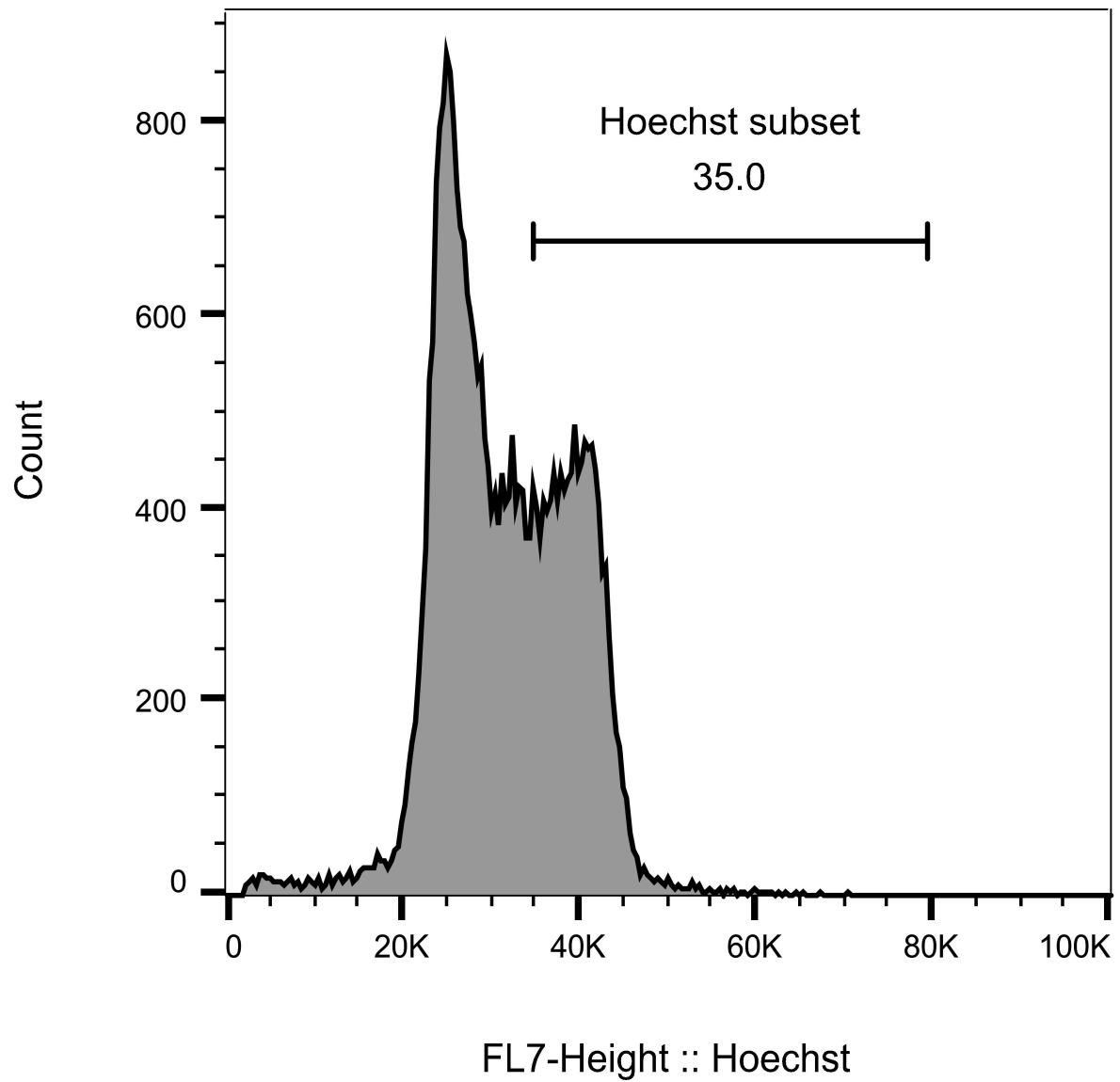

Supplement: Supplementary file 15 — Source data Fig. 1 [file 44320_2026_188_MOESM15_ESM.zip › Figure 1/1B/FACS WT Day26.pdf]

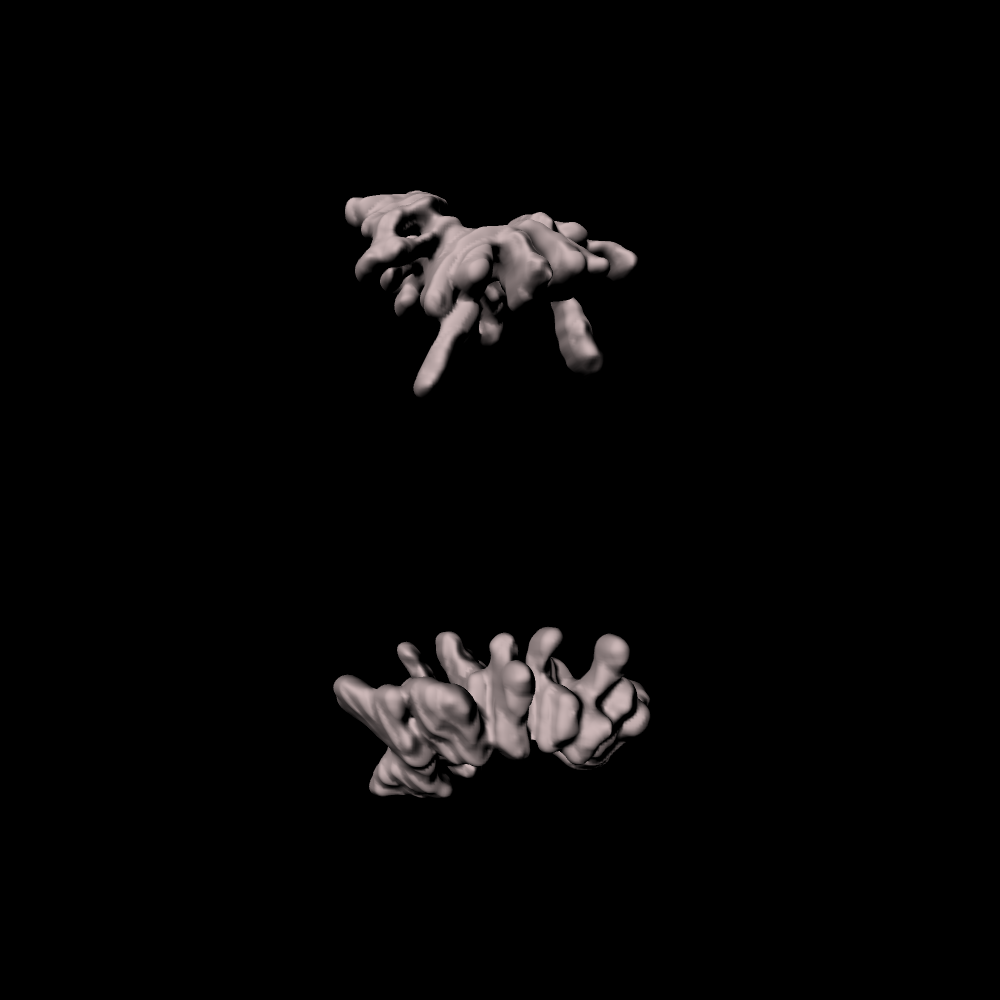

Supplement: Supplementary file 16 — Source data Fig. 2 [file 44320_2026_188_MOESM16_ESM.zip › Figure 2/2A/Live cell imaging 3D Chr1+2'.tif]

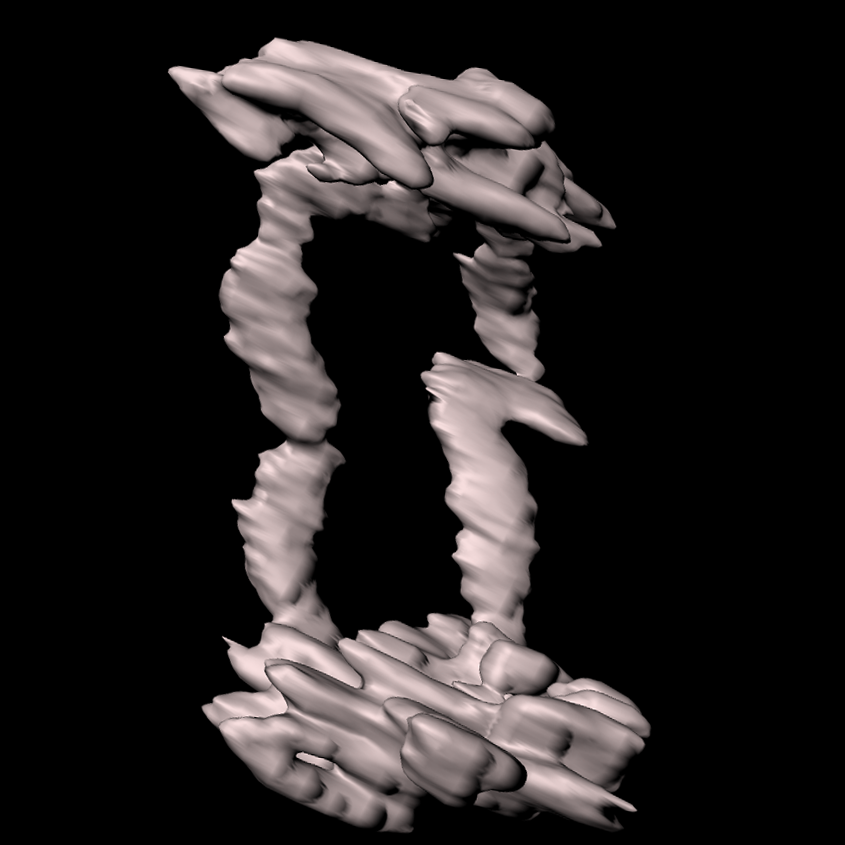

Supplement: Supplementary file 16 — Source data Fig. 2 [file 44320_2026_188_MOESM16_ESM.zip › Figure 2/2A/Live cell imaging 3D Chr2+1.tif]

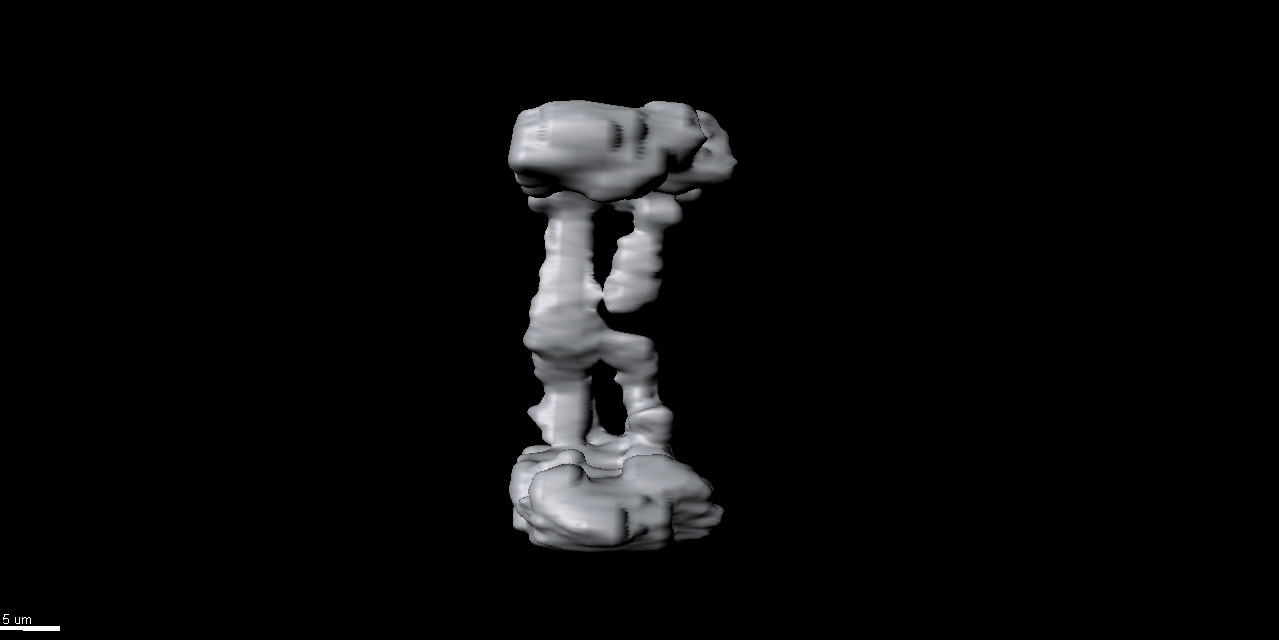

Supplement: Supplementary file 16 — Source data Fig. 2 [file 44320_2026_188_MOESM16_ESM.zip › Figure 2/2A/Live cell imaging 3D Chr2+3.tif]

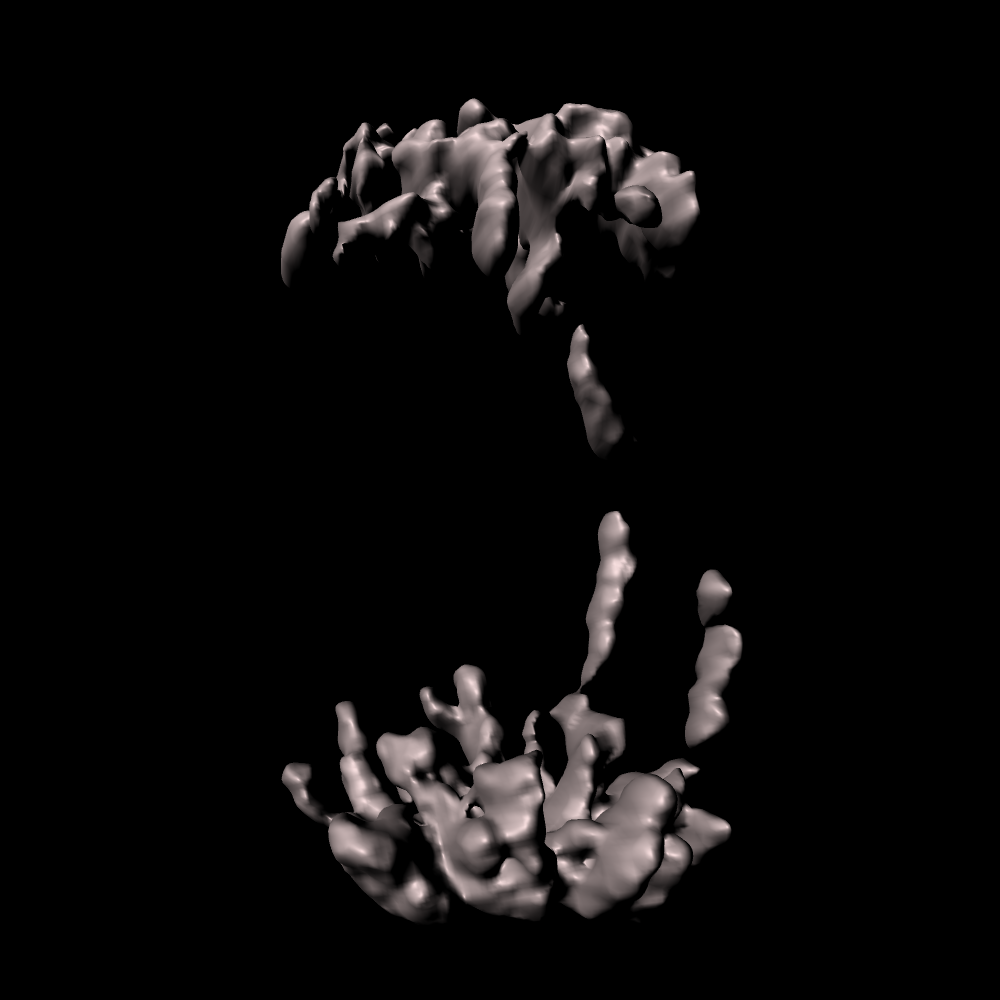

Supplement: Supplementary file 16 — Source data Fig. 2 [file 44320_2026_188_MOESM16_ESM.zip › Figure 2/2A/Live cell imaging 3D Chr4+5.tif]

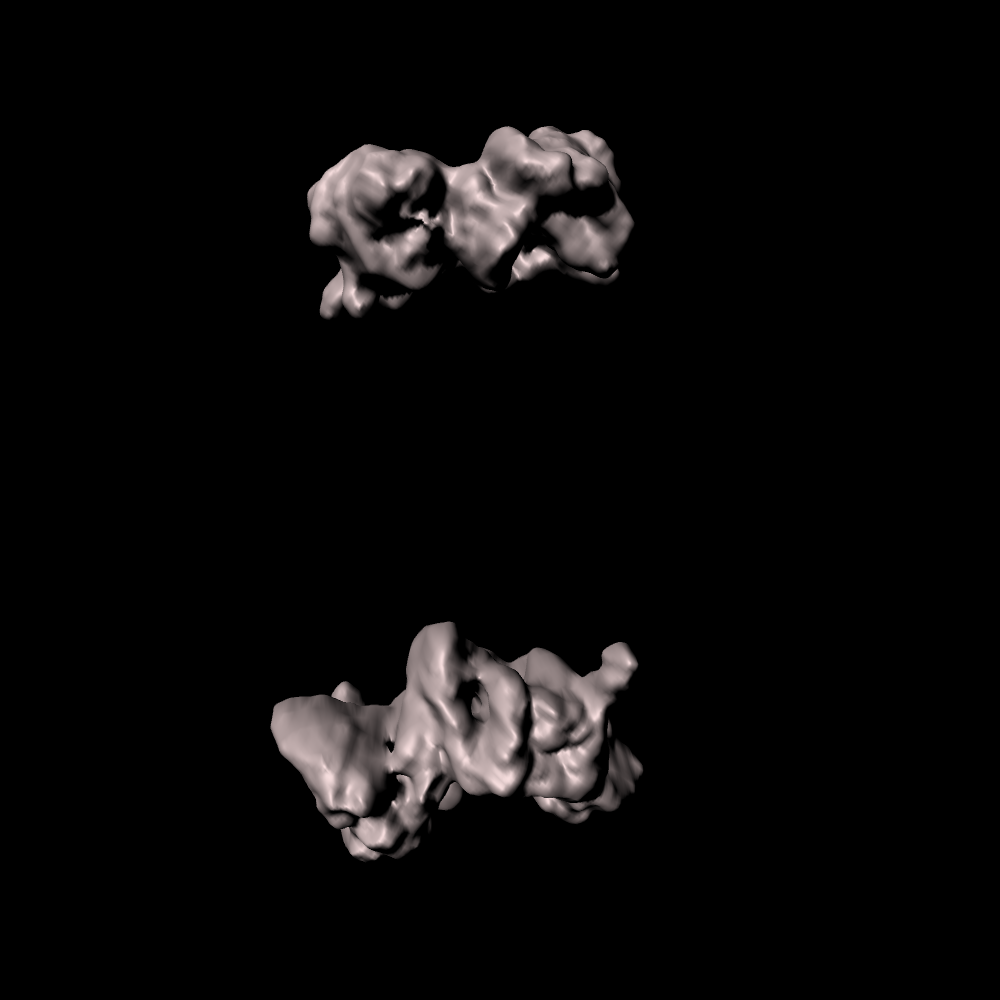

Supplement: Supplementary file 16 — Source data Fig. 2 [file 44320_2026_188_MOESM16_ESM.zip › Figure 2/2A/Live cell imaging 3D WT.tif]

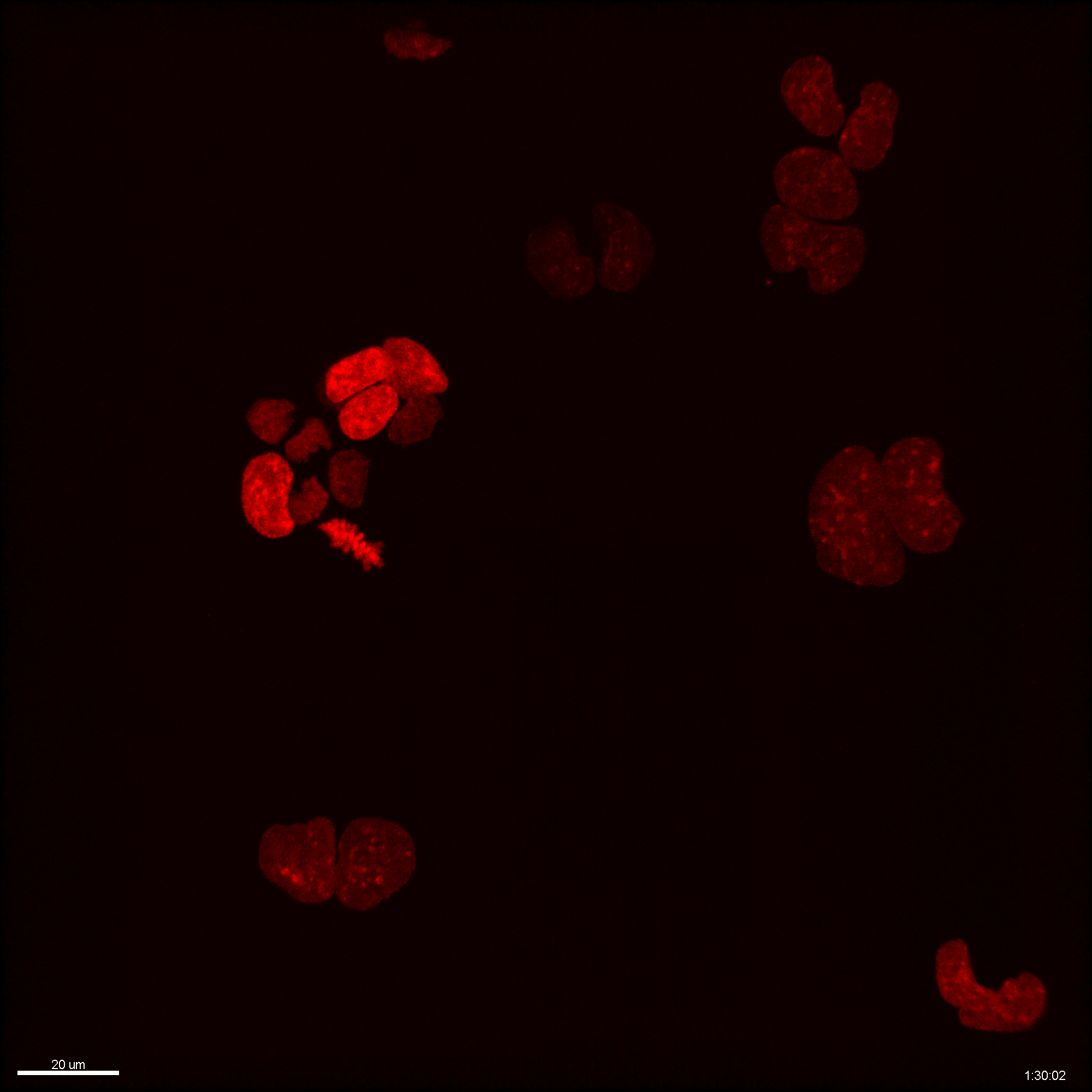

Supplement: Supplementary file 16 — Source data Fig. 2 [file 44320_2026_188_MOESM16_ESM.zip › Figure 2/2B/Live cell imaging Chr1+2' 0 min.tif]

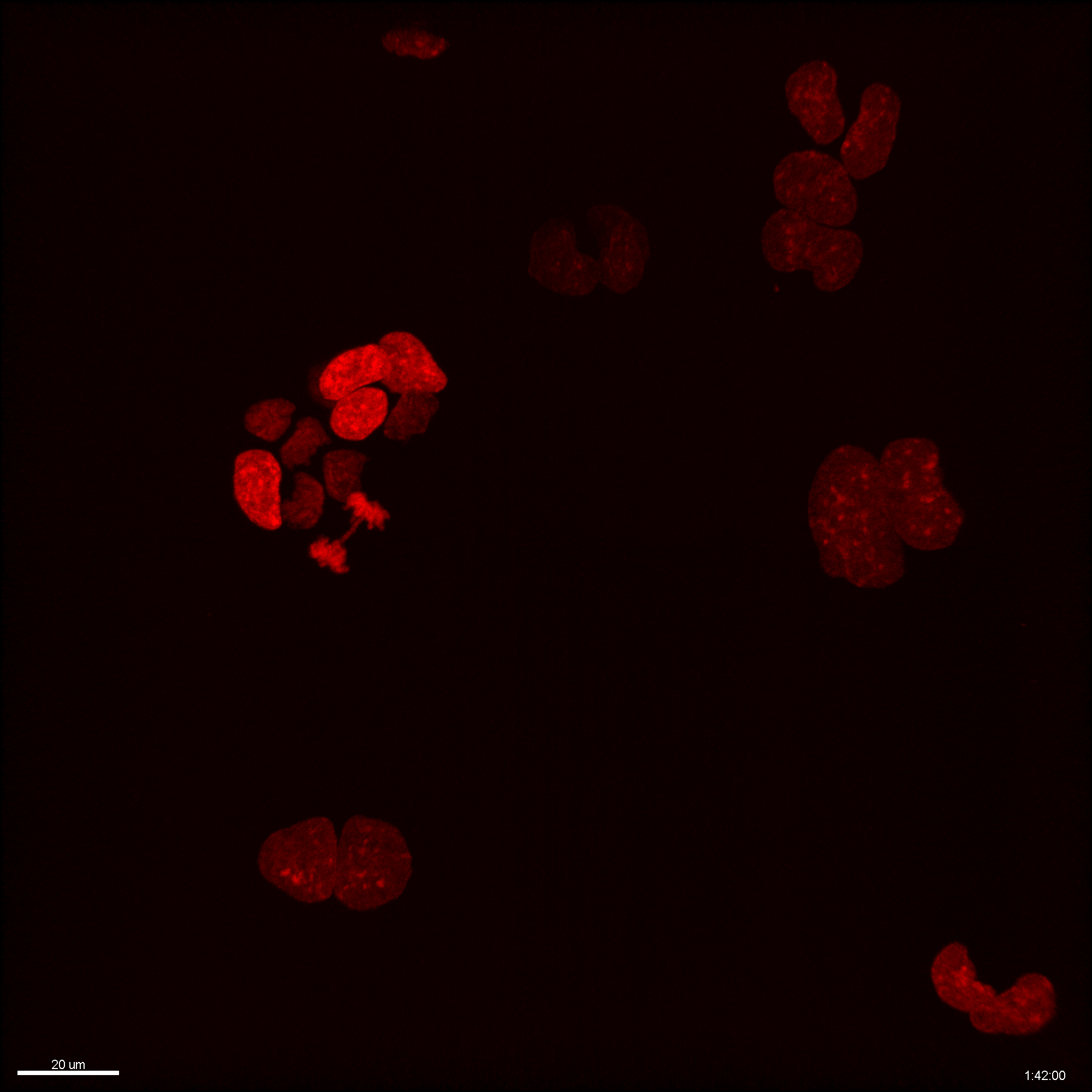

Supplement: Supplementary file 16 — Source data Fig. 2 [file 44320_2026_188_MOESM16_ESM.zip › Figure 2/2B/Live cell imaging Chr1+2' 12 min.tif]

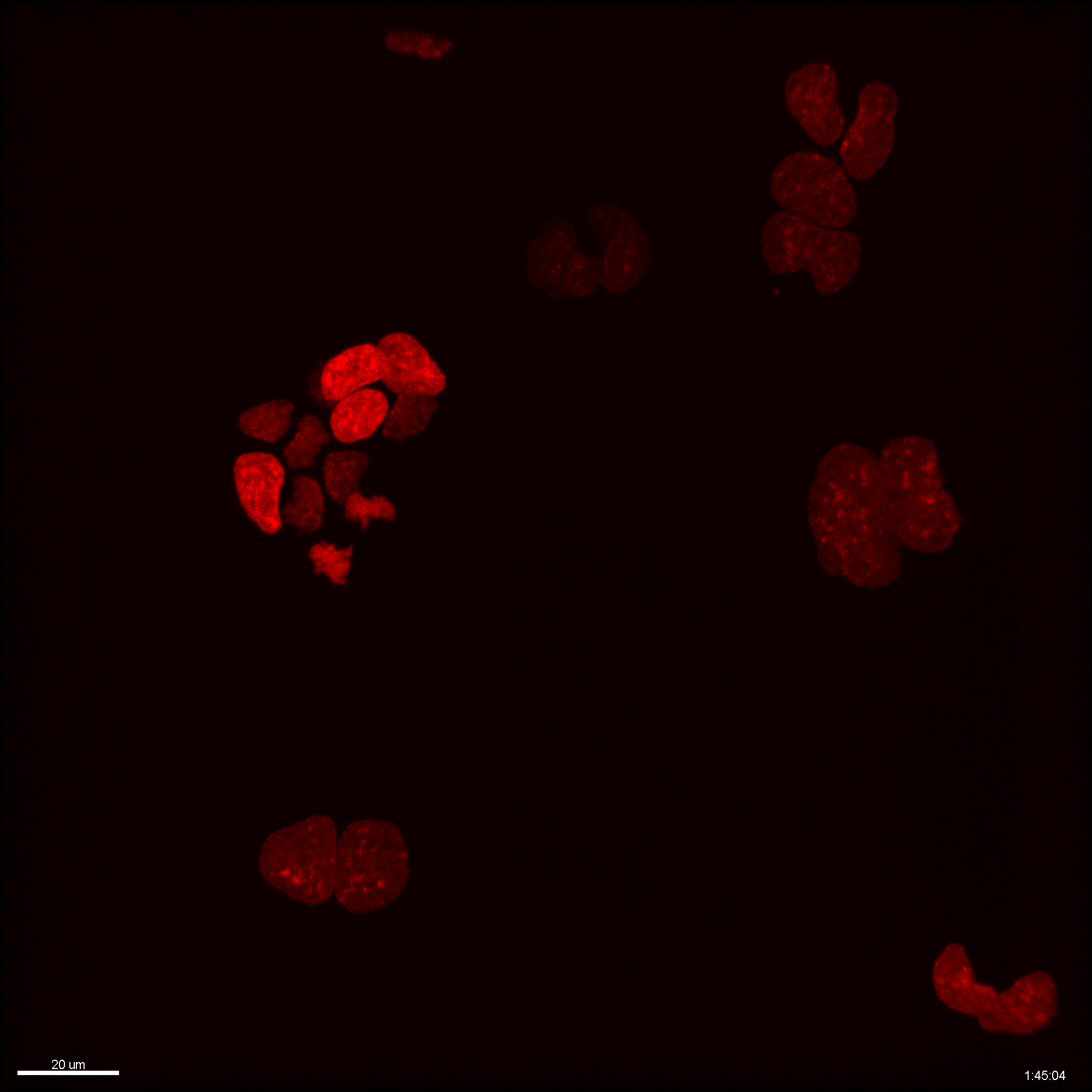

Supplement: Supplementary file 16 — Source data Fig. 2 [file 44320_2026_188_MOESM16_ESM.zip › Figure 2/2B/Live cell imaging Chr1+2' 15 min.tif]

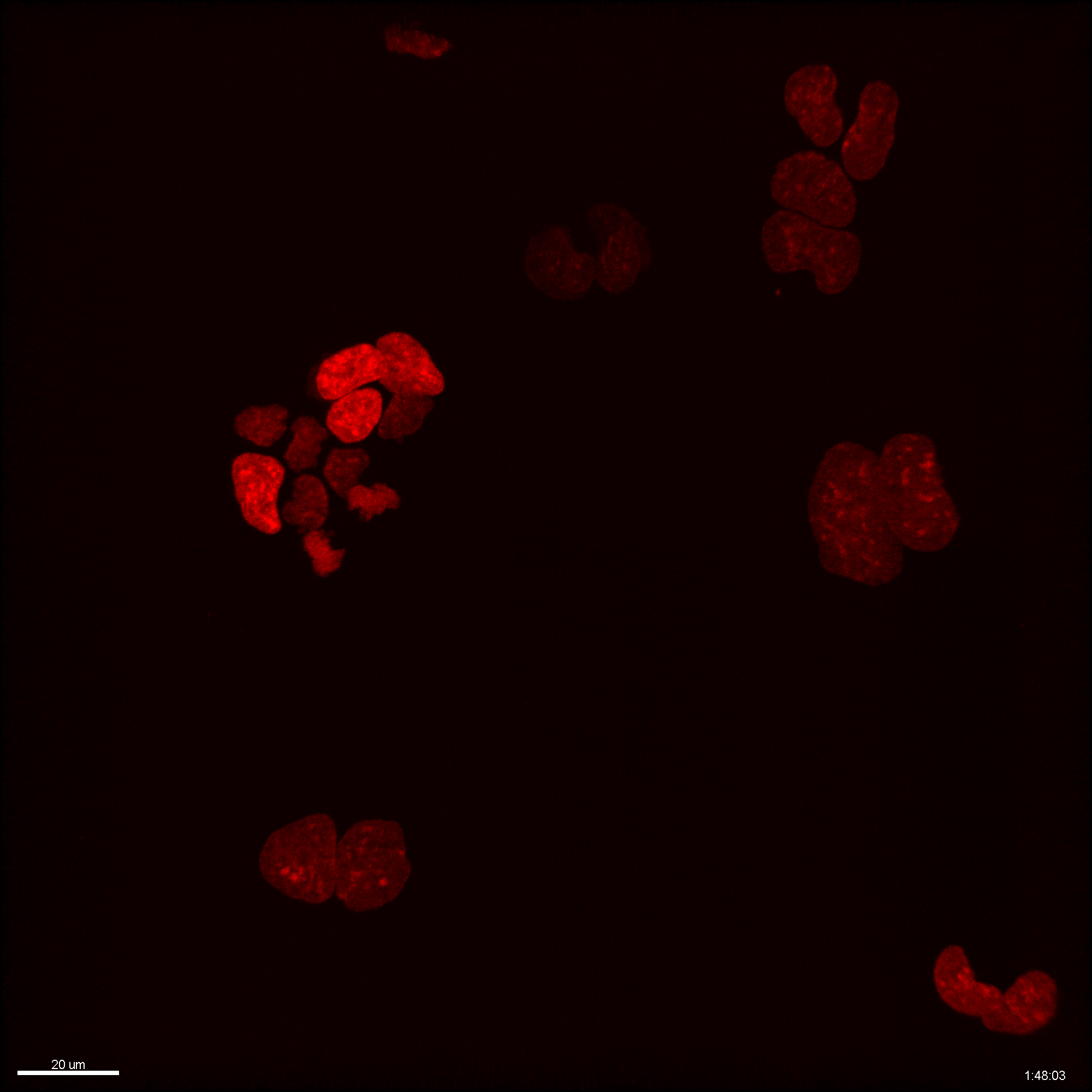

Supplement: Supplementary file 16 — Source data Fig. 2 [file 44320_2026_188_MOESM16_ESM.zip › Figure 2/2B/Live cell imaging Chr1+2' 18 min.tif]

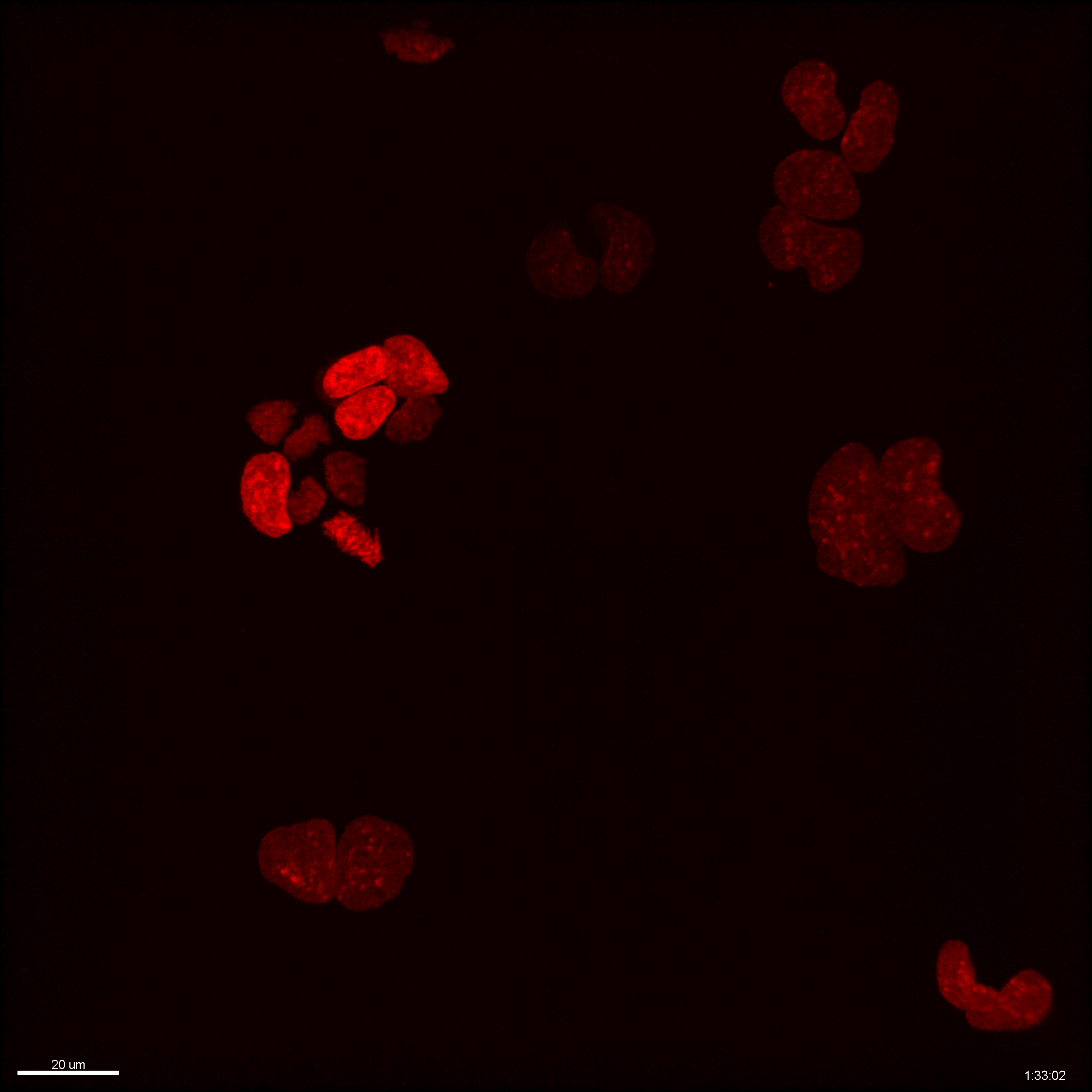

Supplement: Supplementary file 16 — Source data Fig. 2 [file 44320_2026_188_MOESM16_ESM.zip › Figure 2/2B/Live cell imaging Chr1+2' 3 min.tif]

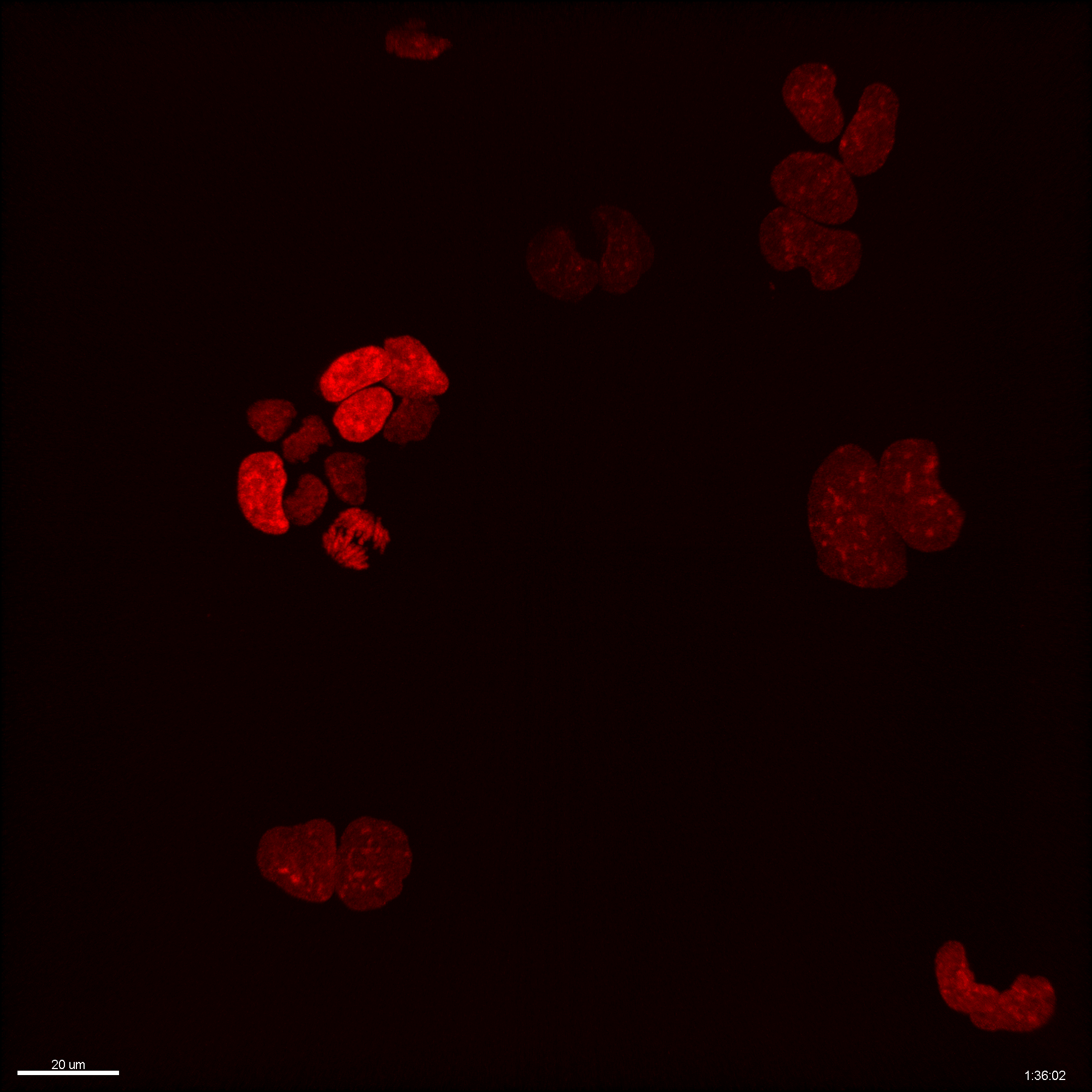

Supplement: Supplementary file 16 — Source data Fig. 2 [file 44320_2026_188_MOESM16_ESM.zip › Figure 2/2B/Live cell imaging Chr1+2' 6 min.tif]

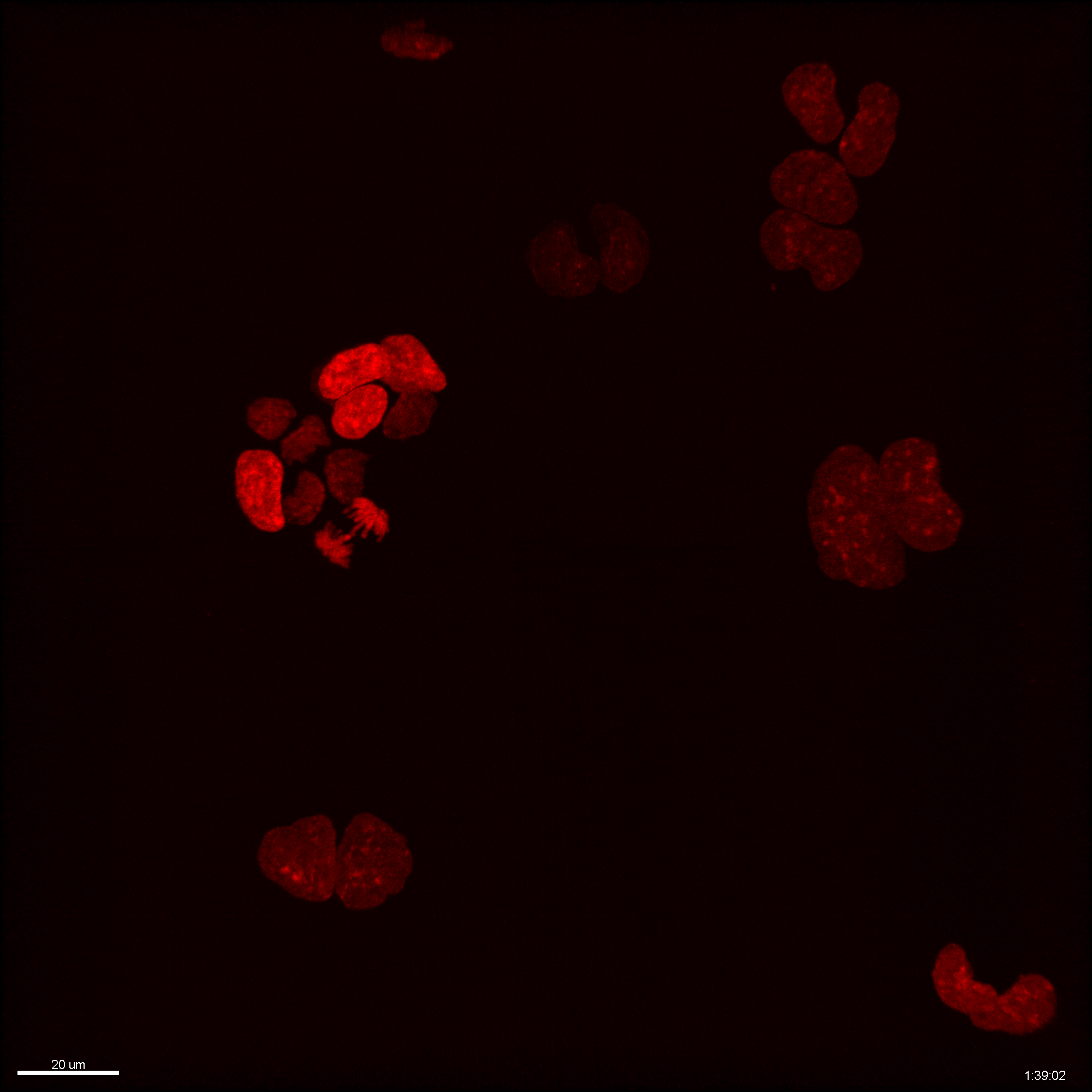

Supplement: Supplementary file 16 — Source data Fig. 2 [file 44320_2026_188_MOESM16_ESM.zip › Figure 2/2B/Live cell imaging Chr1+2' 9 min.tif]

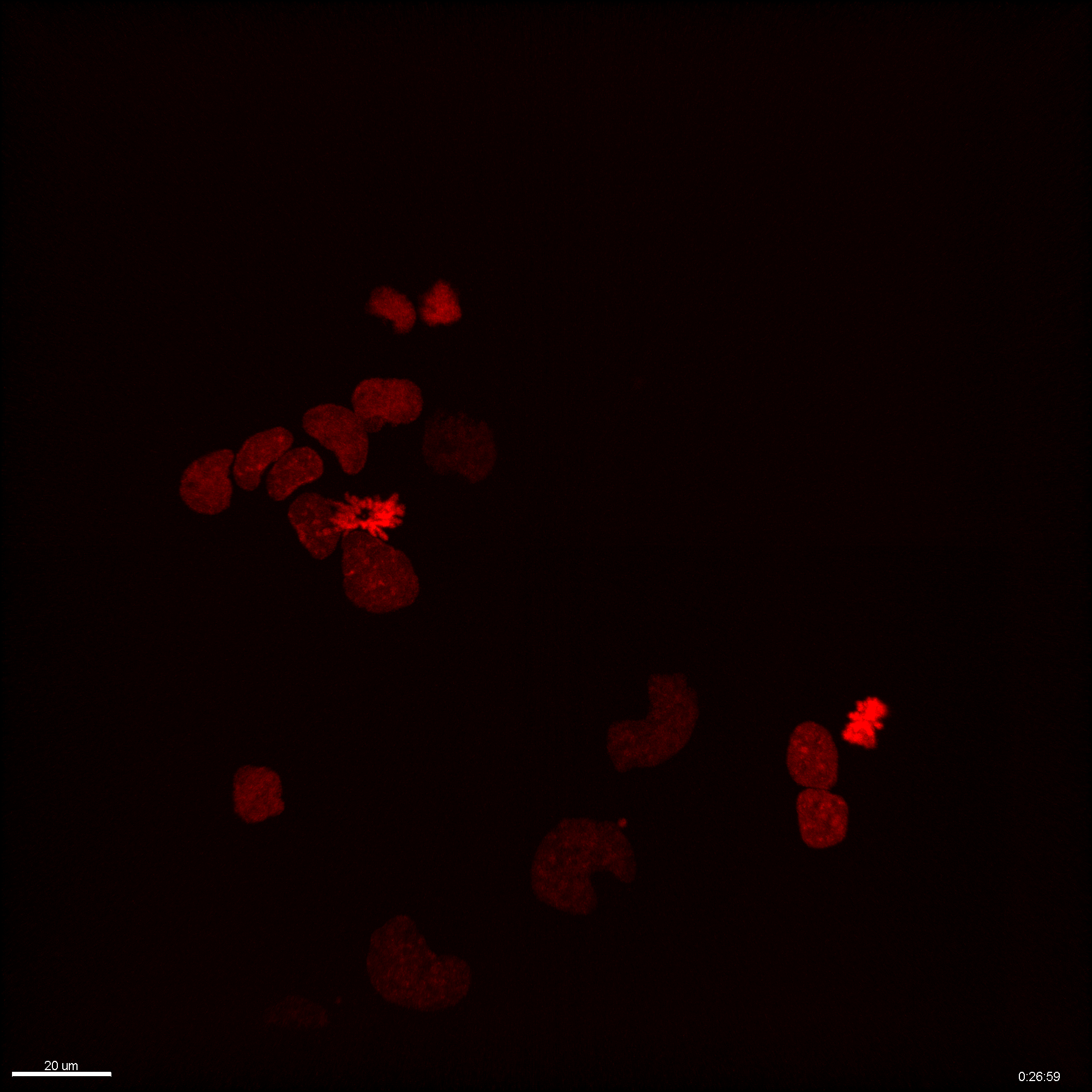

Supplement: Supplementary file 16 — Source data Fig. 2 [file 44320_2026_188_MOESM16_ESM.zip › Figure 2/2B/Live cell imaging Chr2+1 0 min.tif]

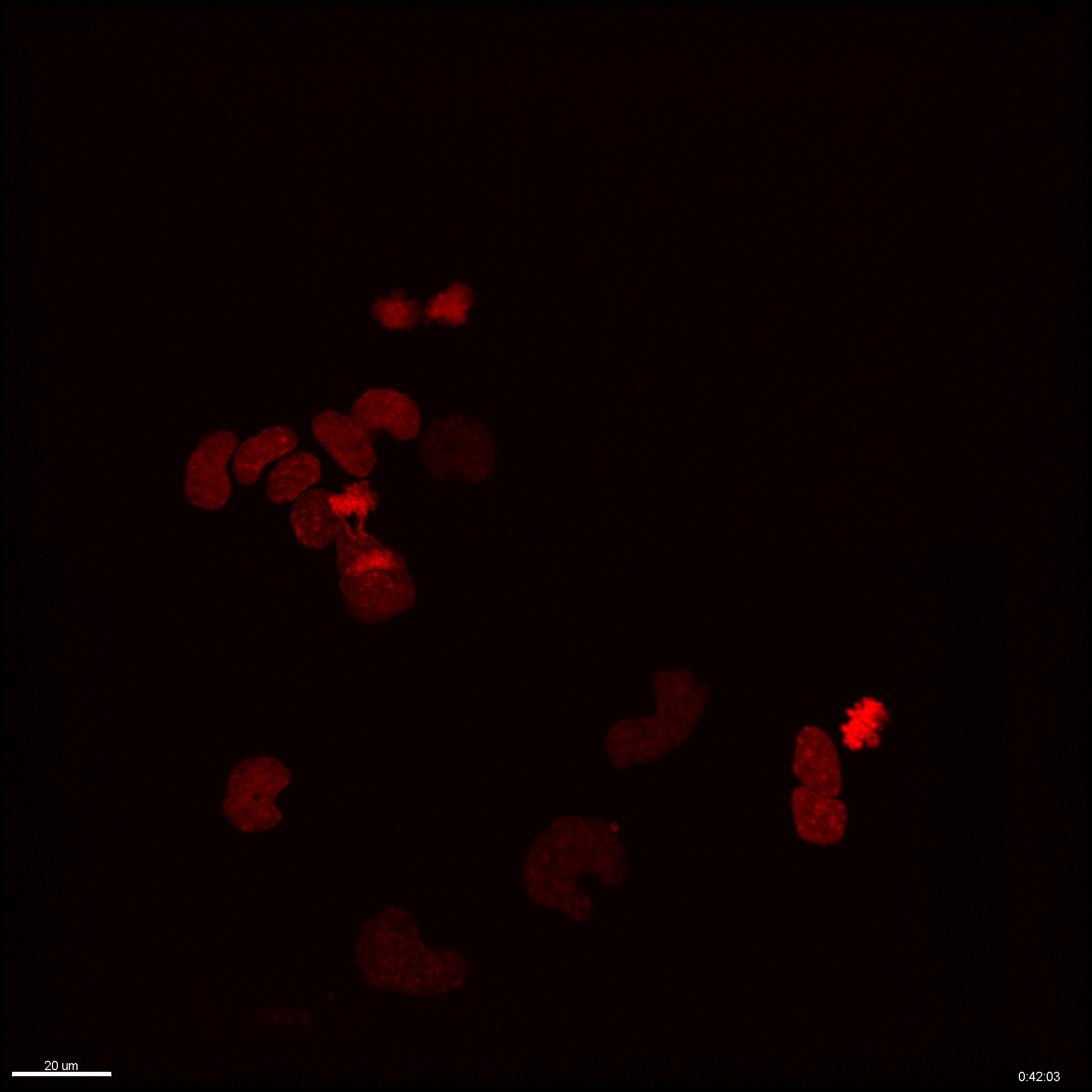

Supplement: Supplementary file 16 — Source data Fig. 2 [file 44320_2026_188_MOESM16_ESM.zip › Figure 2/2B/Live cell imaging Chr2+1 15 min.tif]

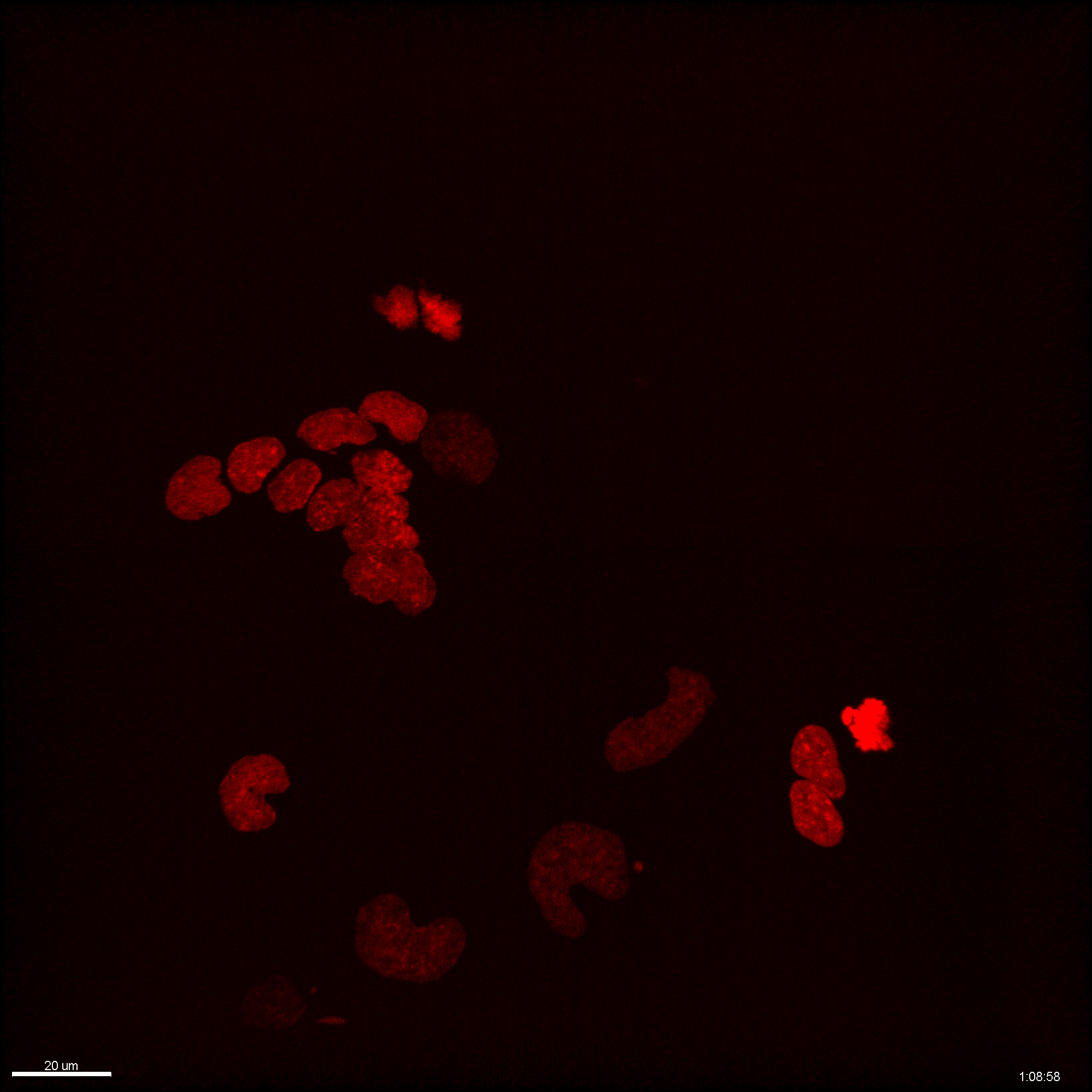

Supplement: Supplementary file 16 — Source data Fig. 2 [file 44320_2026_188_MOESM16_ESM.zip › Figure 2/2B/Live cell imaging Chr2+1 42 min.tif]

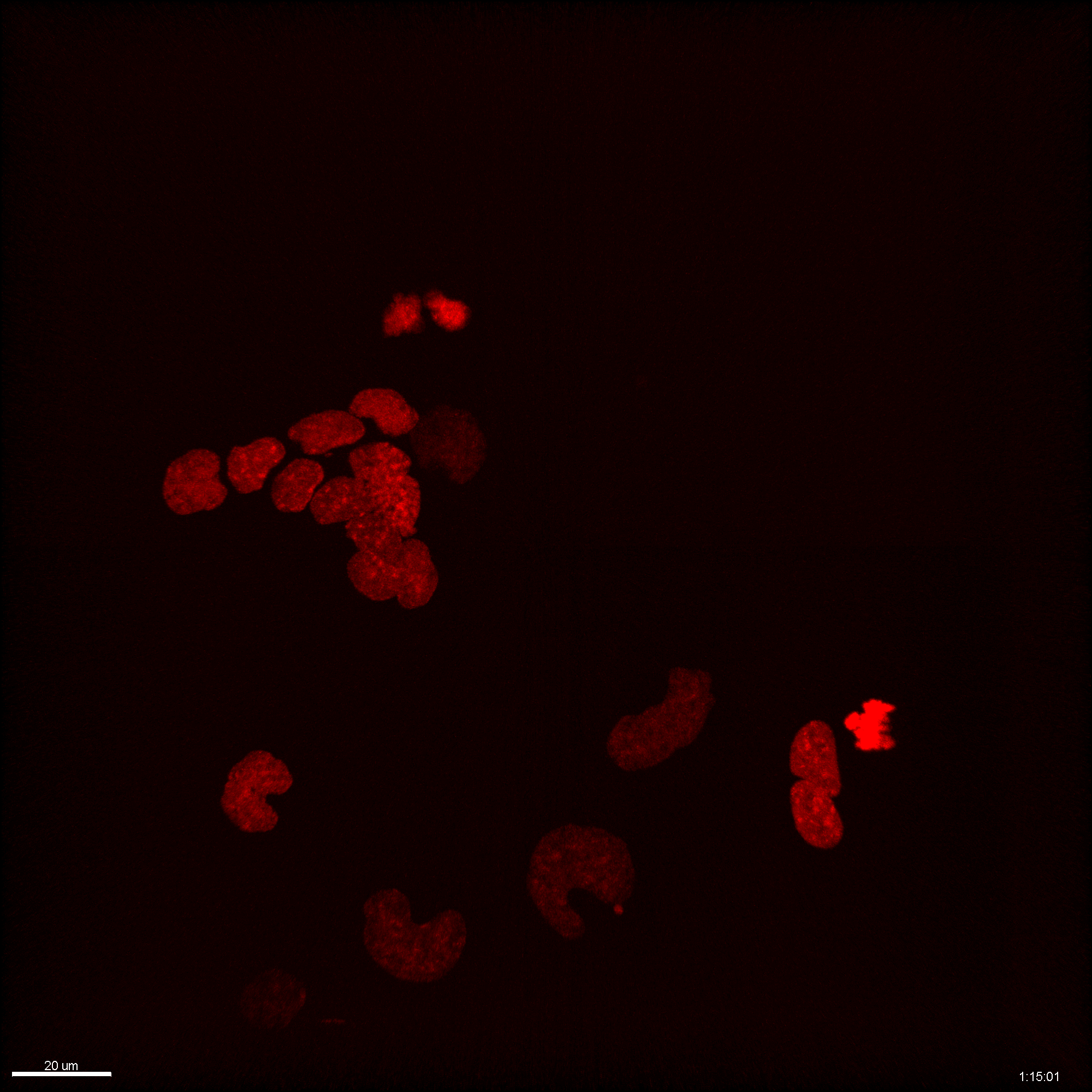

Supplement: Supplementary file 16 — Source data Fig. 2 [file 44320_2026_188_MOESM16_ESM.zip › Figure 2/2B/Live cell imaging Chr2+1 48 min.tif]

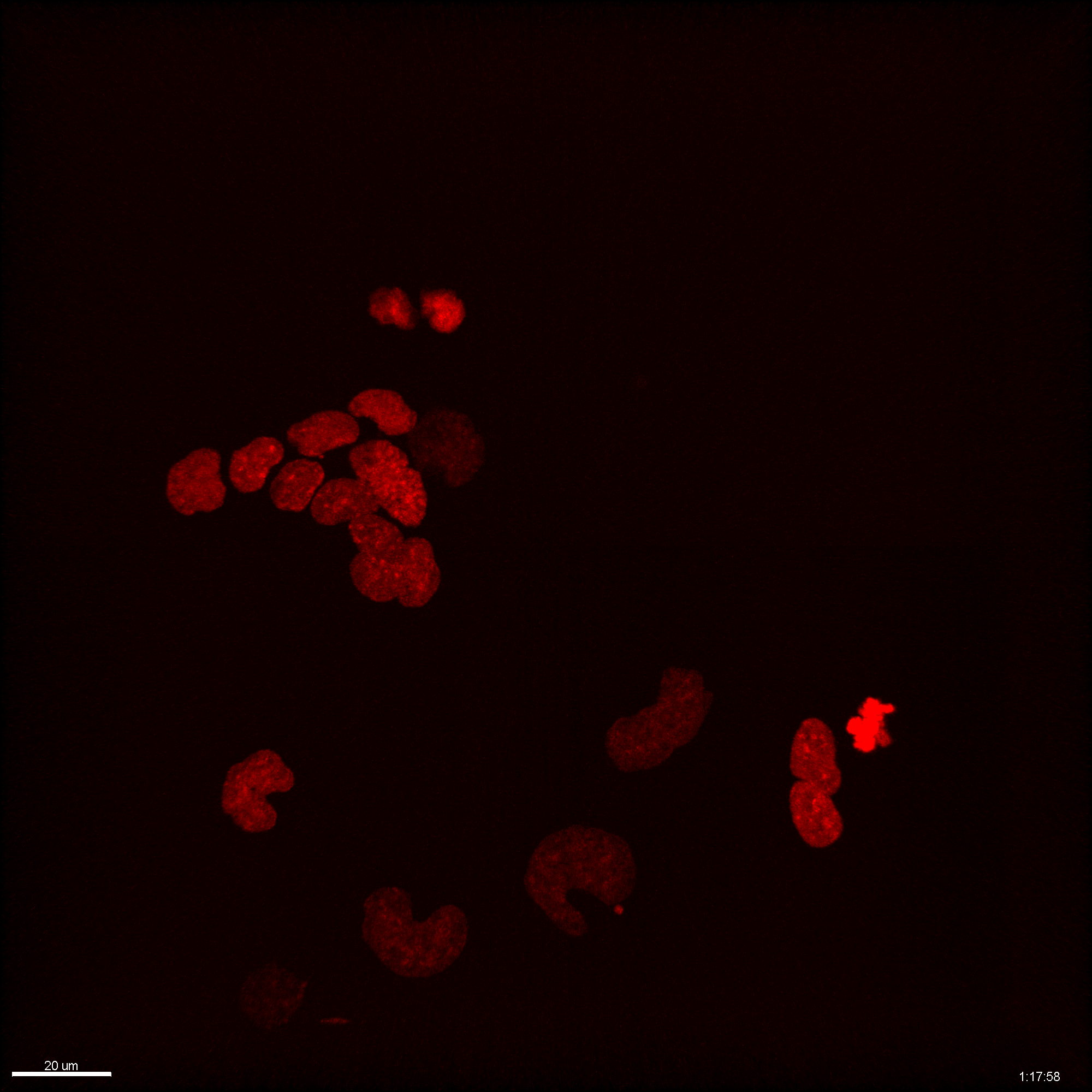

Supplement: Supplementary file 16 — Source data Fig. 2 [file 44320_2026_188_MOESM16_ESM.zip › Figure 2/2B/Live cell imaging Chr2+1 51 min.tif]

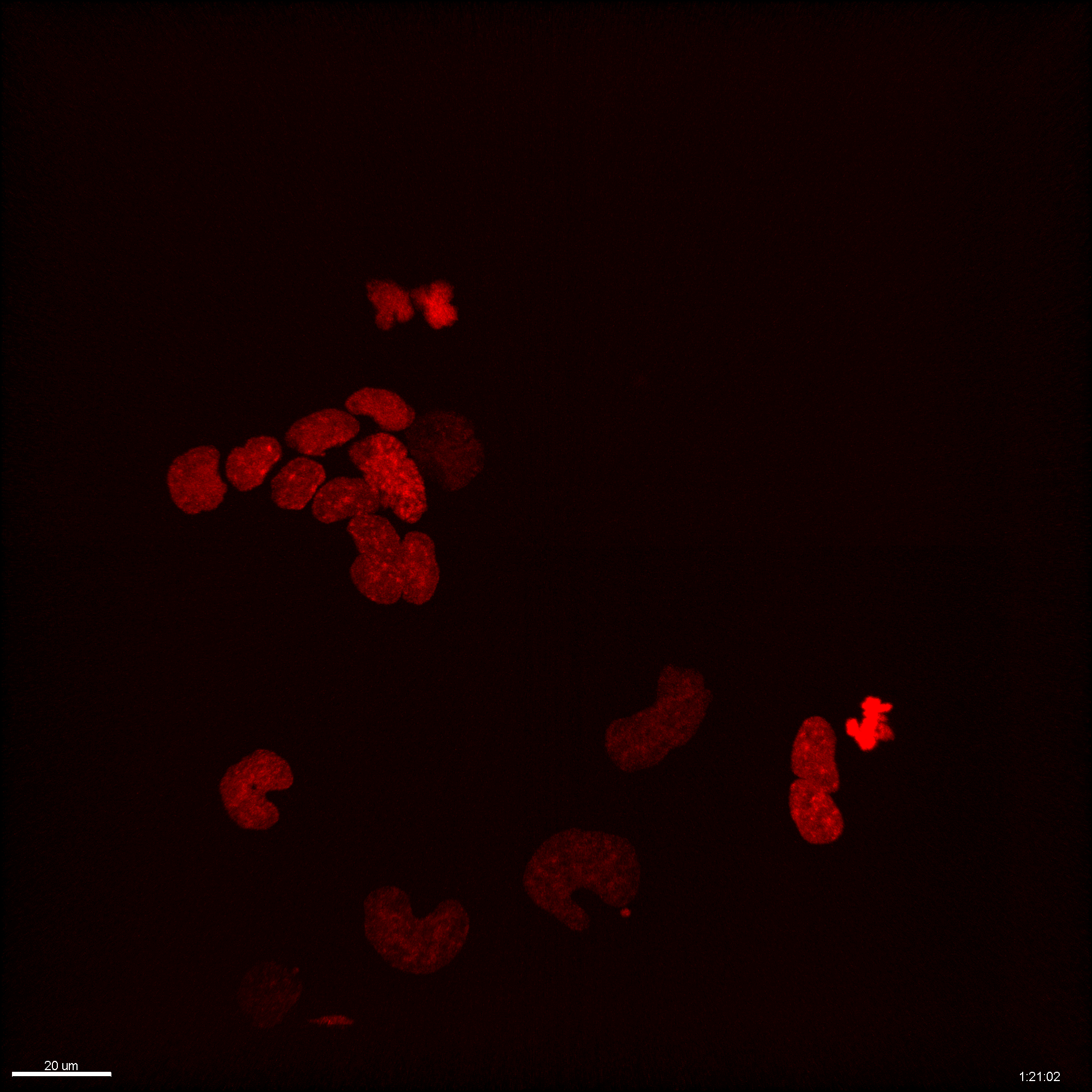

Supplement: Supplementary file 16 — Source data Fig. 2 [file 44320_2026_188_MOESM16_ESM.zip › Figure 2/2B/Live cell imaging Chr2+1 54 min.tif]

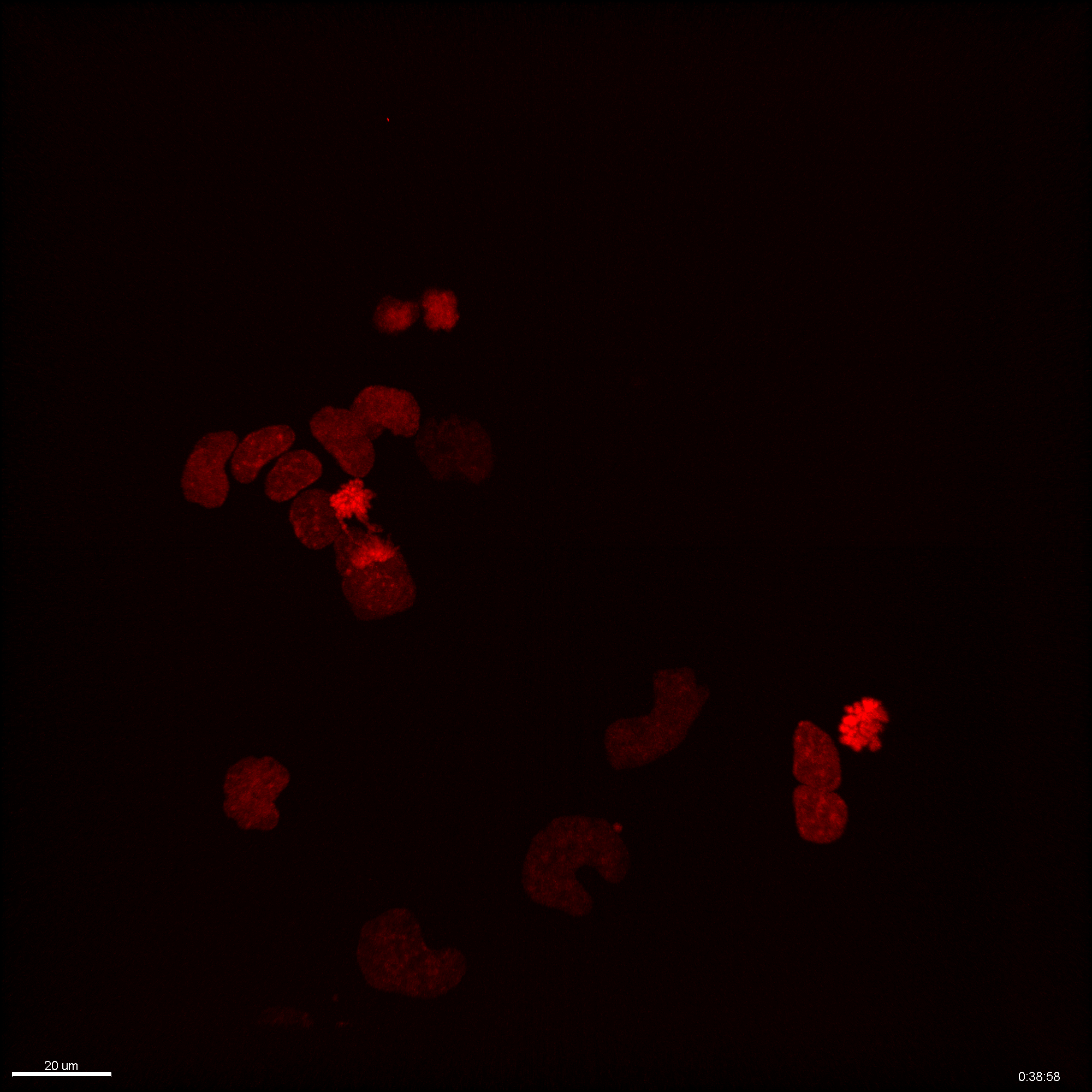

Supplement: Supplementary file 16 — Source data Fig. 2 [file 44320_2026_188_MOESM16_ESM.zip › Figure 2/2B/Live cell imaging Chr2+1 9 min.tif]

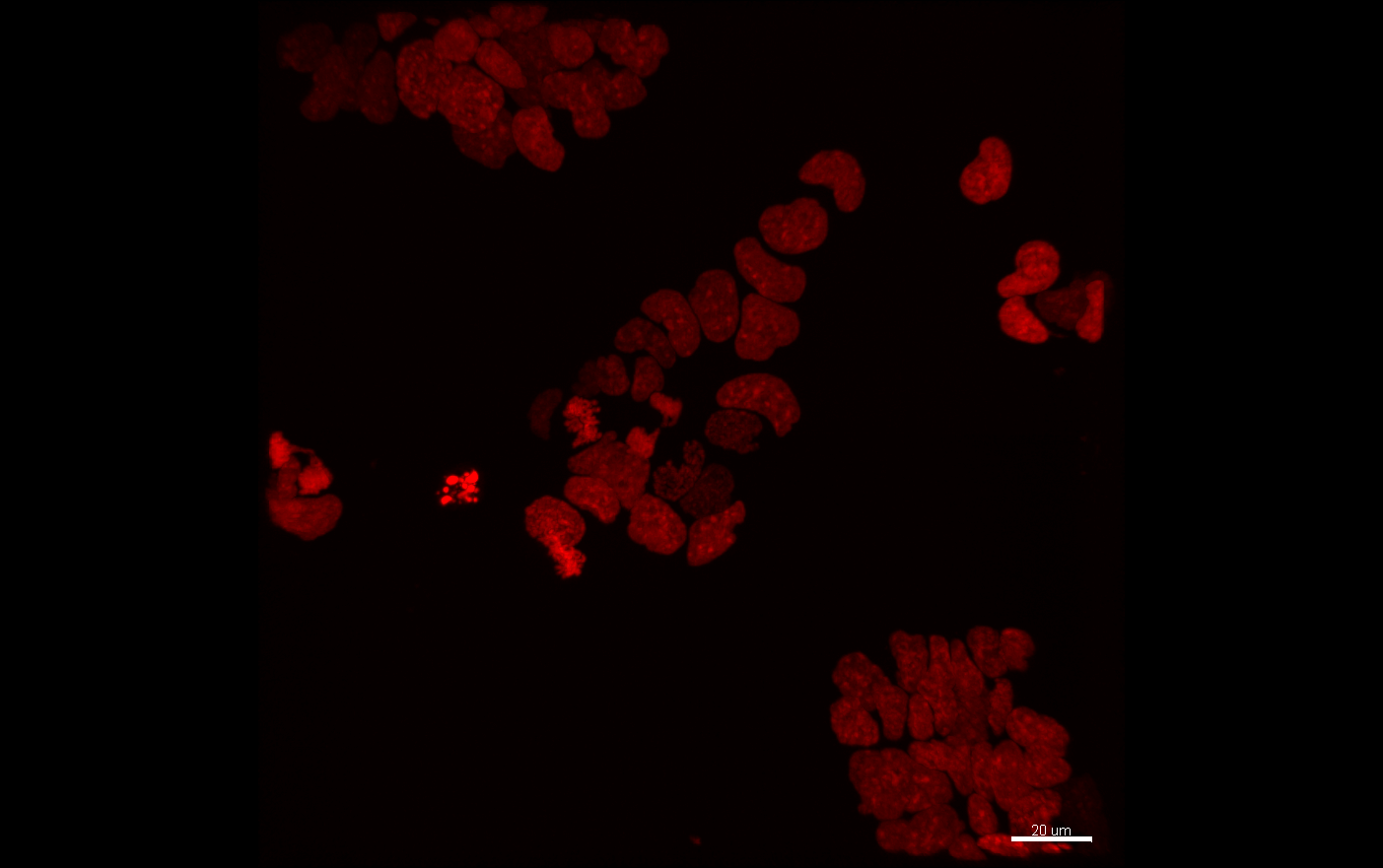

Supplement: Supplementary file 16 — Source data Fig. 2 [file 44320_2026_188_MOESM16_ESM.zip › Figure 2/2B/Live cell imaging Chr2+3 0 min.tif]

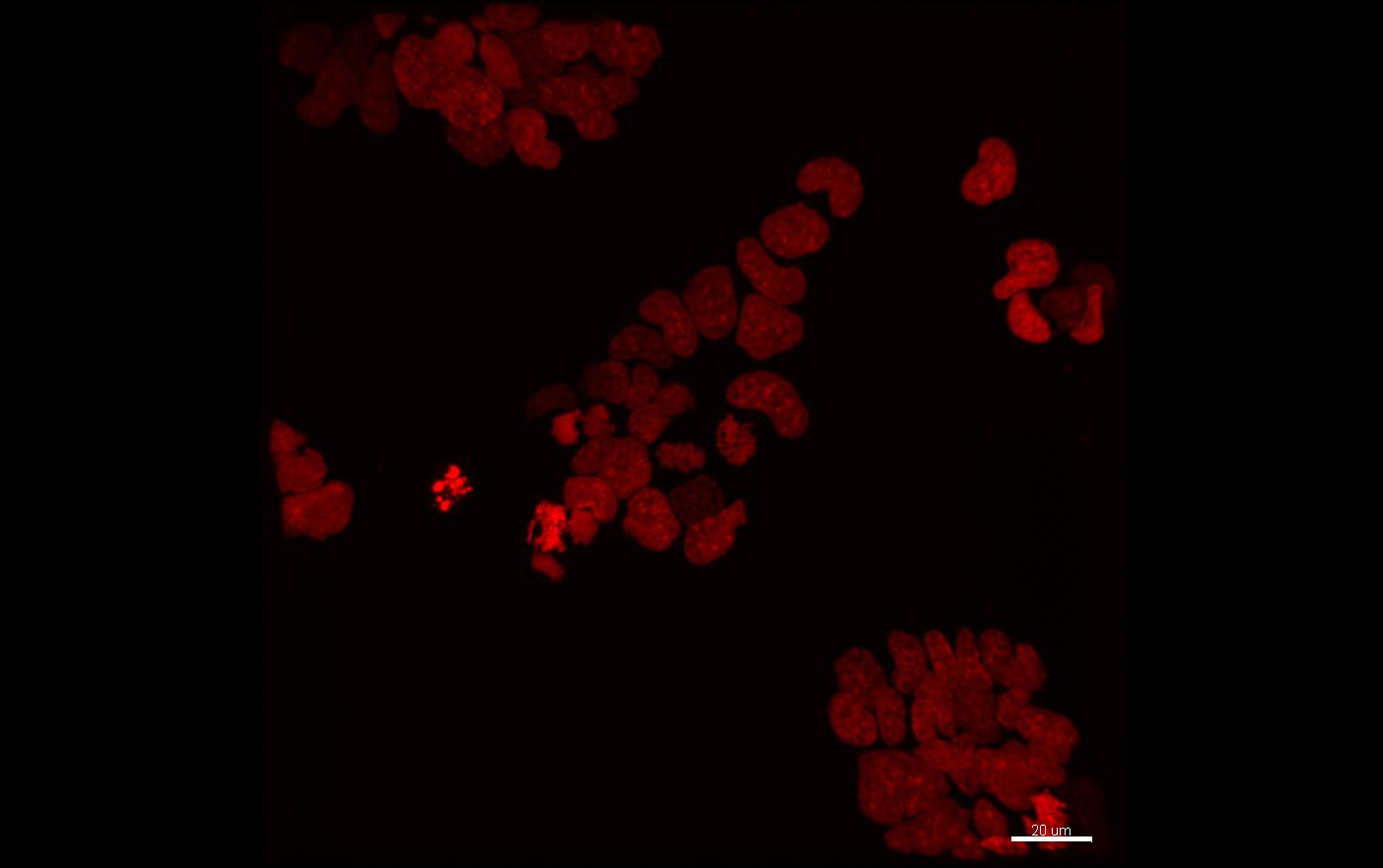

Supplement: Supplementary file 16 — Source data Fig. 2 [file 44320_2026_188_MOESM16_ESM.zip › Figure 2/2B/Live cell imaging Chr2+3 12 min.tif]

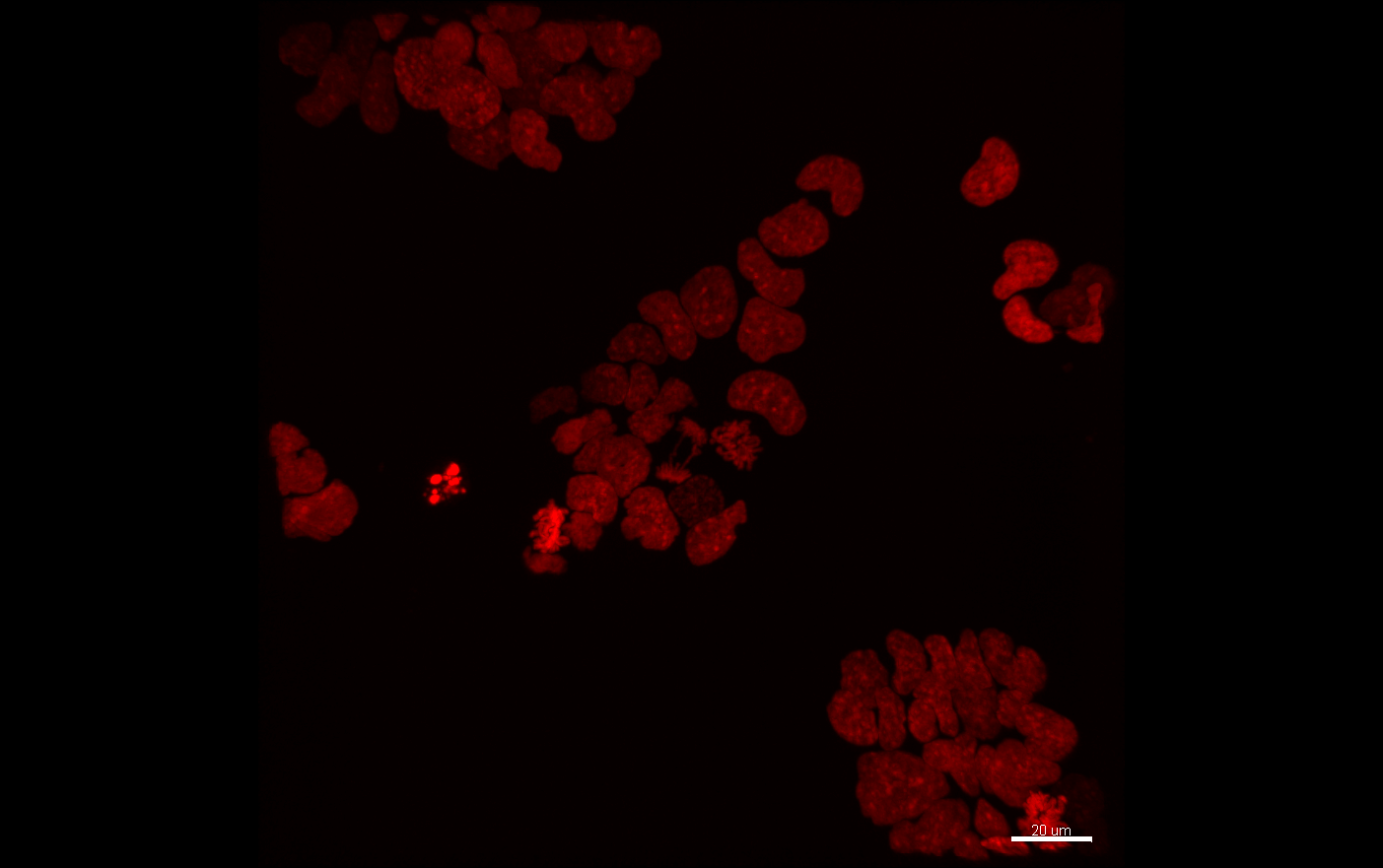

Supplement: Supplementary file 16 — Source data Fig. 2 [file 44320_2026_188_MOESM16_ESM.zip › Figure 2/2B/Live cell imaging Chr2+3 15 min.tif]

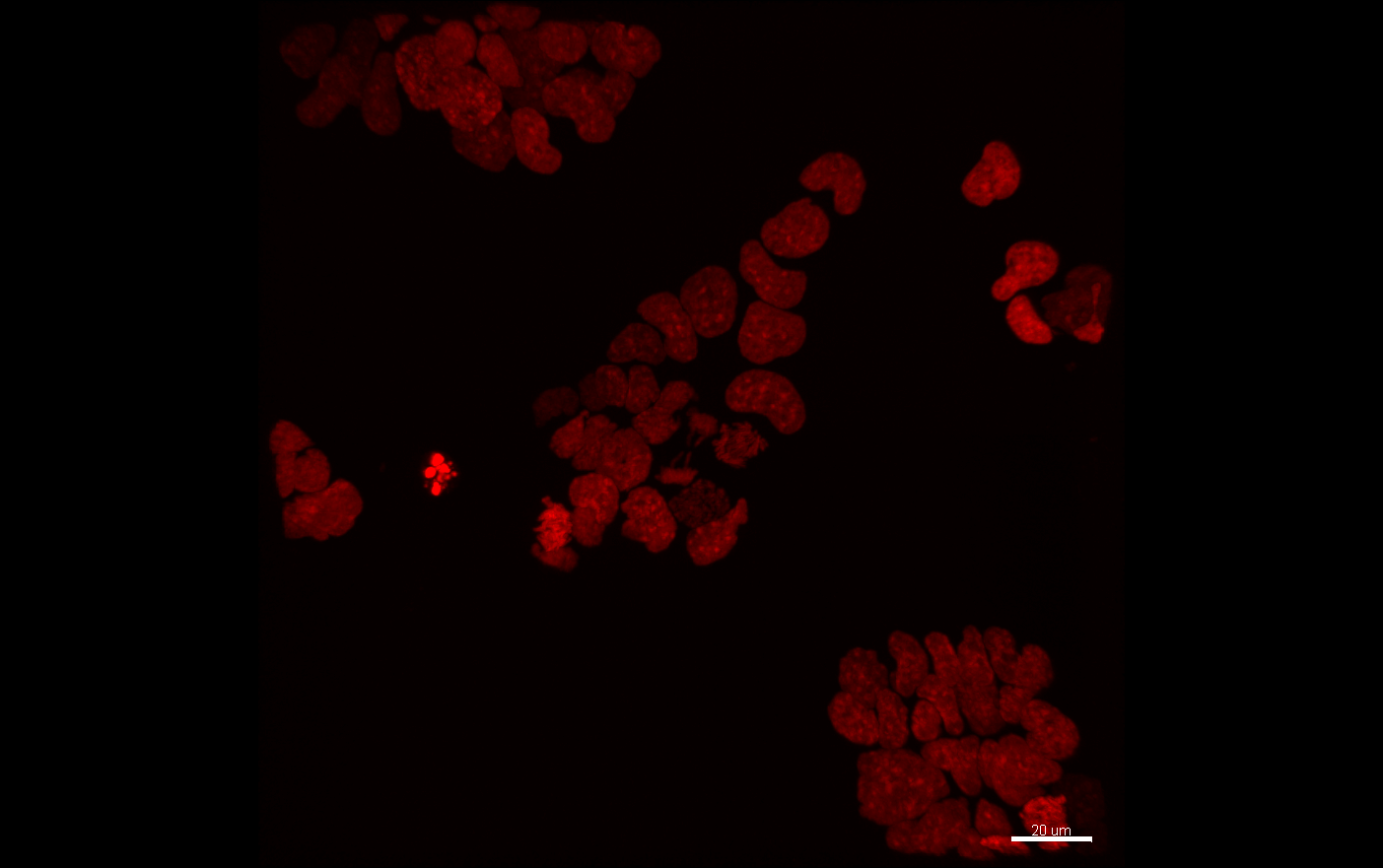

Supplement: Supplementary file 16 — Source data Fig. 2 [file 44320_2026_188_MOESM16_ESM.zip › Figure 2/2B/Live cell imaging Chr2+3 18 min.tif]

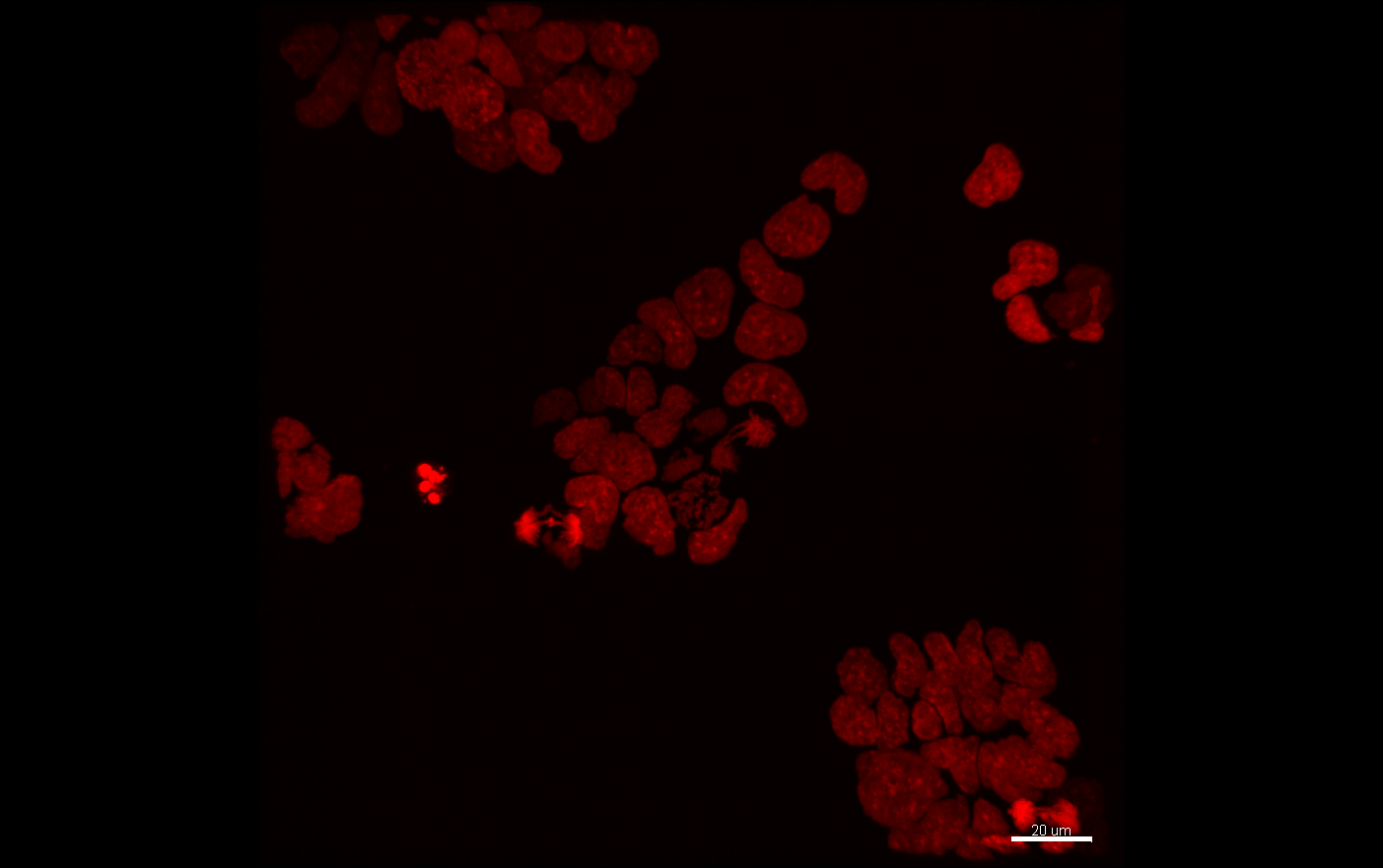

Supplement: Supplementary file 16 — Source data Fig. 2 [file 44320_2026_188_MOESM16_ESM.zip › Figure 2/2B/Live cell imaging Chr2+3 21 min.tif]

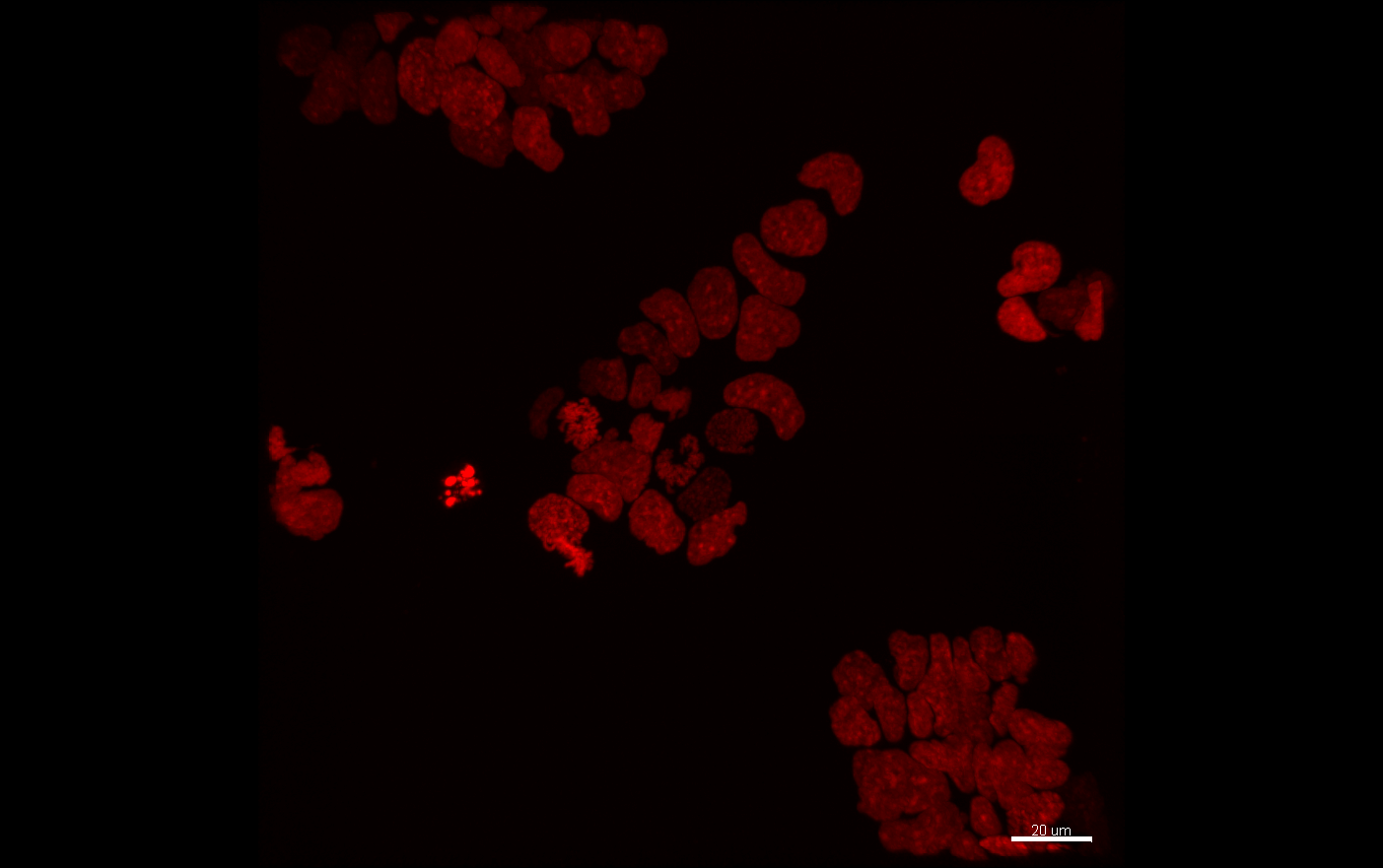

Supplement: Supplementary file 16 — Source data Fig. 2 [file 44320_2026_188_MOESM16_ESM.zip › Figure 2/2B/Live cell imaging Chr2+3 3 min.tif]

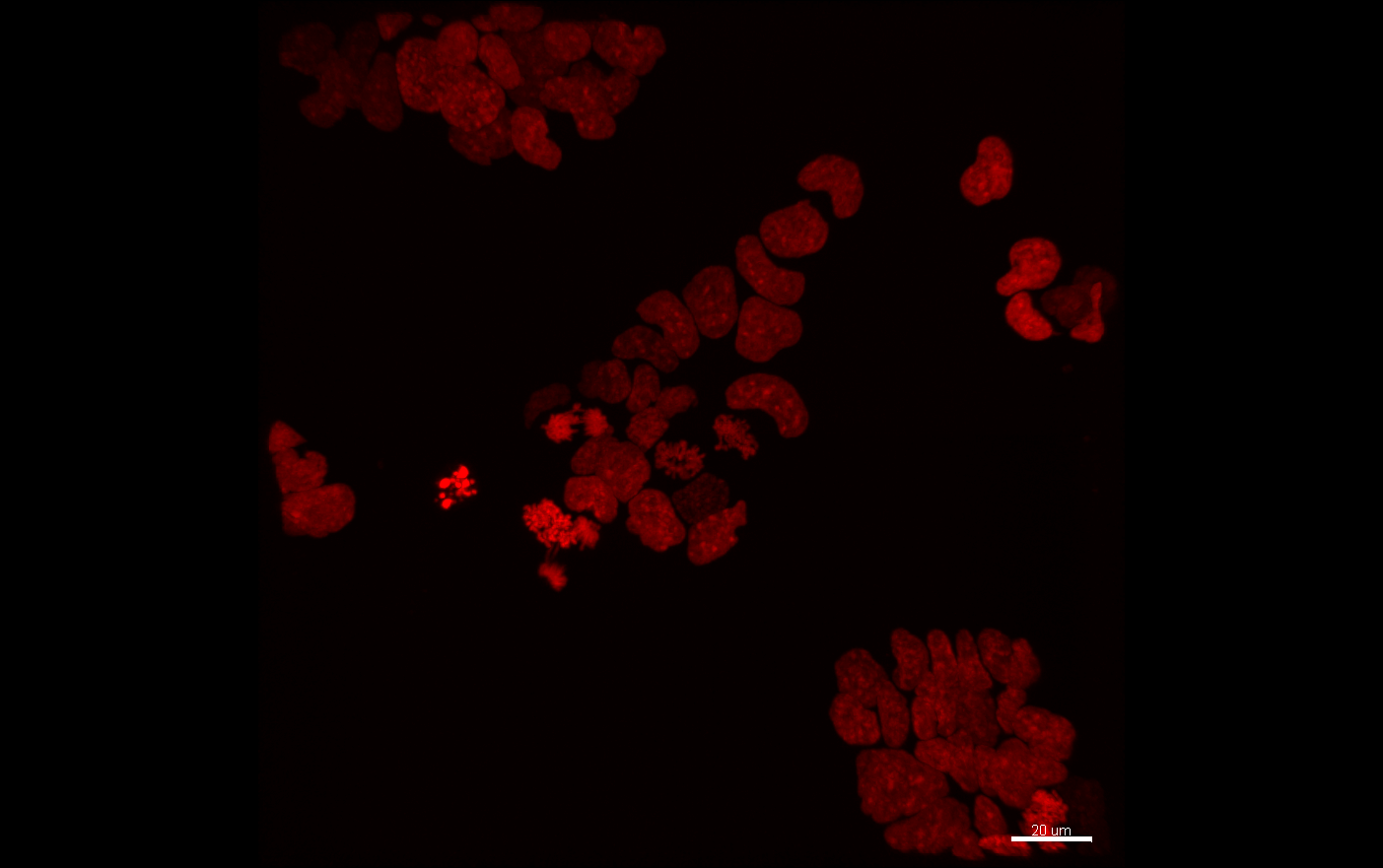

Supplement: Supplementary file 16 — Source data Fig. 2 [file 44320_2026_188_MOESM16_ESM.zip › Figure 2/2B/Live cell imaging Chr2+3 9 min.tif]

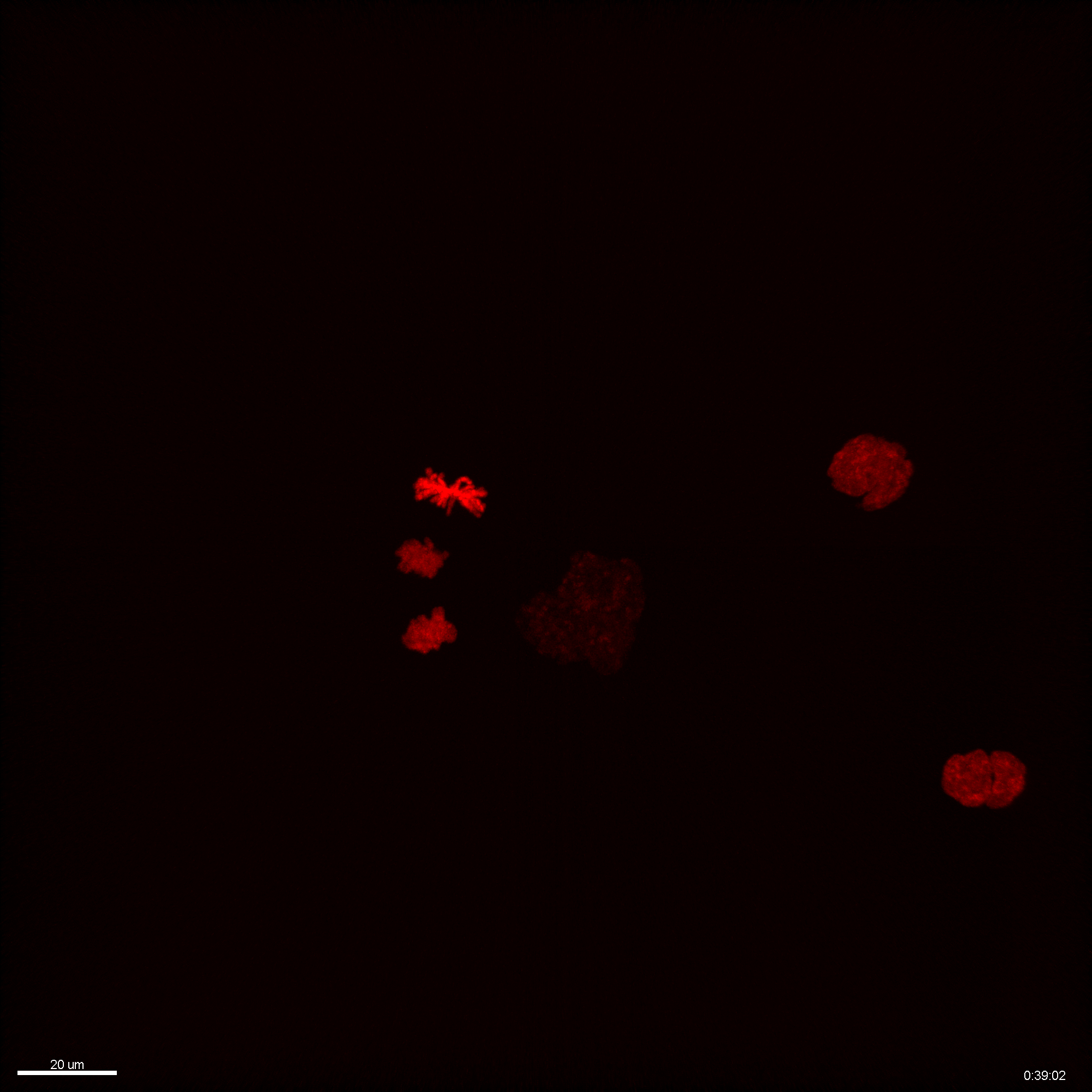

Supplement: Supplementary file 16 — Source data Fig. 2 [file 44320_2026_188_MOESM16_ESM.zip › Figure 2/2B/Live cell imaging Chr4+5 0 min.tif]

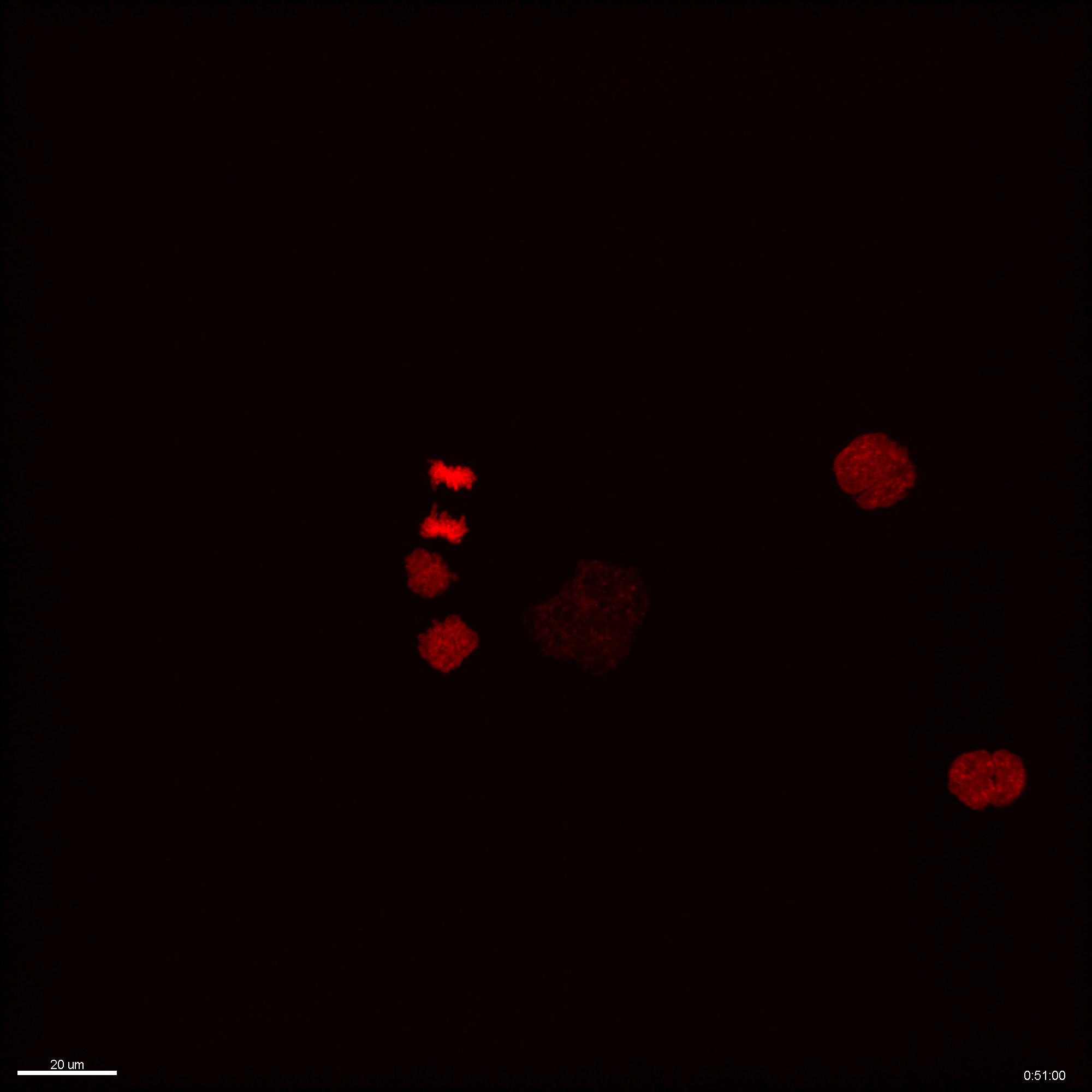

Supplement: Supplementary file 16 — Source data Fig. 2 [file 44320_2026_188_MOESM16_ESM.zip › Figure 2/2B/Live cell imaging Chr4+5 12 min.tif]

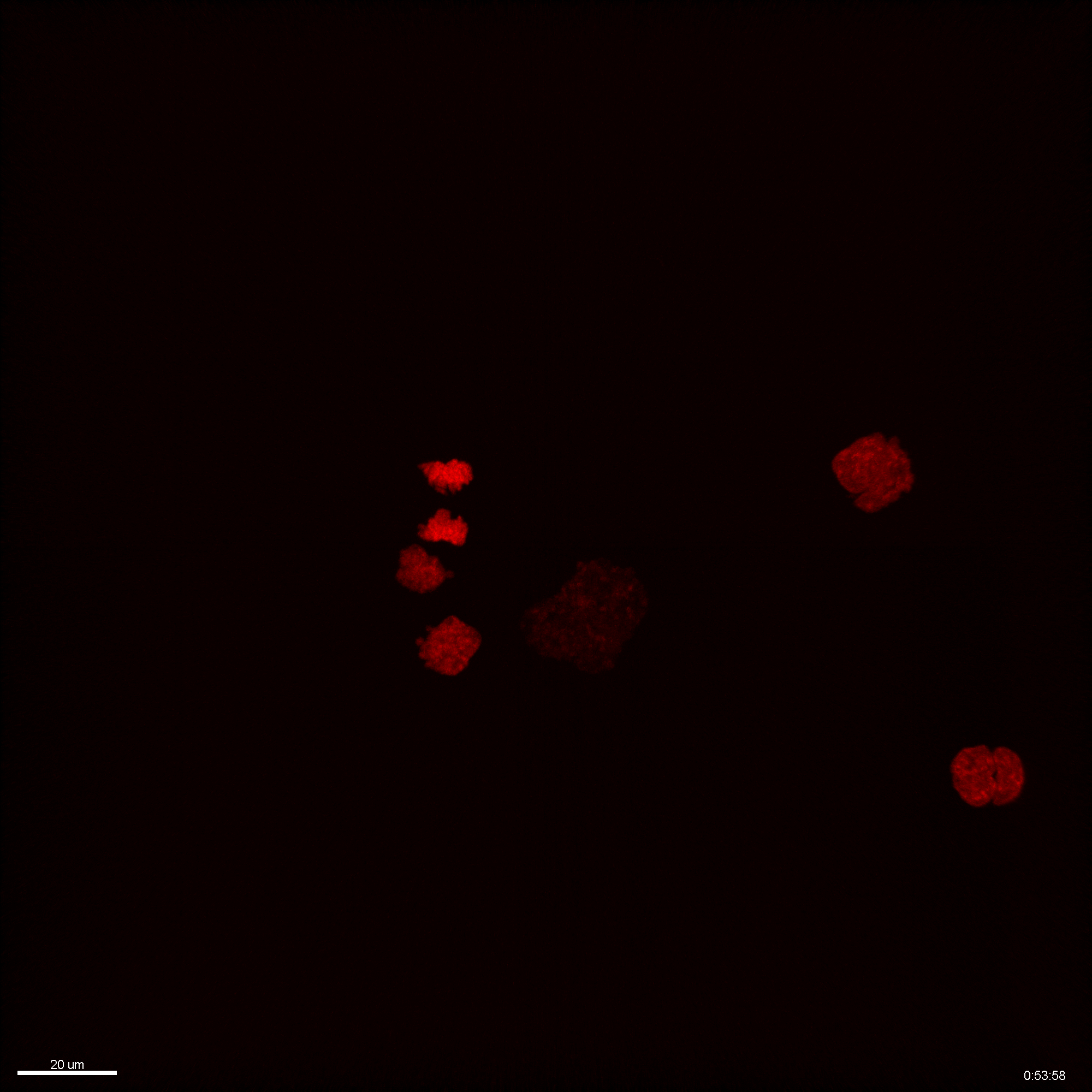

Supplement: Supplementary file 16 — Source data Fig. 2 [file 44320_2026_188_MOESM16_ESM.zip › Figure 2/2B/Live cell imaging Chr4+5 15 min.tif]

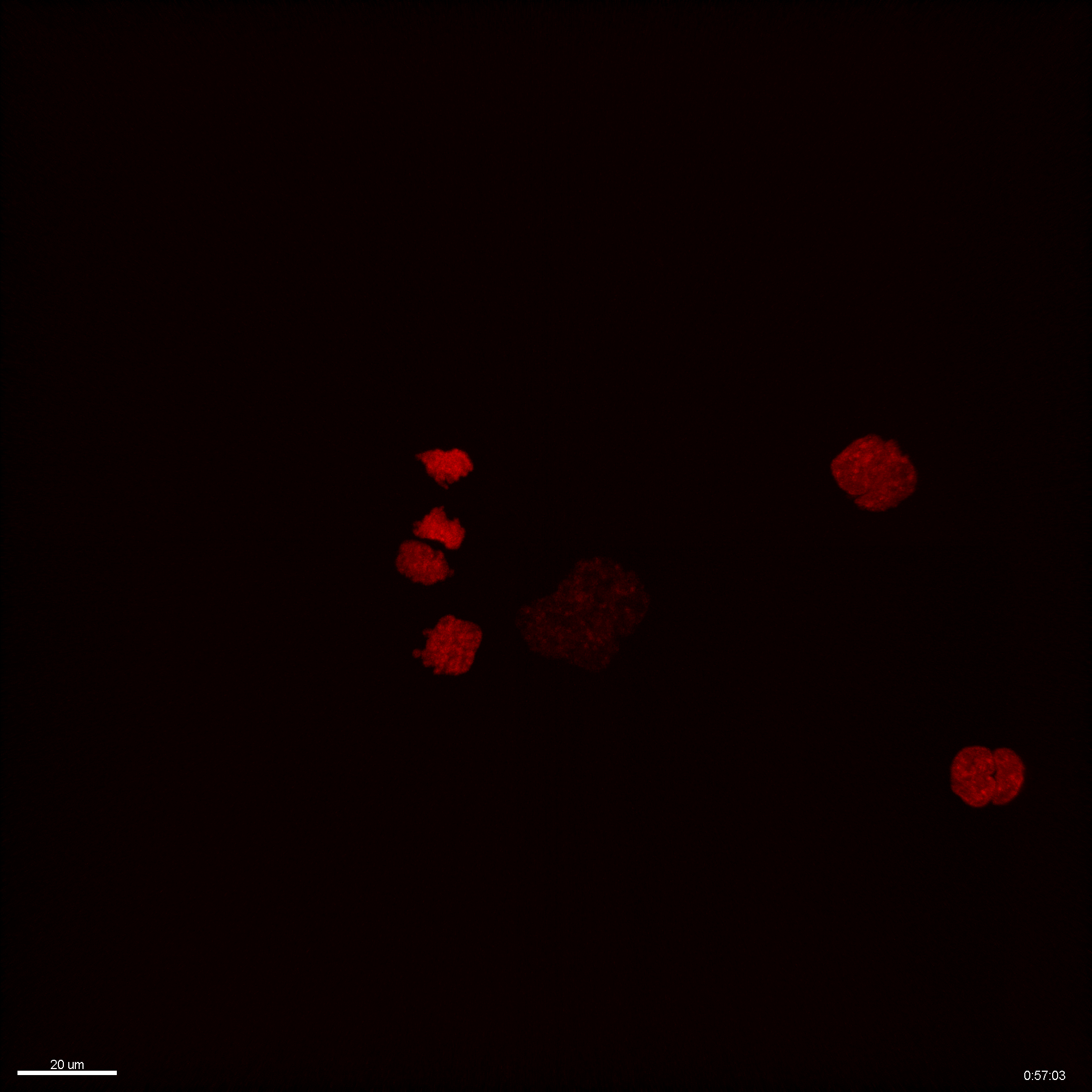

Supplement: Supplementary file 16 — Source data Fig. 2 [file 44320_2026_188_MOESM16_ESM.zip › Figure 2/2B/Live cell imaging Chr4+5 18 min.tif]

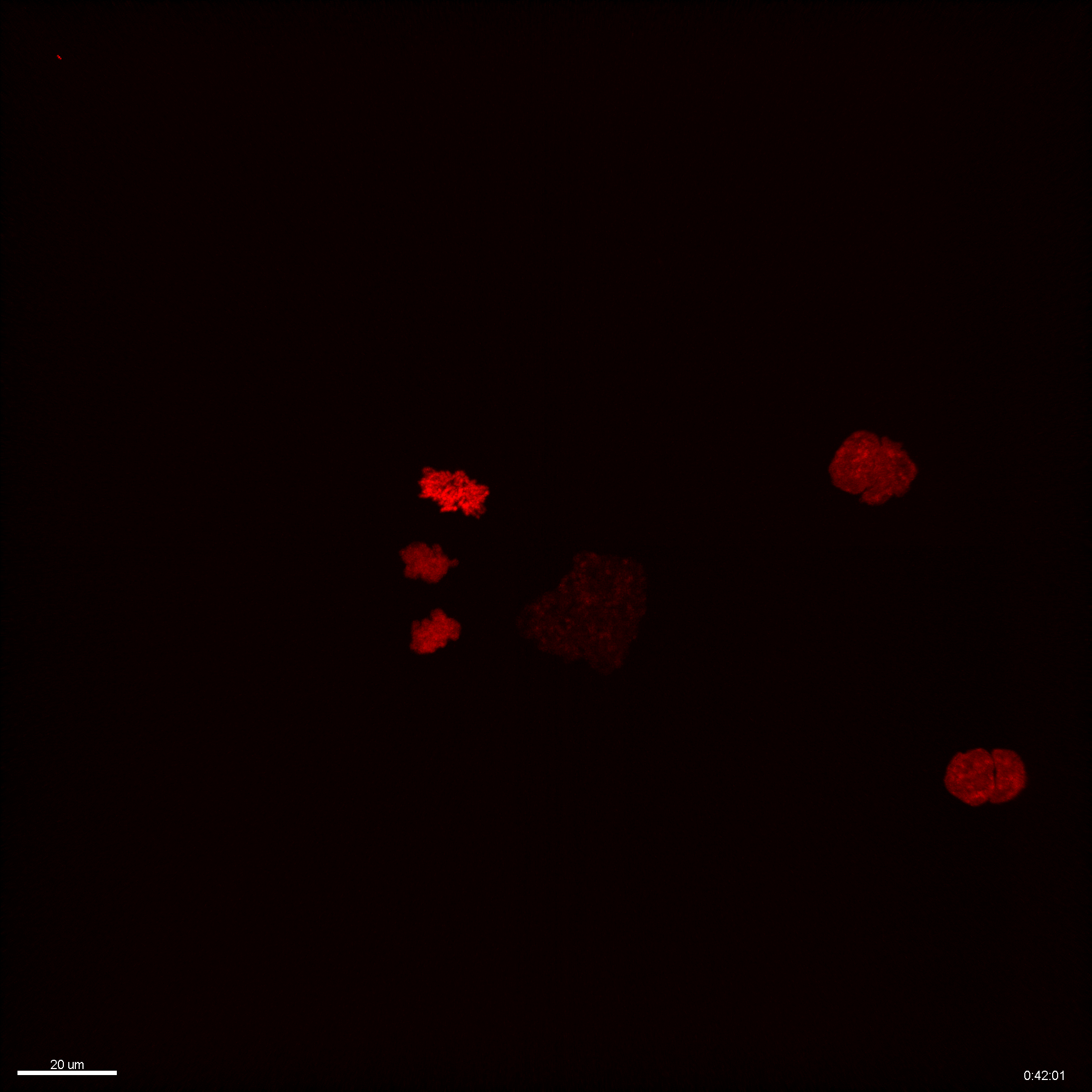

Supplement: Supplementary file 16 — Source data Fig. 2 [file 44320_2026_188_MOESM16_ESM.zip › Figure 2/2B/Live cell imaging Chr4+5 3 min.tif]

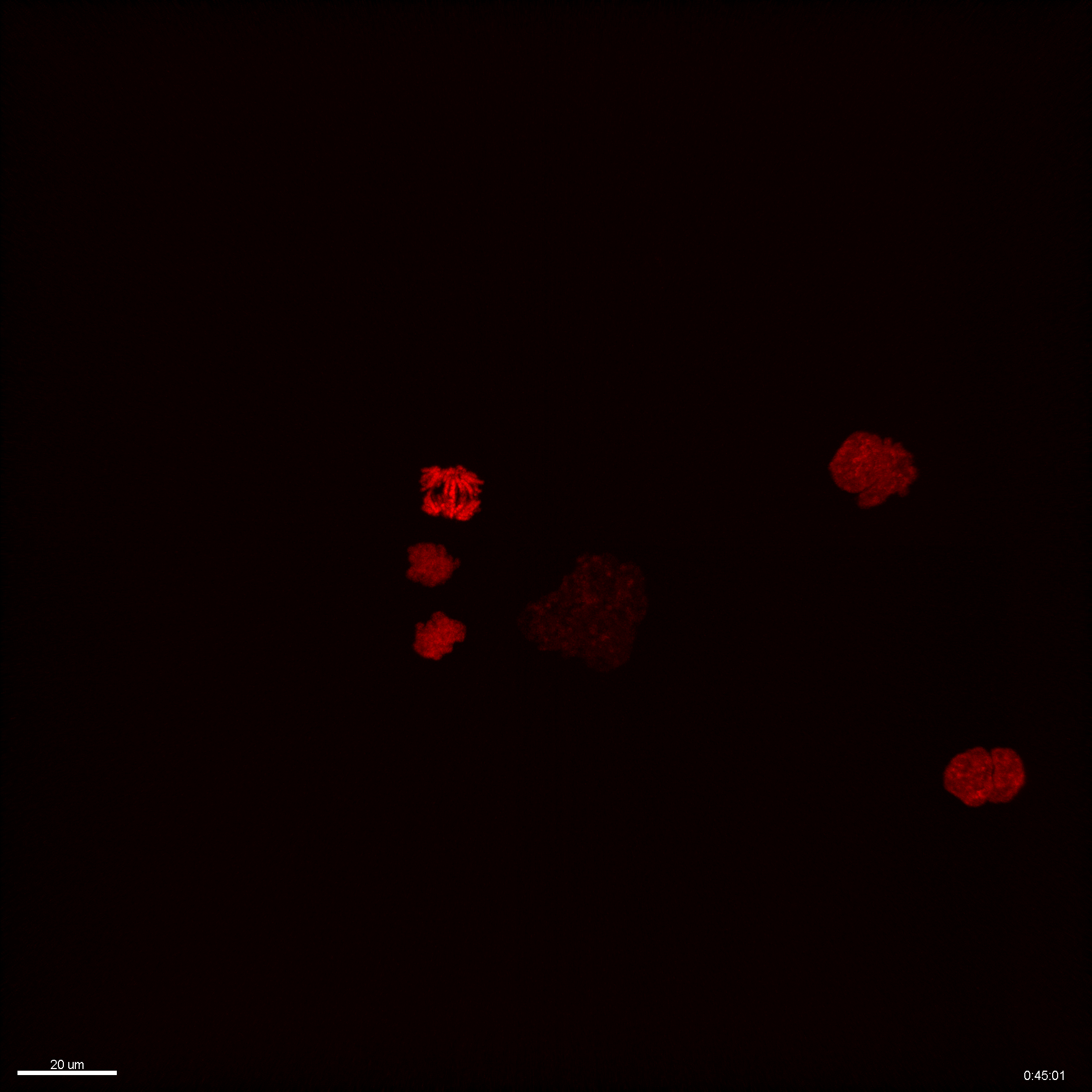

Supplement: Supplementary file 16 — Source data Fig. 2 [file 44320_2026_188_MOESM16_ESM.zip › Figure 2/2B/Live cell imaging Chr4+5 6 min.tif]

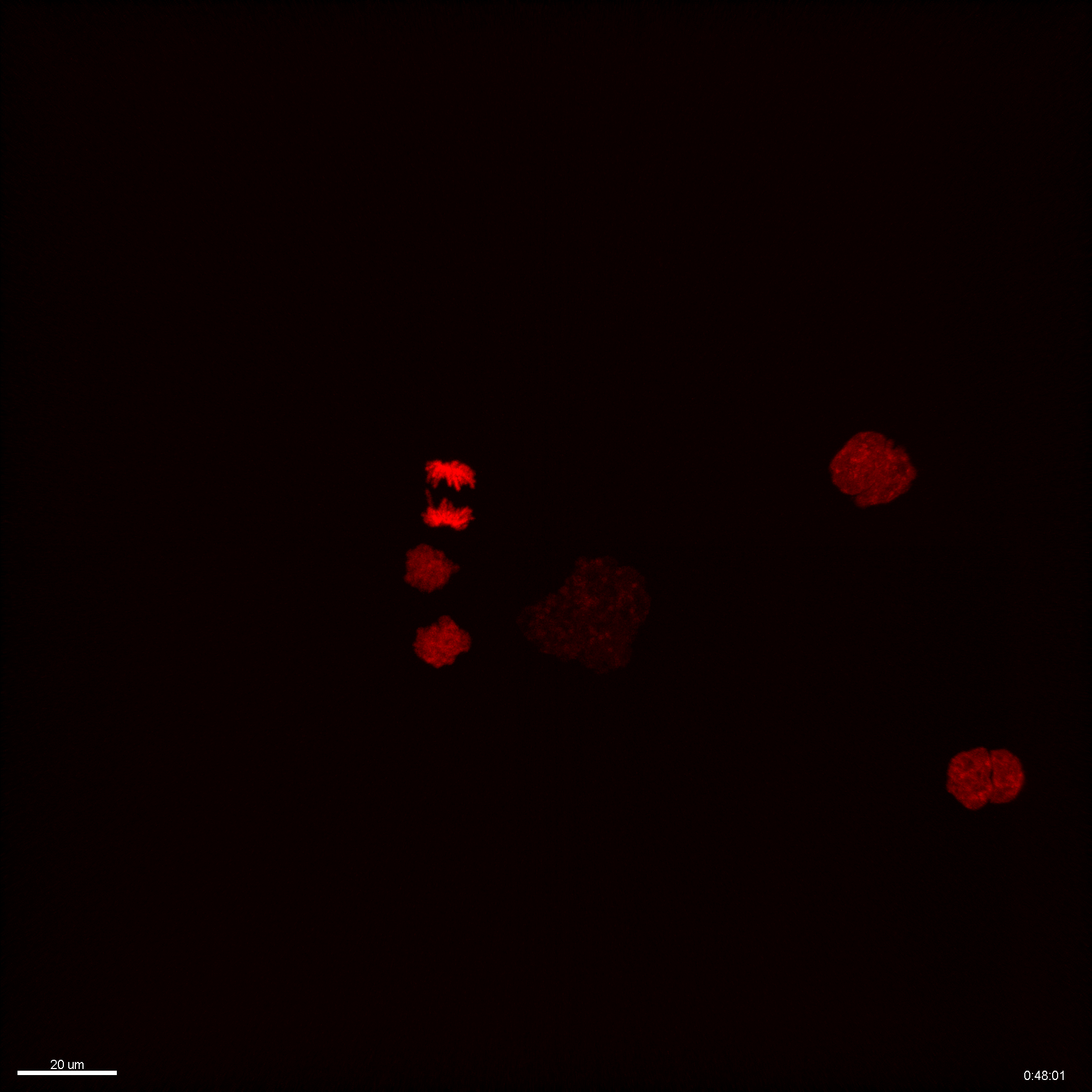

Supplement: Supplementary file 16 — Source data Fig. 2 [file 44320_2026_188_MOESM16_ESM.zip › Figure 2/2B/Live cell imaging Chr4+5 9 min.tif]

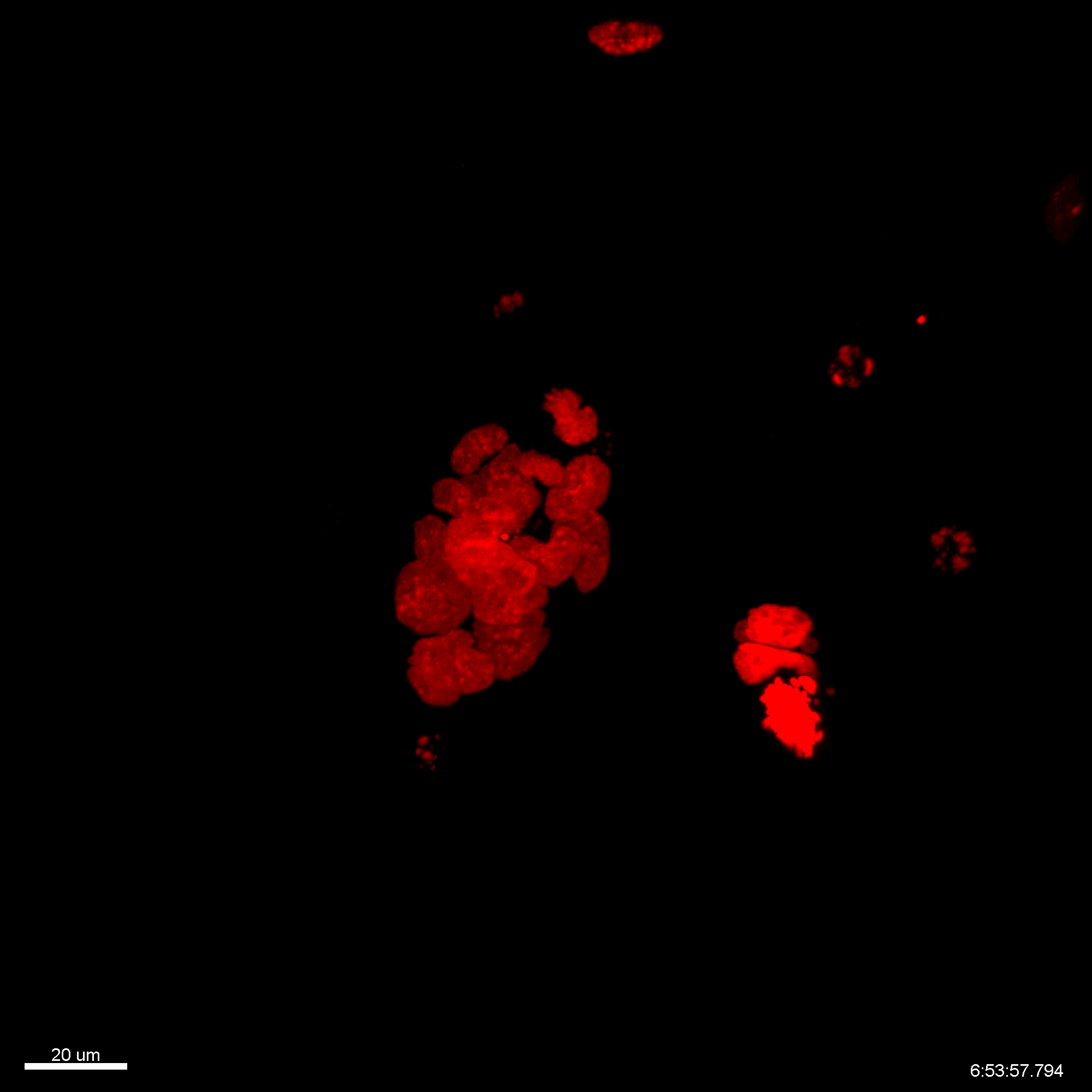

Supplement: Supplementary file 16 — Source data Fig. 2 [file 44320_2026_188_MOESM16_ESM.zip › Figure 2/2B/Live cell imaging WT 0 min.tif]

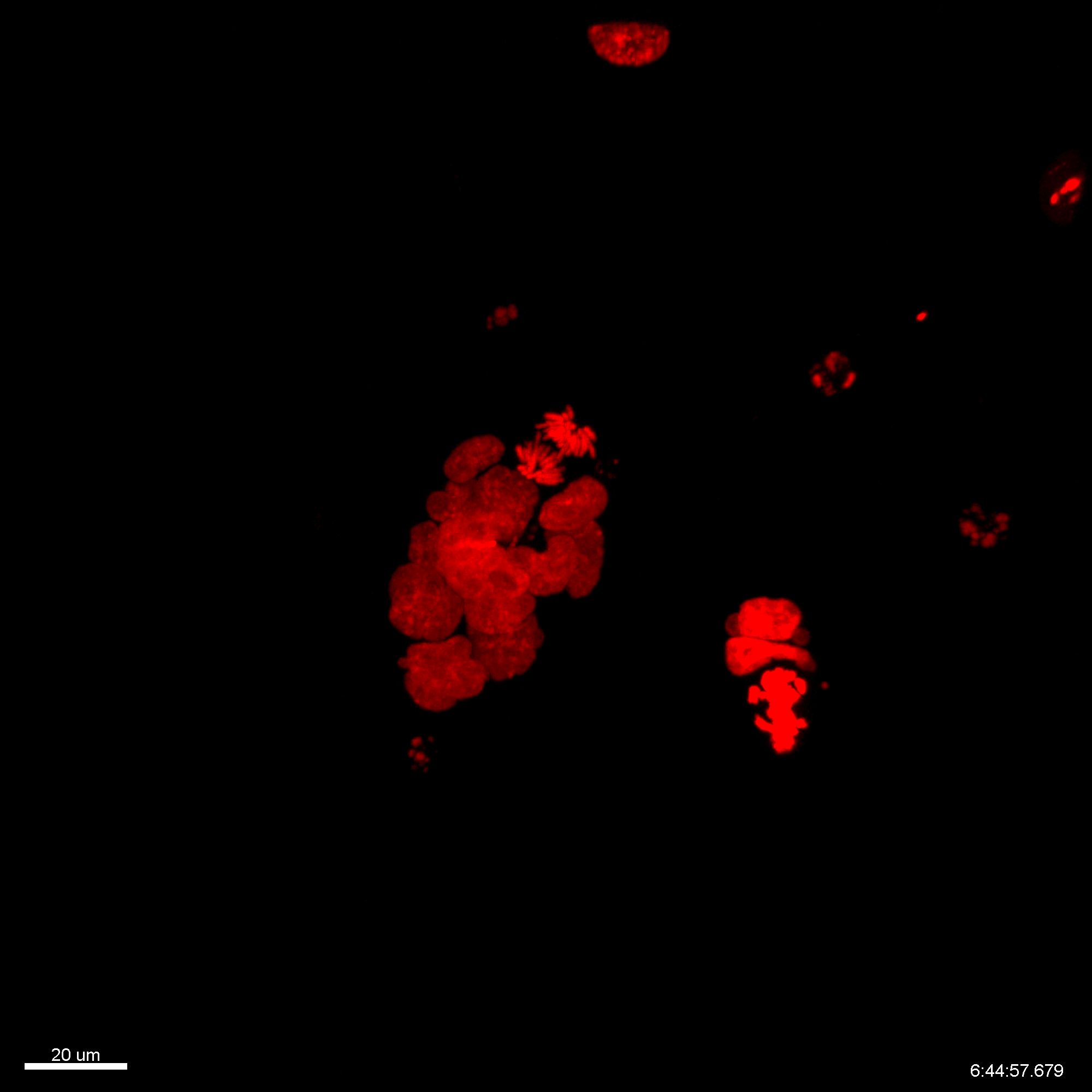

Supplement: Supplementary file 16 — Source data Fig. 2 [file 44320_2026_188_MOESM16_ESM.zip › Figure 2/2B/Live cell imaging WT 12 min.tif]

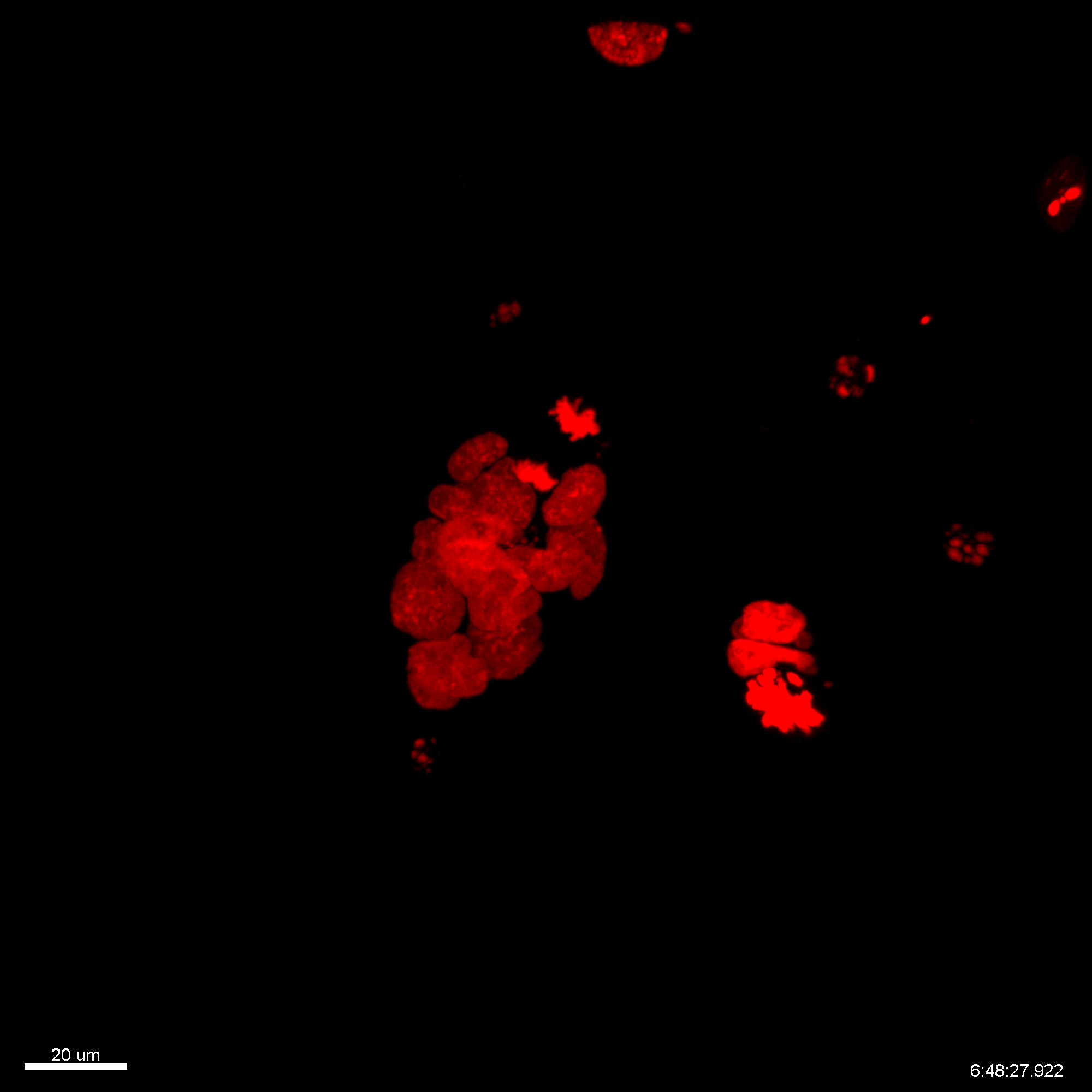

Supplement: Supplementary file 16 — Source data Fig. 2 [file 44320_2026_188_MOESM16_ESM.zip › Figure 2/2B/Live cell imaging WT 15 min.tif]

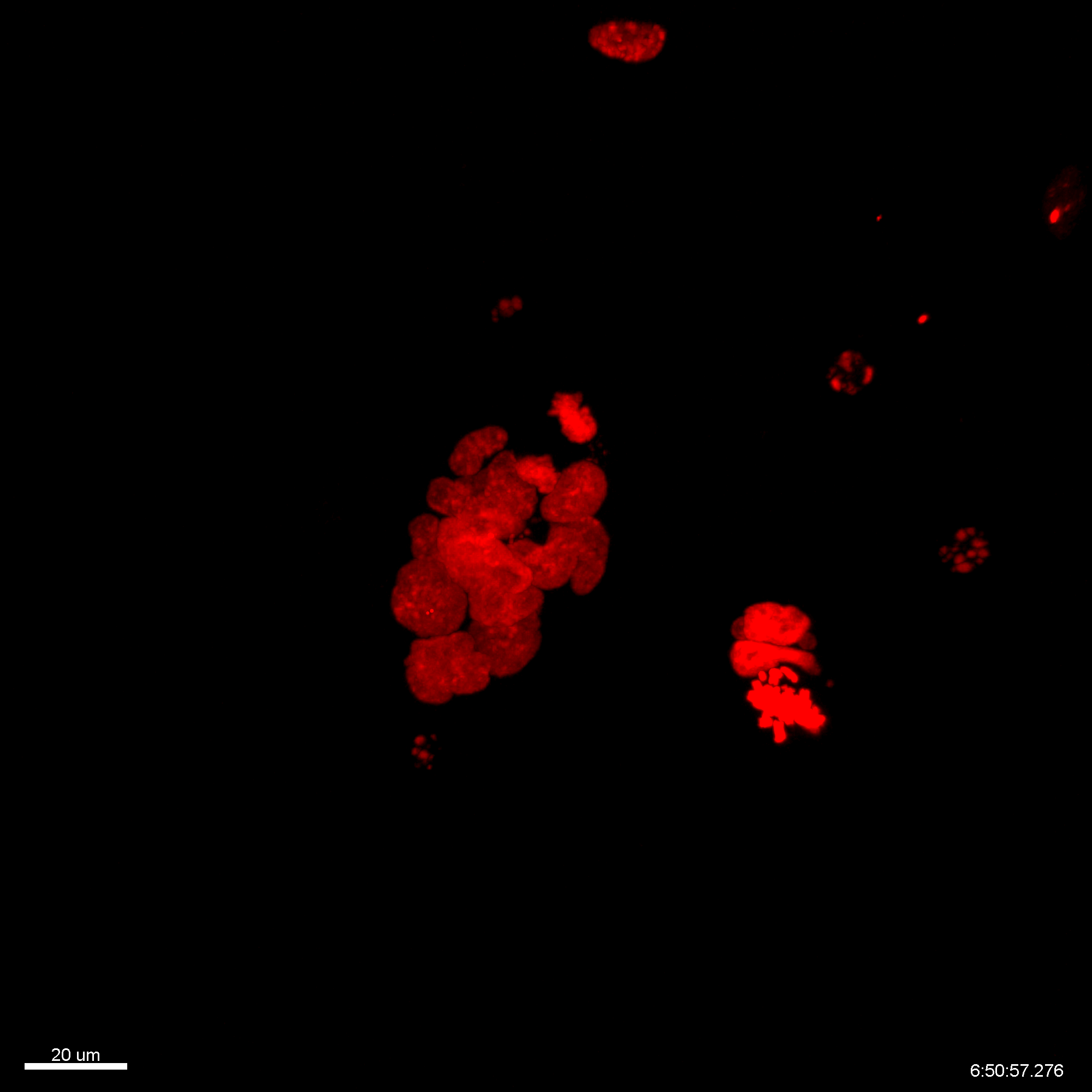

Supplement: Supplementary file 16 — Source data Fig. 2 [file 44320_2026_188_MOESM16_ESM.zip › Figure 2/2B/Live cell imaging WT 18 min.tif]

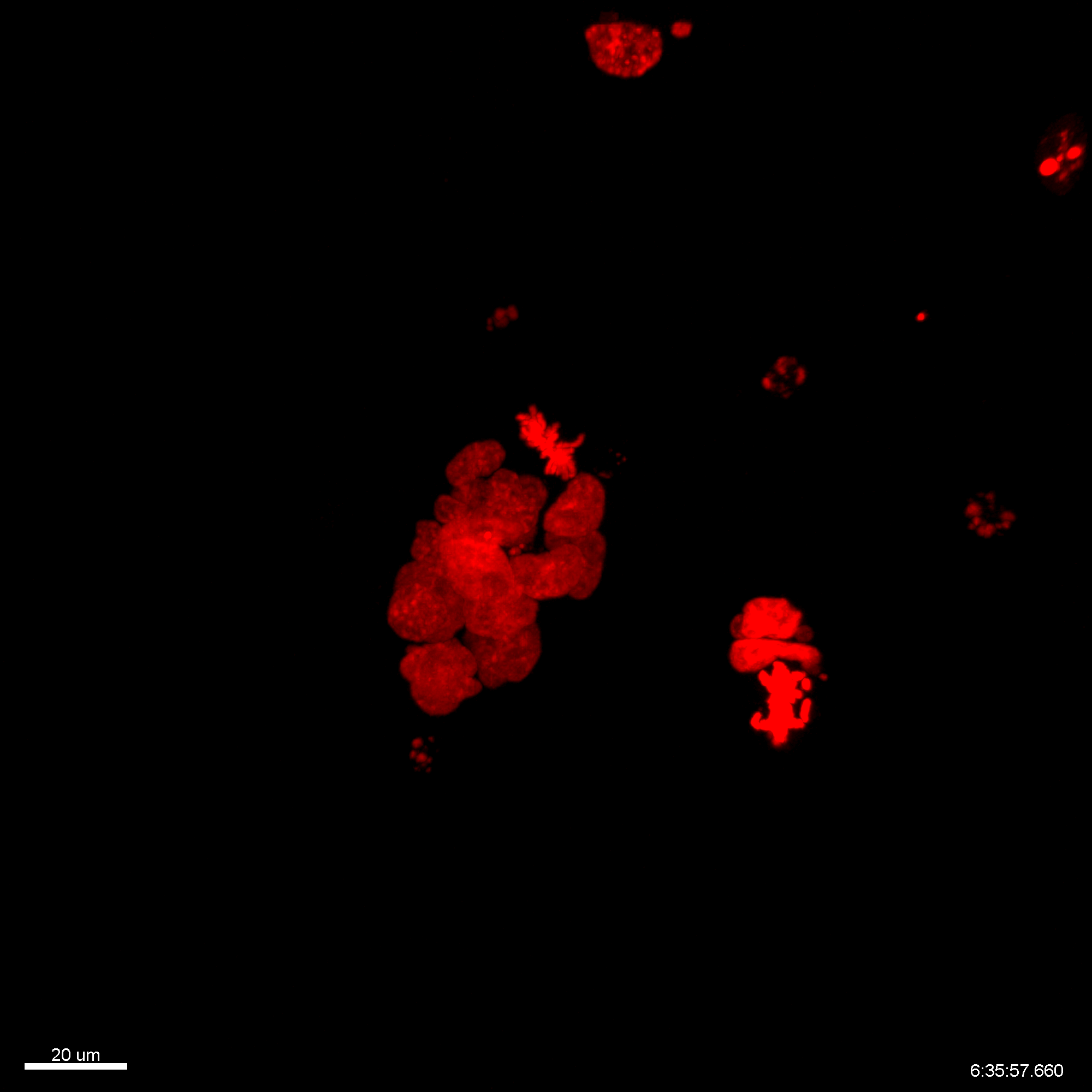

Supplement: Supplementary file 16 — Source data Fig. 2 [file 44320_2026_188_MOESM16_ESM.zip › Figure 2/2B/Live cell imaging WT 3 min.tif]

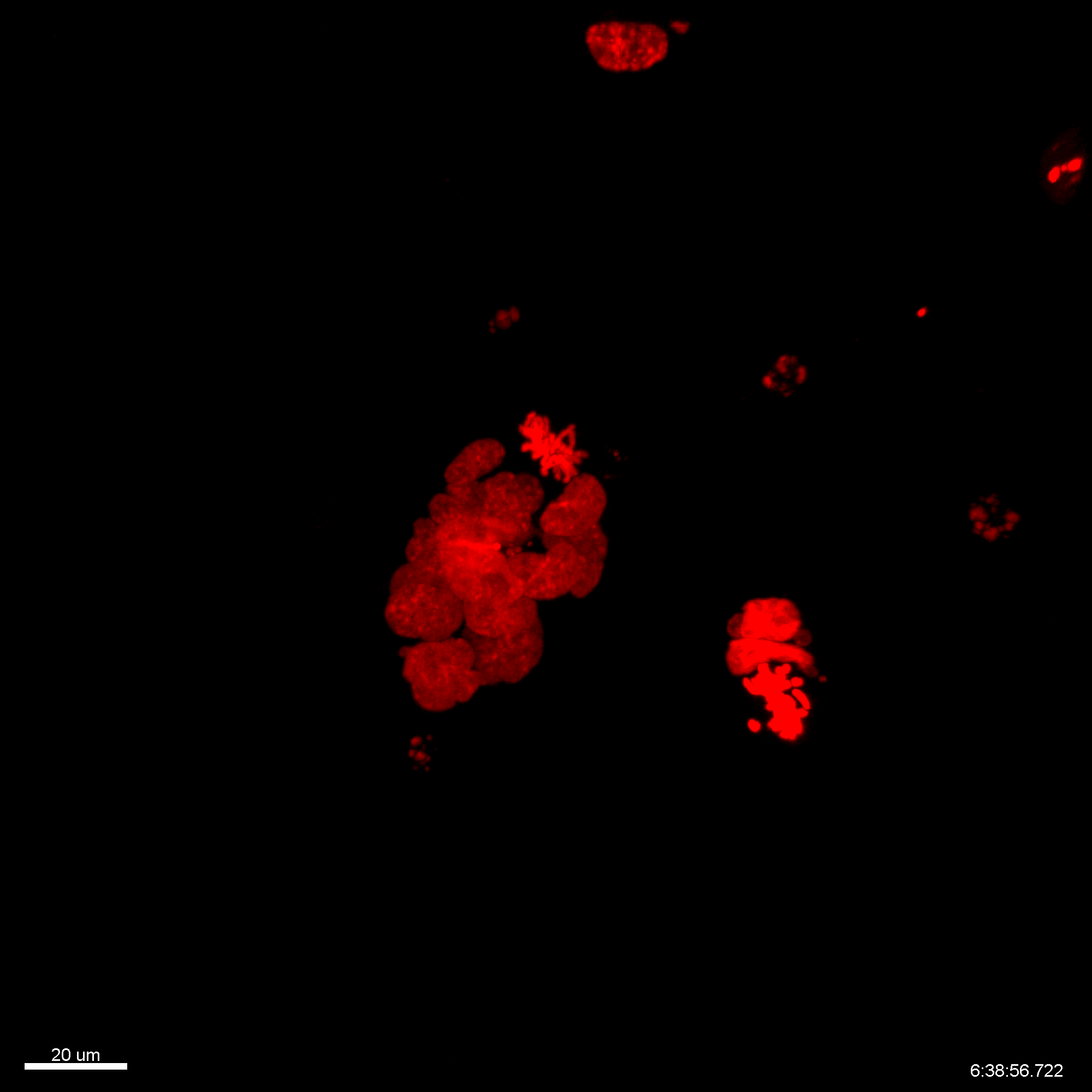

Supplement: Supplementary file 16 — Source data Fig. 2 [file 44320_2026_188_MOESM16_ESM.zip › Figure 2/2B/Live cell imaging WT 6 min.tif]

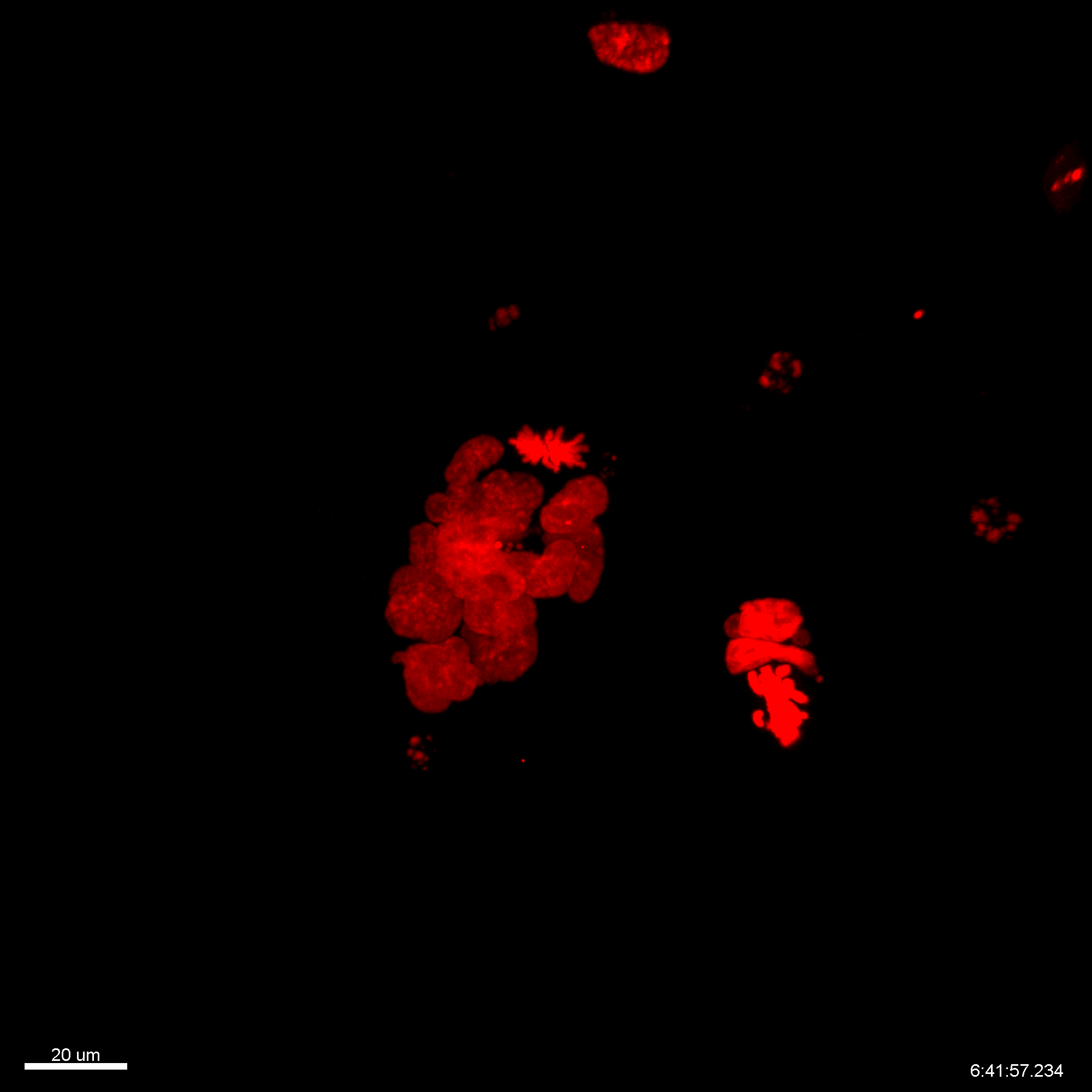

Supplement: Supplementary file 16 — Source data Fig. 2 [file 44320_2026_188_MOESM16_ESM.zip › Figure 2/2B/Live cell imaging WT 9 min.tif]

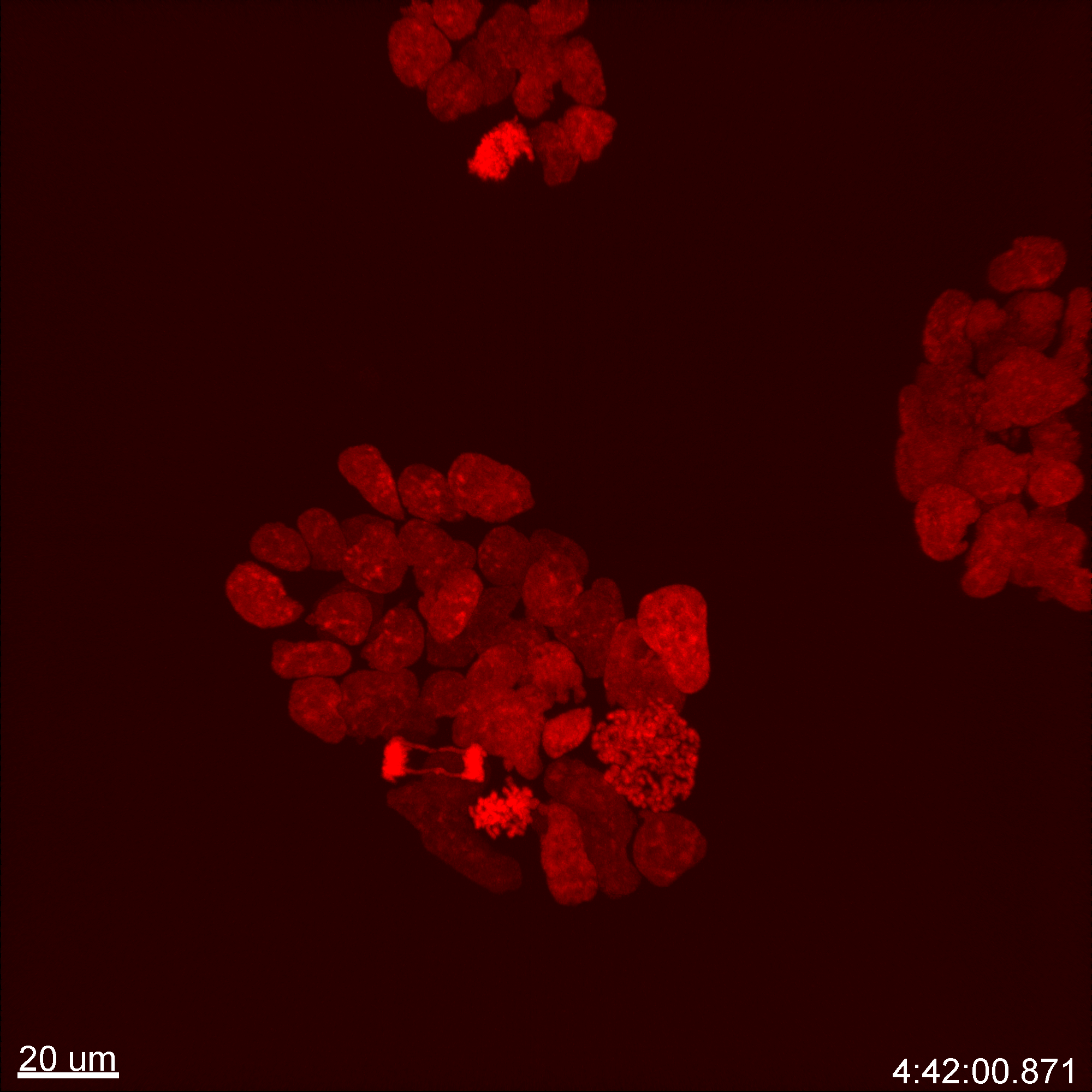

Supplement: Supplementary file 16 — Source data Fig. 2 [file 44320_2026_188_MOESM16_ESM.zip › Figure 2/2C/Live-cell imaging Chr2+3.tif]

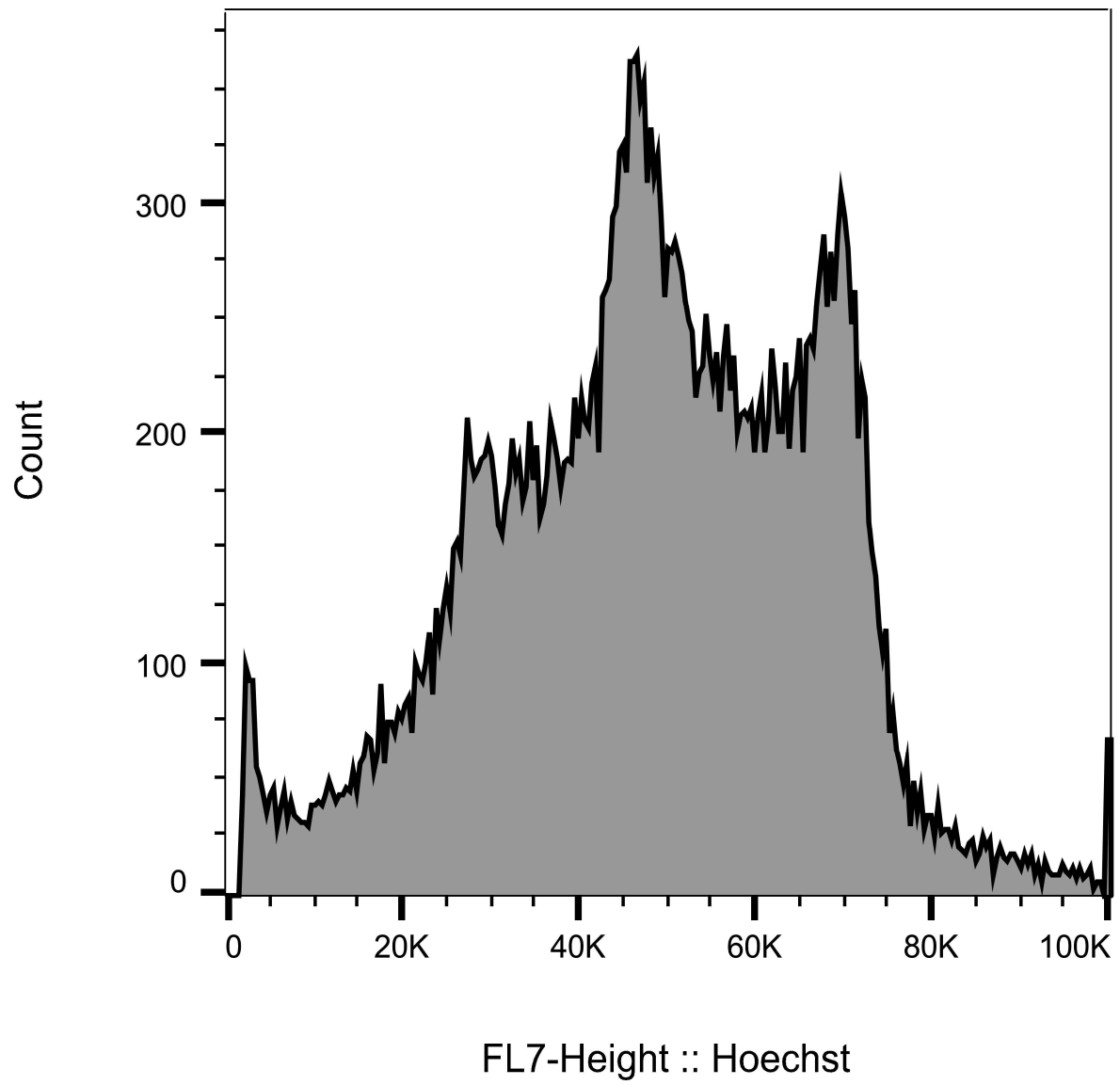

Supplement: Supplementary file 16 — Source data Fig. 2 [file 44320_2026_188_MOESM16_ESM.zip › Figure 2/2G/FACS Chr2+1 ESCs Day0.pdf]

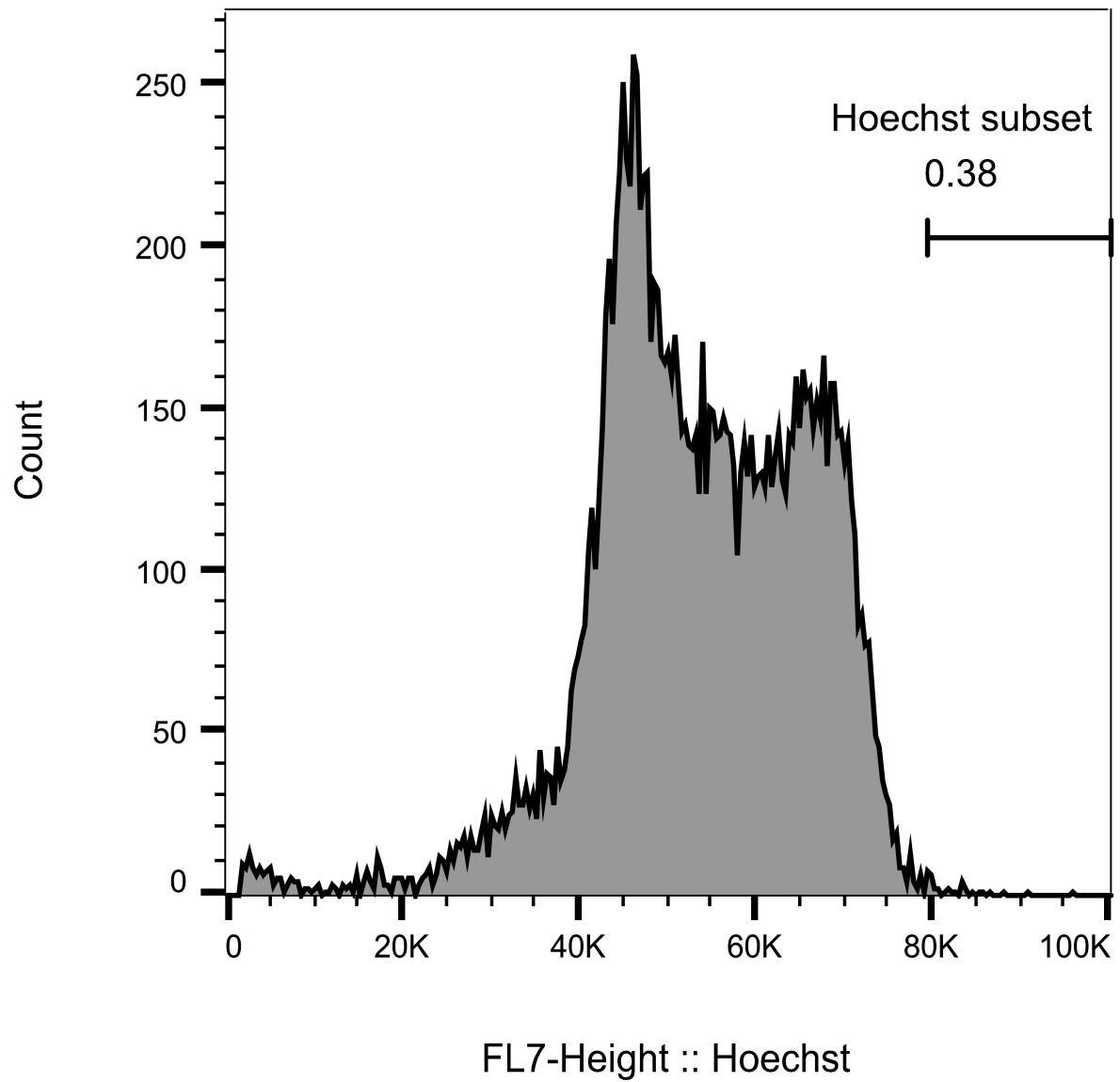

Supplement: Supplementary file 16 — Source data Fig. 2 [file 44320_2026_188_MOESM16_ESM.zip › Figure 2/2G/FACS Chr2+1 ESCs Day26.pdf]

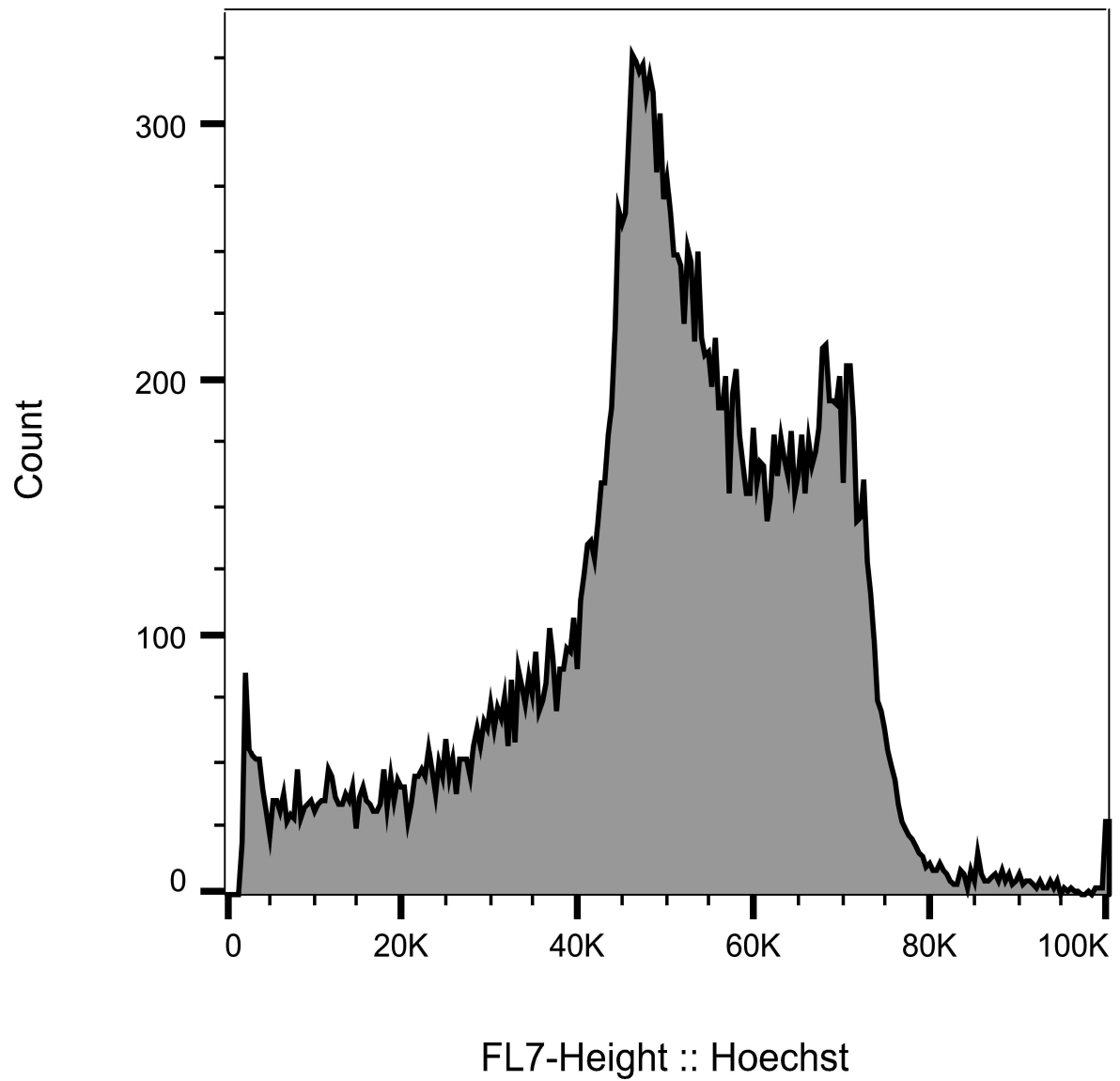

Supplement: Supplementary file 16 — Source data Fig. 2 [file 44320_2026_188_MOESM16_ESM.zip › Figure 2/2G/FACS Chr2+3 ESCs Day0.pdf]

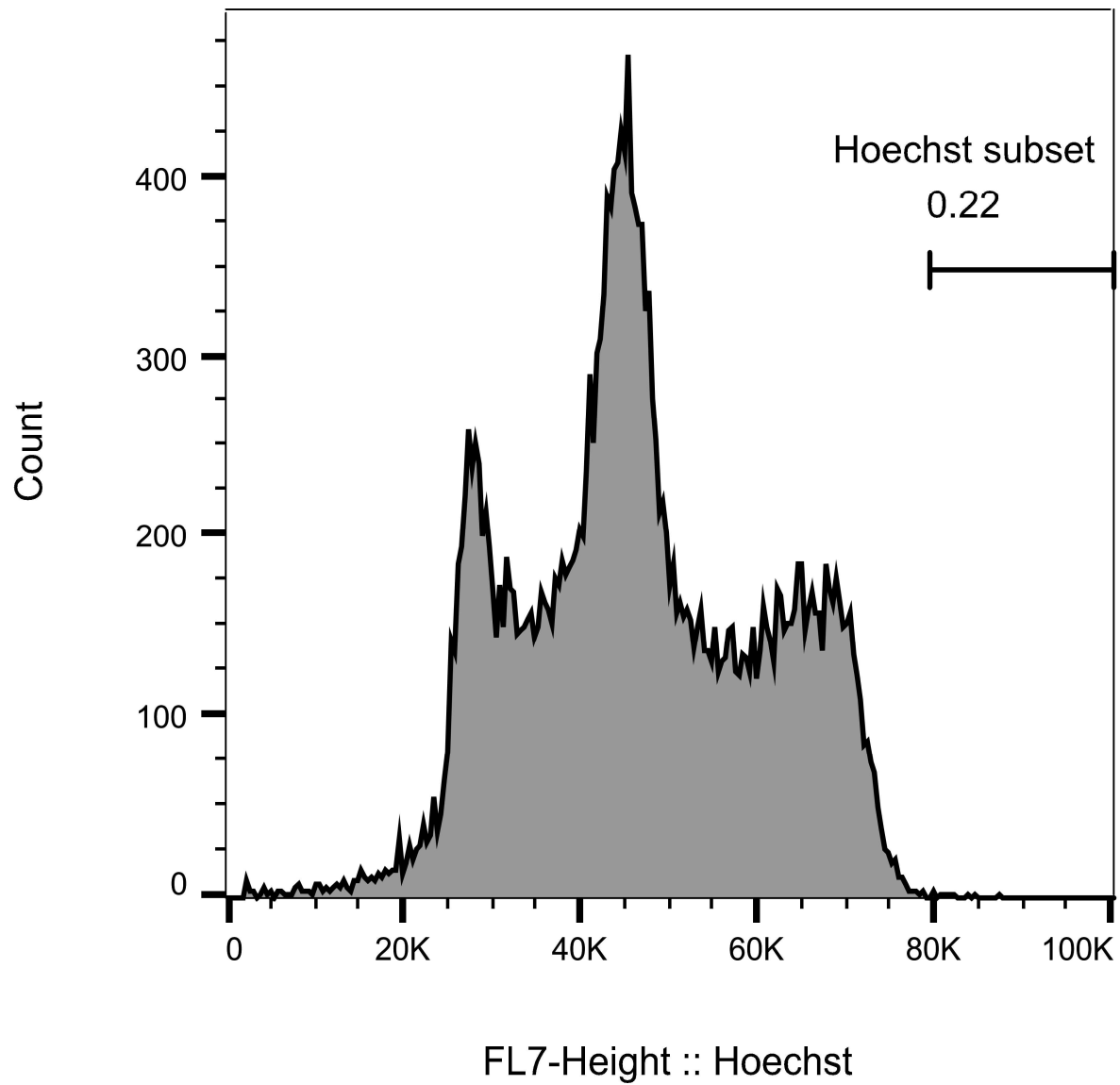

Supplement: Supplementary file 16 — Source data Fig. 2 [file 44320_2026_188_MOESM16_ESM.zip › Figure 2/2G/FACS Chr2+3 ESCs Day26.pdf]

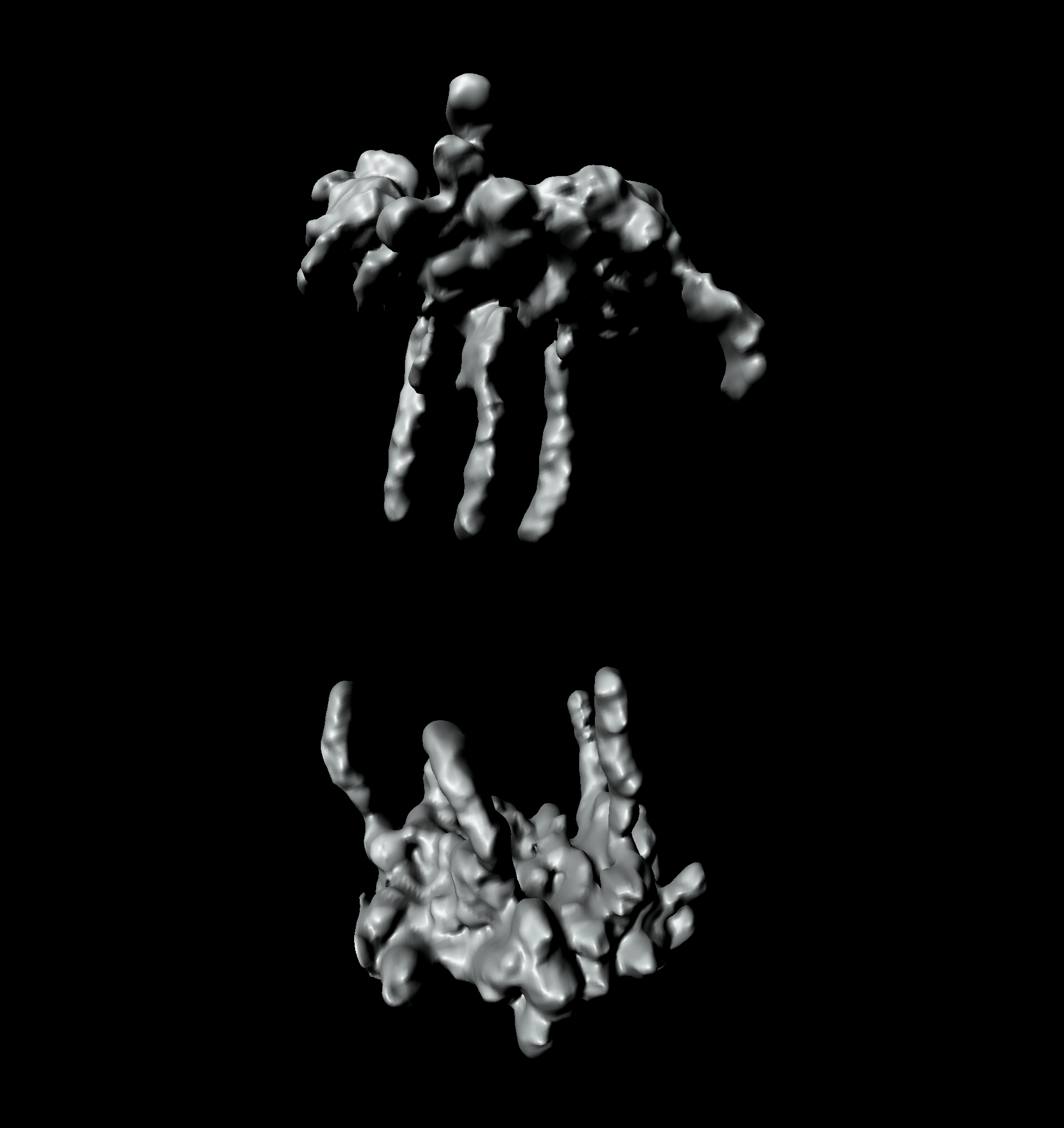

Supplement: Supplementary file 16 — Source data Fig. 2 [file 44320_2026_188_MOESM16_ESM.zip › Figure 2/2G/Live cell imaging 3D Chr2+1 ESCs.tif]

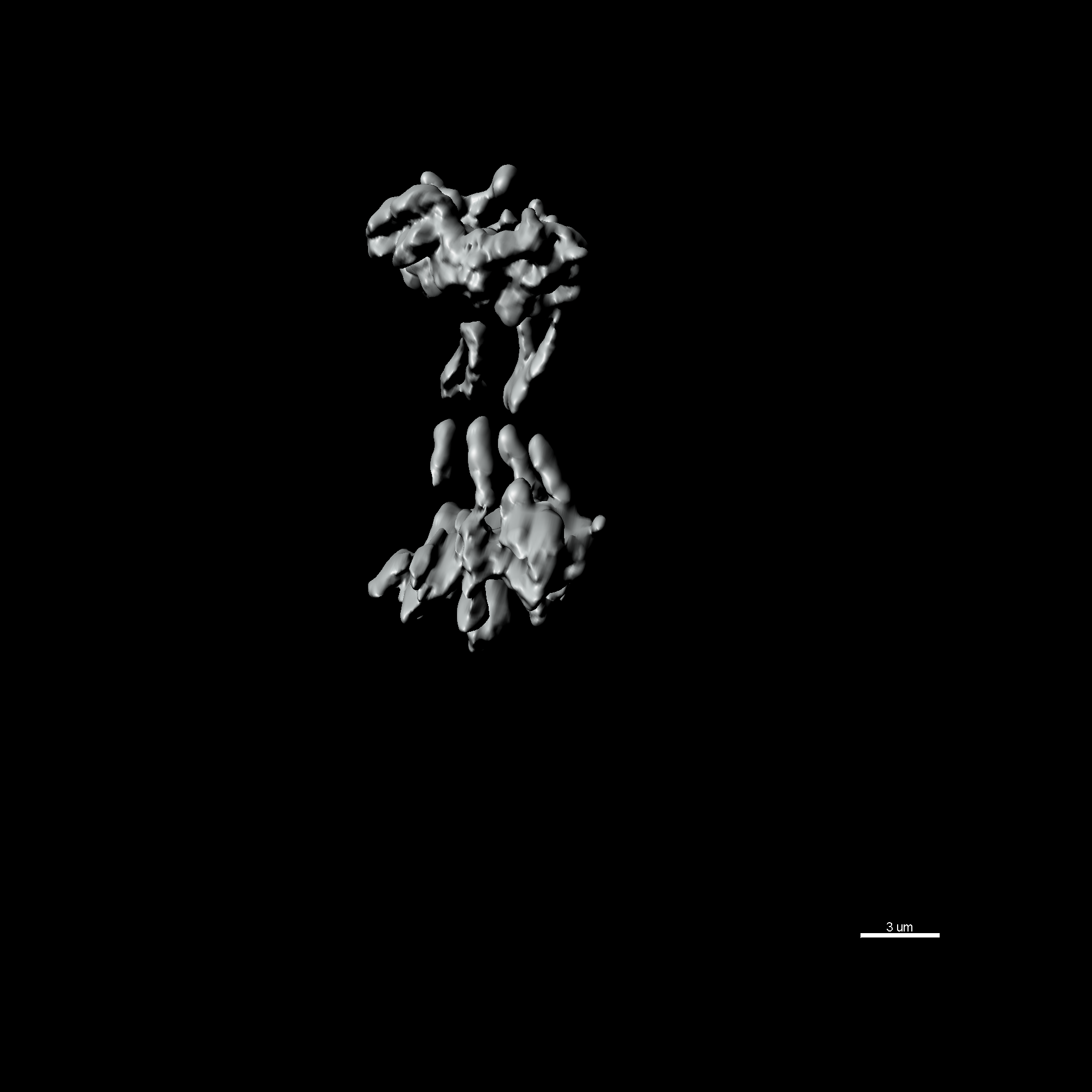

Supplement: Supplementary file 16 — Source data Fig. 2 [file 44320_2026_188_MOESM16_ESM.zip › Figure 2/2G/Live cell imaging 3D Chr2+3 ESCs.tif]

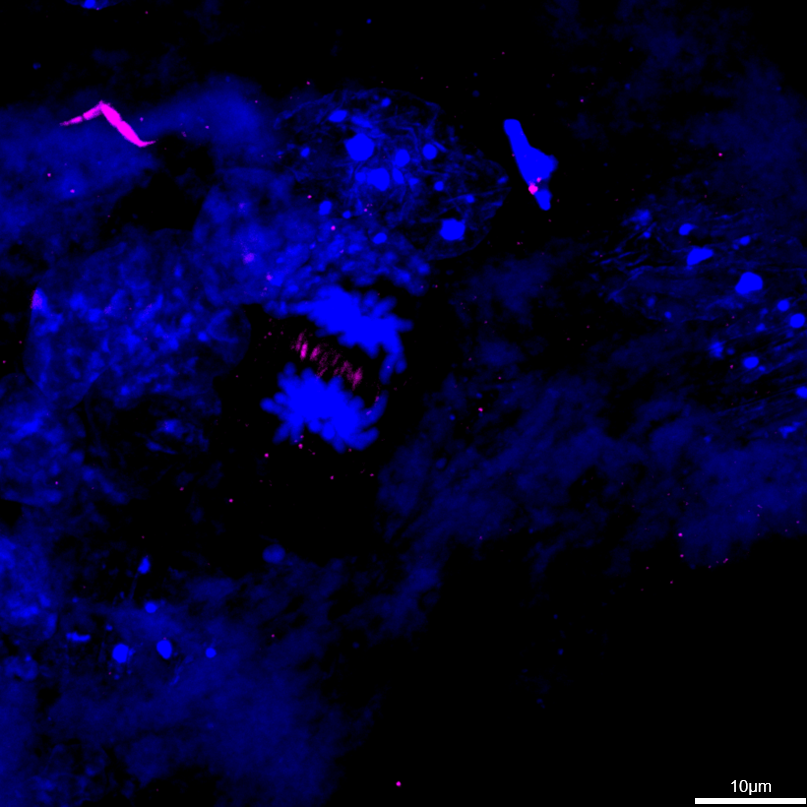

Supplement: Supplementary file 17 — Source data Fig. 3 [file 44320_2026_188_MOESM17_ESM.zip › Figure 3/3A/Confocal Chr1+2' DNA Aurora-1.tif]

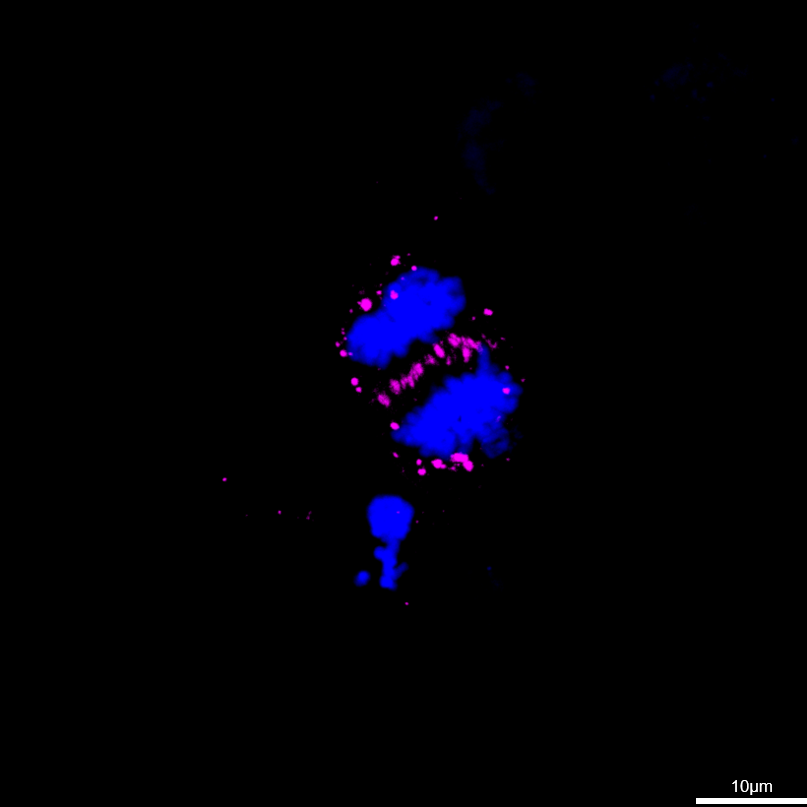

Supplement: Supplementary file 17 — Source data Fig. 3 [file 44320_2026_188_MOESM17_ESM.zip › Figure 3/3A/Confocal Chr1+2' DNA Aurora-2.tif]

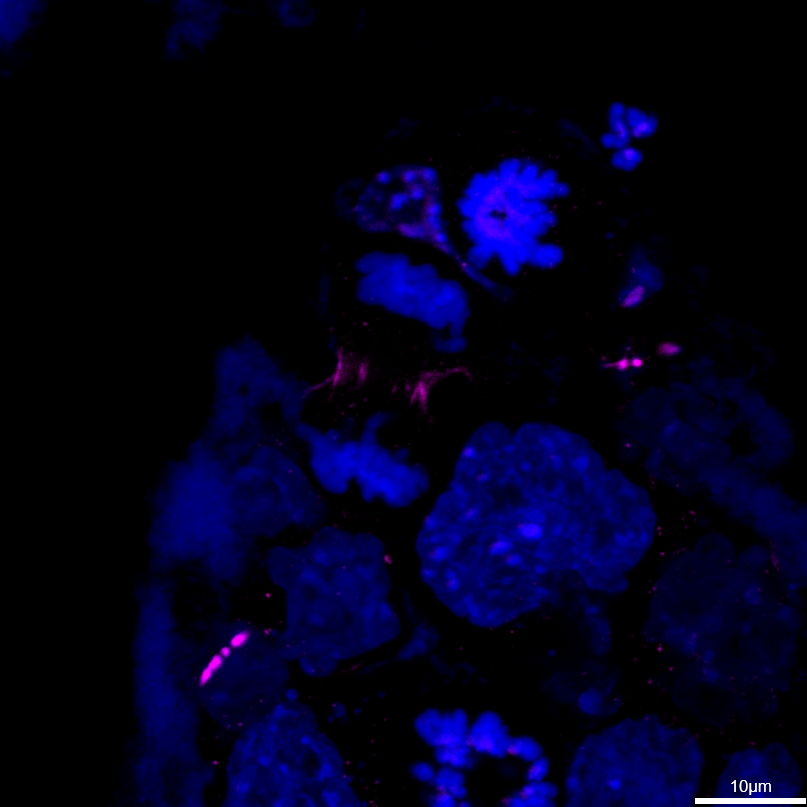

Supplement: Supplementary file 17 — Source data Fig. 3 [file 44320_2026_188_MOESM17_ESM.zip › Figure 3/3A/Confocal Chr1+2' DNA Aurora-3.tif]

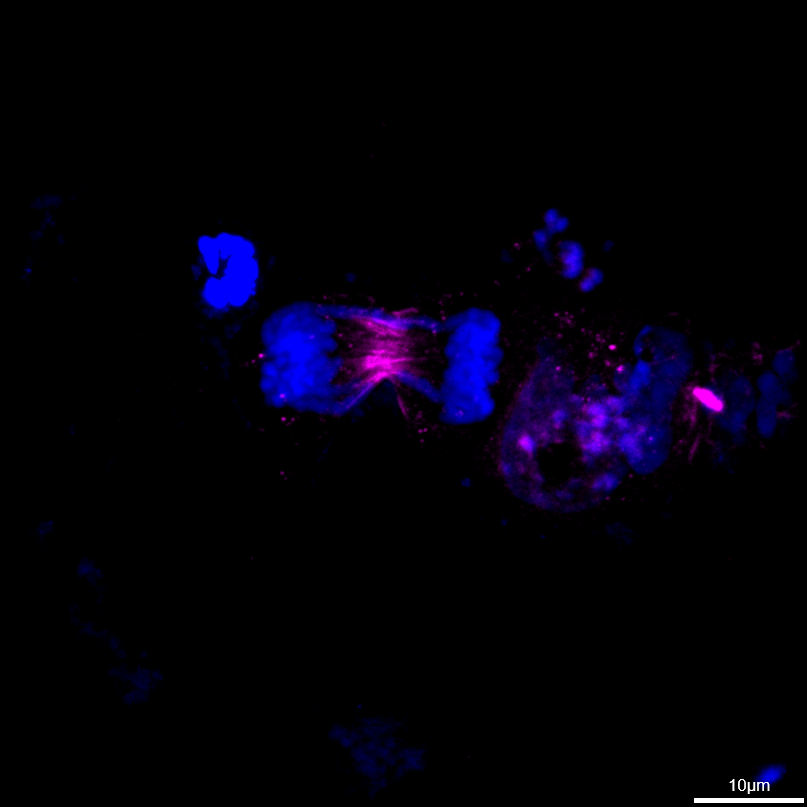

Supplement: Supplementary file 17 — Source data Fig. 3 [file 44320_2026_188_MOESM17_ESM.zip › Figure 3/3A/Confocal Chr2+1 DNA Aurora B-1.tif]

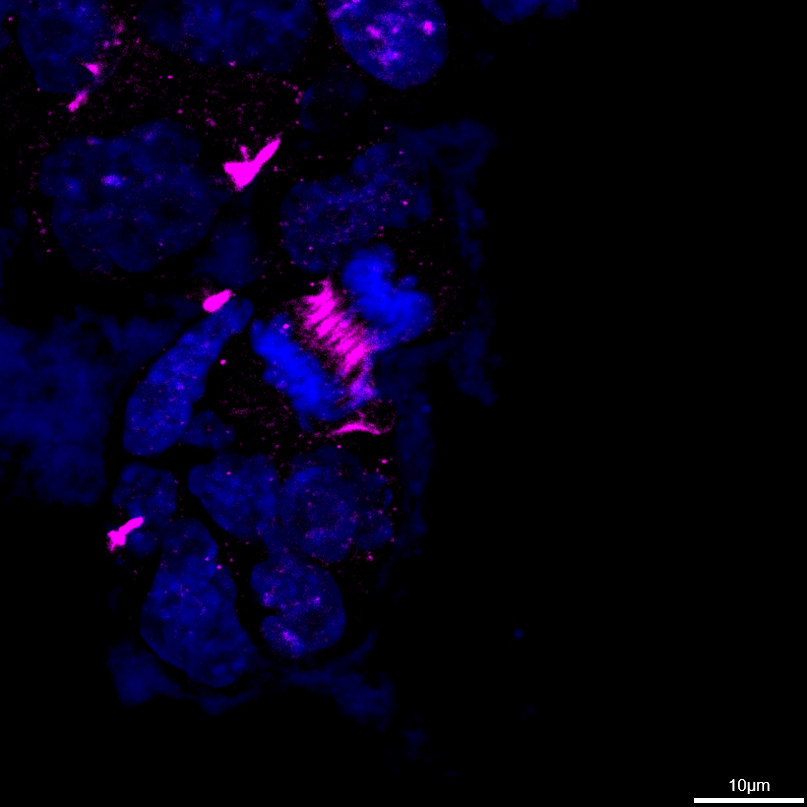

Supplement: Supplementary file 17 — Source data Fig. 3 [file 44320_2026_188_MOESM17_ESM.zip › Figure 3/3A/Confocal Chr2+1 DNA Aurora B-2.tif]

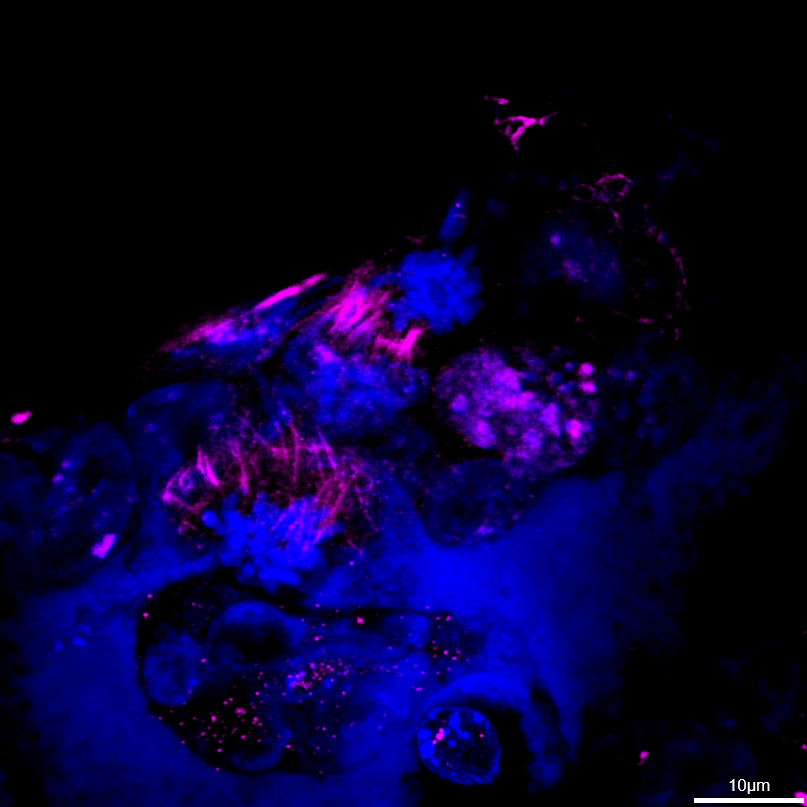

Supplement: Supplementary file 17 — Source data Fig. 3 [file 44320_2026_188_MOESM17_ESM.zip › Figure 3/3A/Confocal Chr2+1 DNA Aurora B-3.tif]

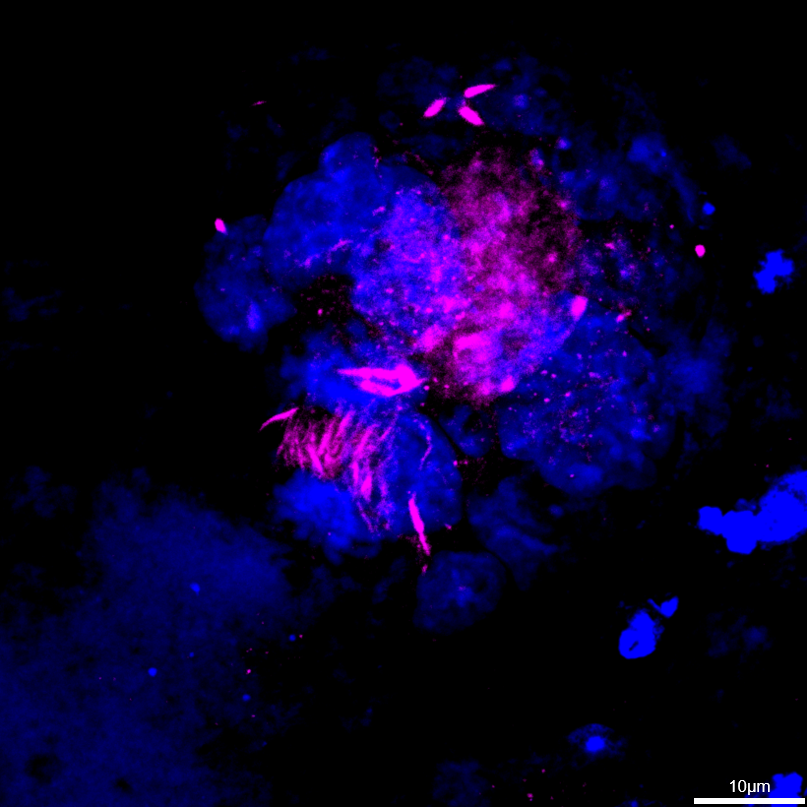

Supplement: Supplementary file 17 — Source data Fig. 3 [file 44320_2026_188_MOESM17_ESM.zip › Figure 3/3A/Confocal Chr2+3 DNA Aurora B-1.tif]

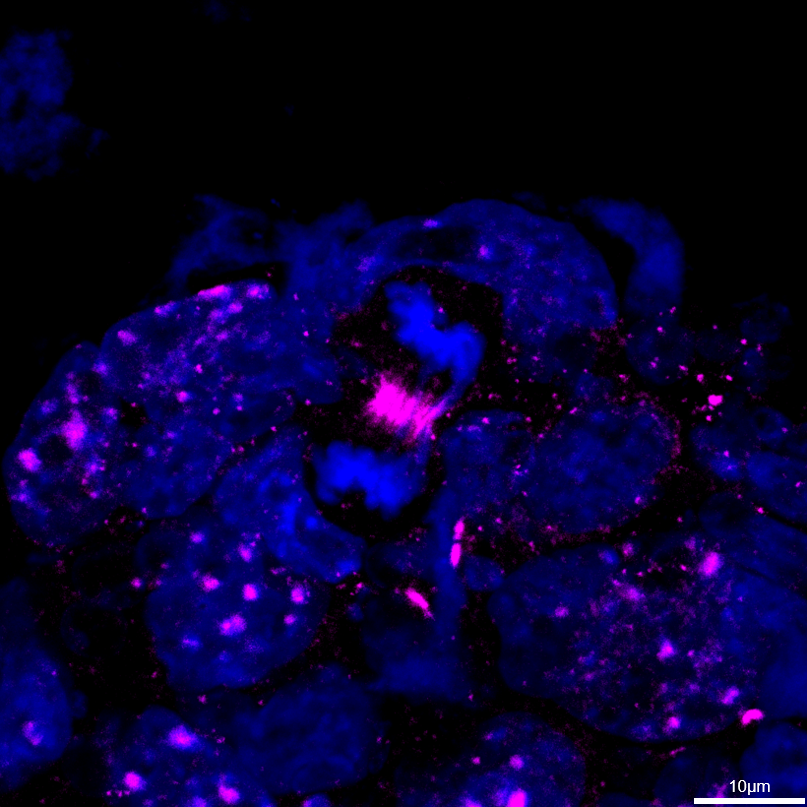

Supplement: Supplementary file 17 — Source data Fig. 3 [file 44320_2026_188_MOESM17_ESM.zip › Figure 3/3A/Confocal Chr2+3 DNA Aurora B-2.tif]

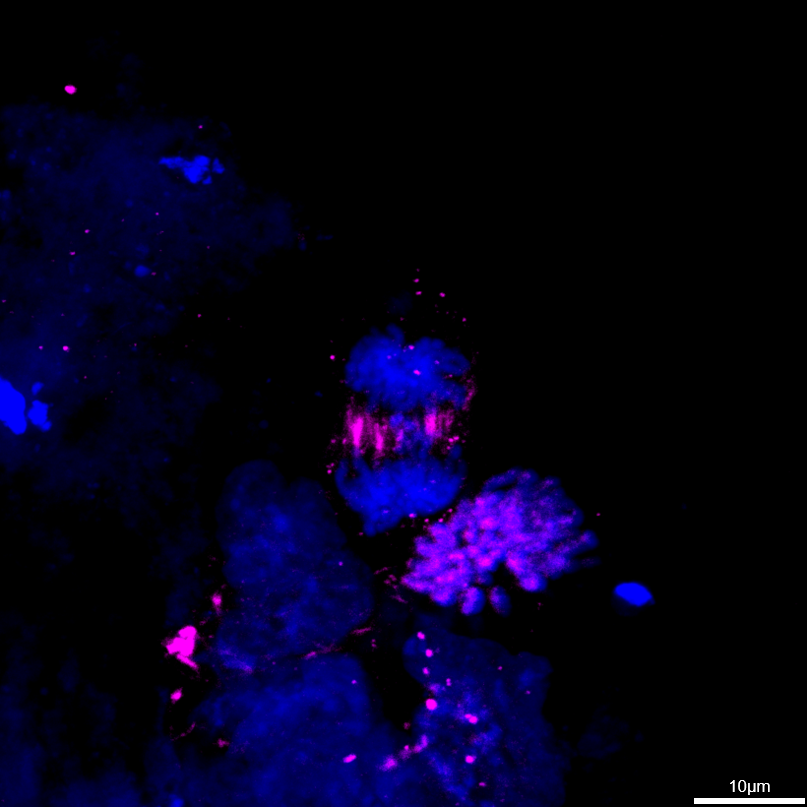

Supplement: Supplementary file 17 — Source data Fig. 3 [file 44320_2026_188_MOESM17_ESM.zip › Figure 3/3A/Confocal Chr2+3 DNA Aurora B-3.tif]

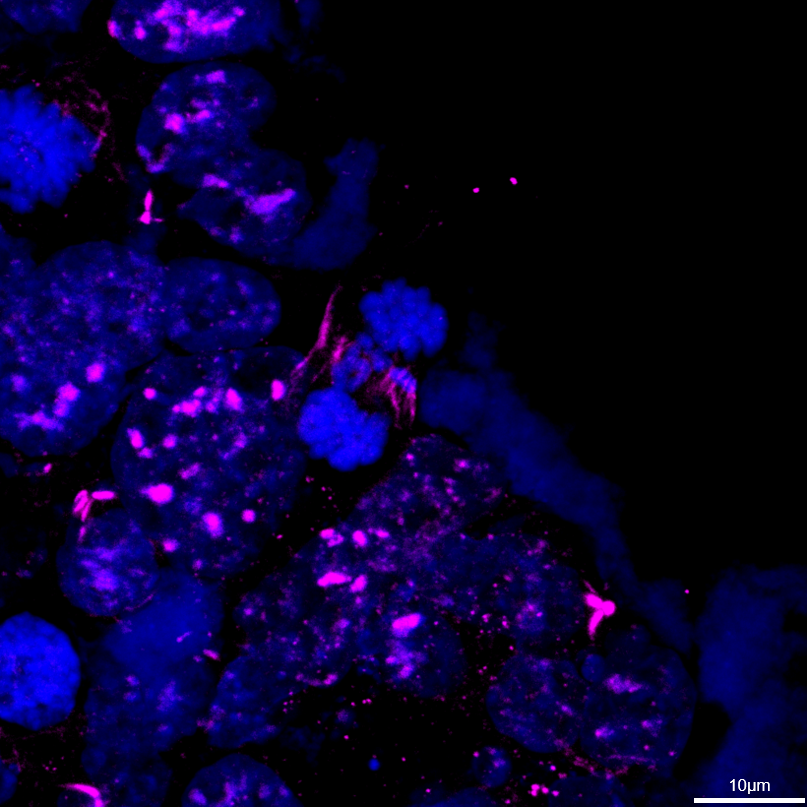

Supplement: Supplementary file 17 — Source data Fig. 3 [file 44320_2026_188_MOESM17_ESM.zip › Figure 3/3A/Confocal Chr4+5 DNA Aurora B-1.tif]

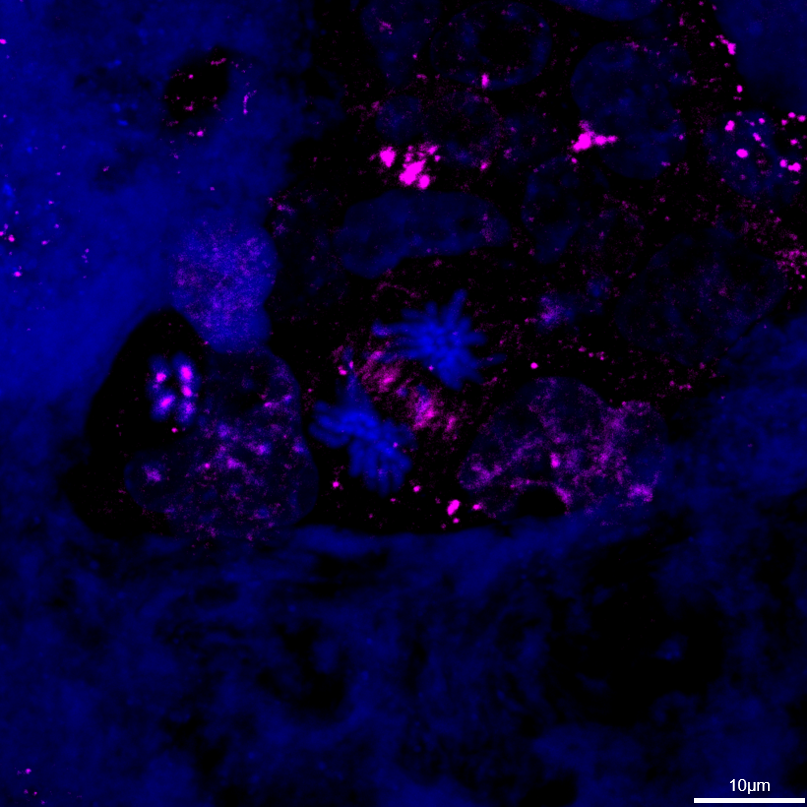

Supplement: Supplementary file 17 — Source data Fig. 3 [file 44320_2026_188_MOESM17_ESM.zip › Figure 3/3A/Confocal Chr4+5 DNA Aurora B-2.tif]

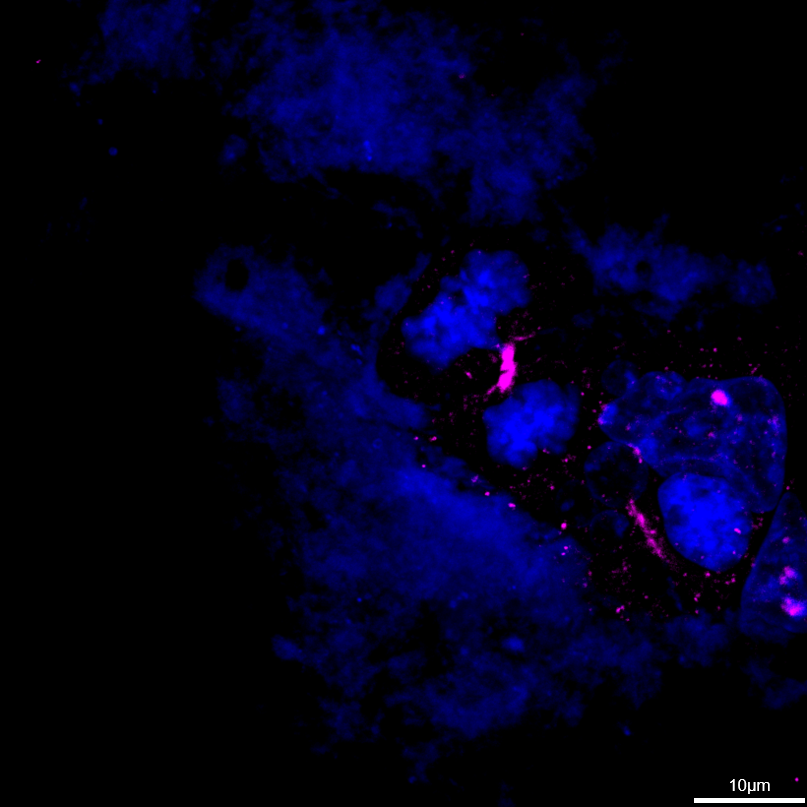

Supplement: Supplementary file 17 — Source data Fig. 3 [file 44320_2026_188_MOESM17_ESM.zip › Figure 3/3A/Confocal Chr4+5 DNA Aurora B-3.tif]

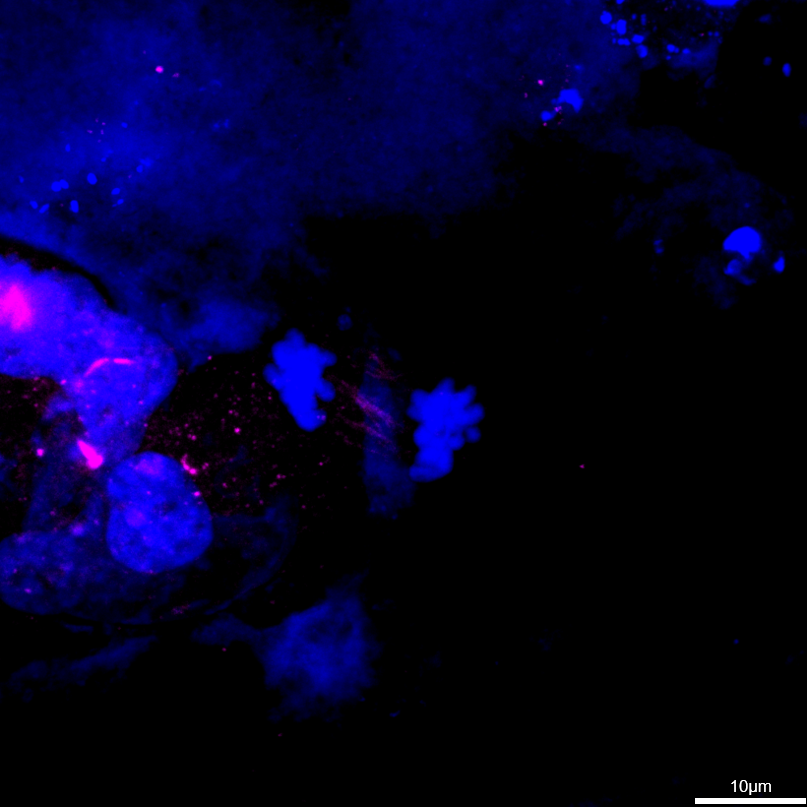

Supplement: Supplementary file 17 — Source data Fig. 3 [file 44320_2026_188_MOESM17_ESM.zip › Figure 3/3A/Confocal WT DNA Aurora B-1.tif]

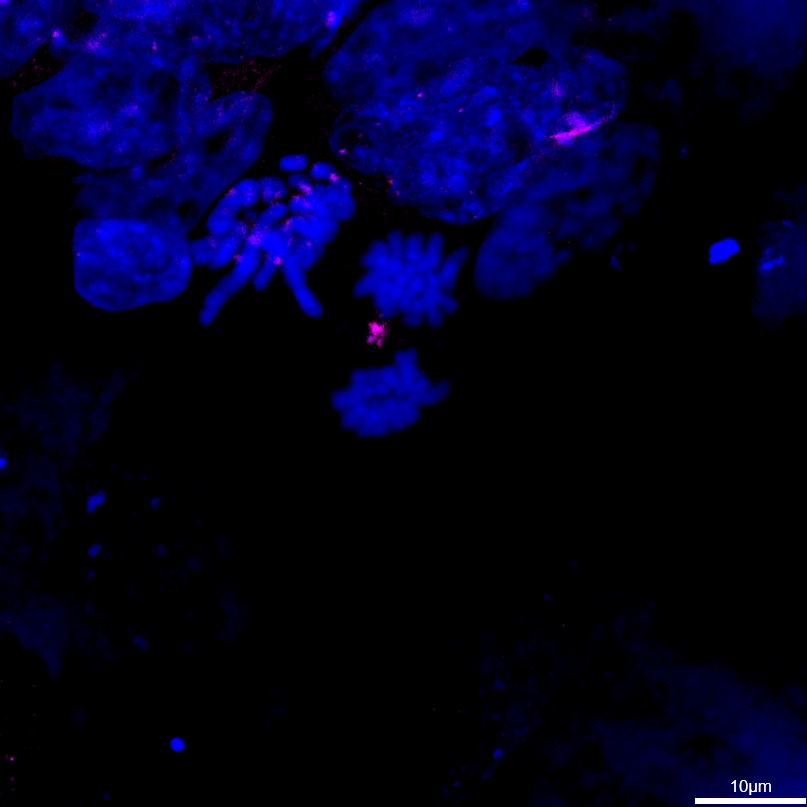

Supplement: Supplementary file 17 — Source data Fig. 3 [file 44320_2026_188_MOESM17_ESM.zip › Figure 3/3A/Confocal WT DNA Aurora B-2.tif]

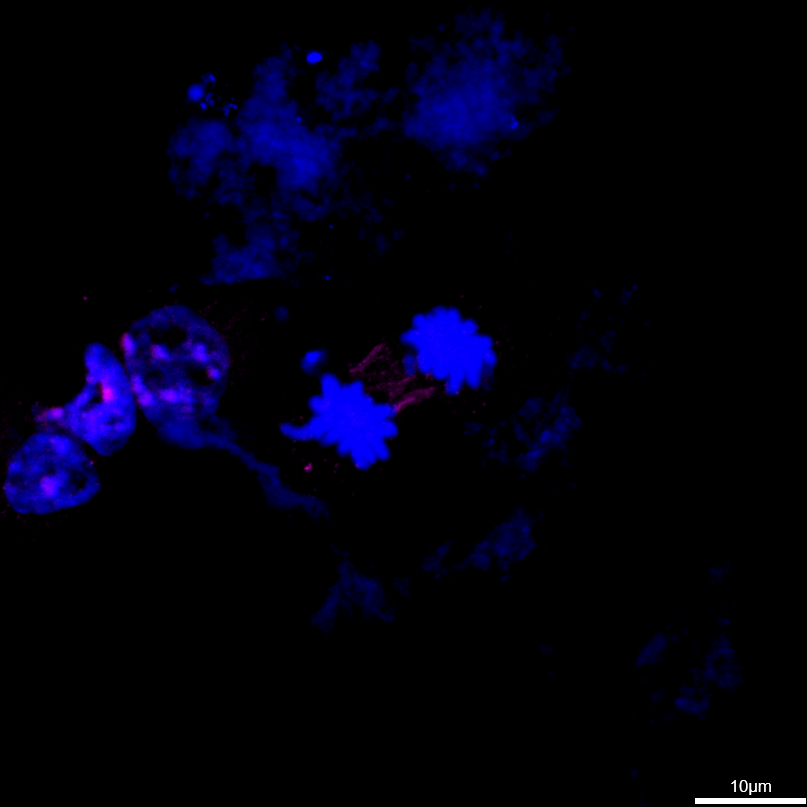

Supplement: Supplementary file 17 — Source data Fig. 3 [file 44320_2026_188_MOESM17_ESM.zip › Figure 3/3A/Confocal WT DNA Aurora B-3.tif]

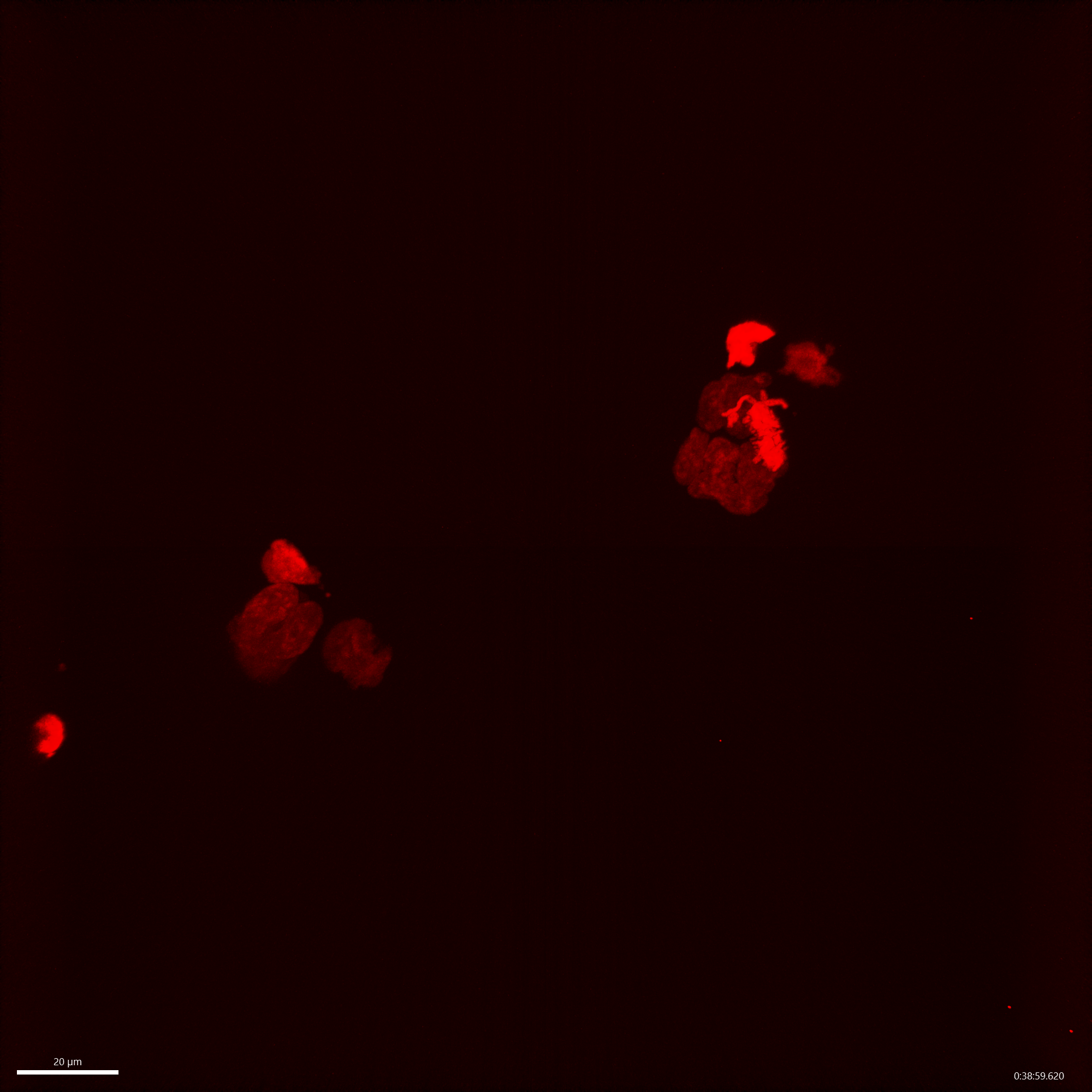

Supplement: Supplementary file 17 — Source data Fig. 3 [file 44320_2026_188_MOESM17_ESM.zip › Figure 3/3E/Live cell imaging Chr2+1 Aurora B-Inhibitor 0min.tif]

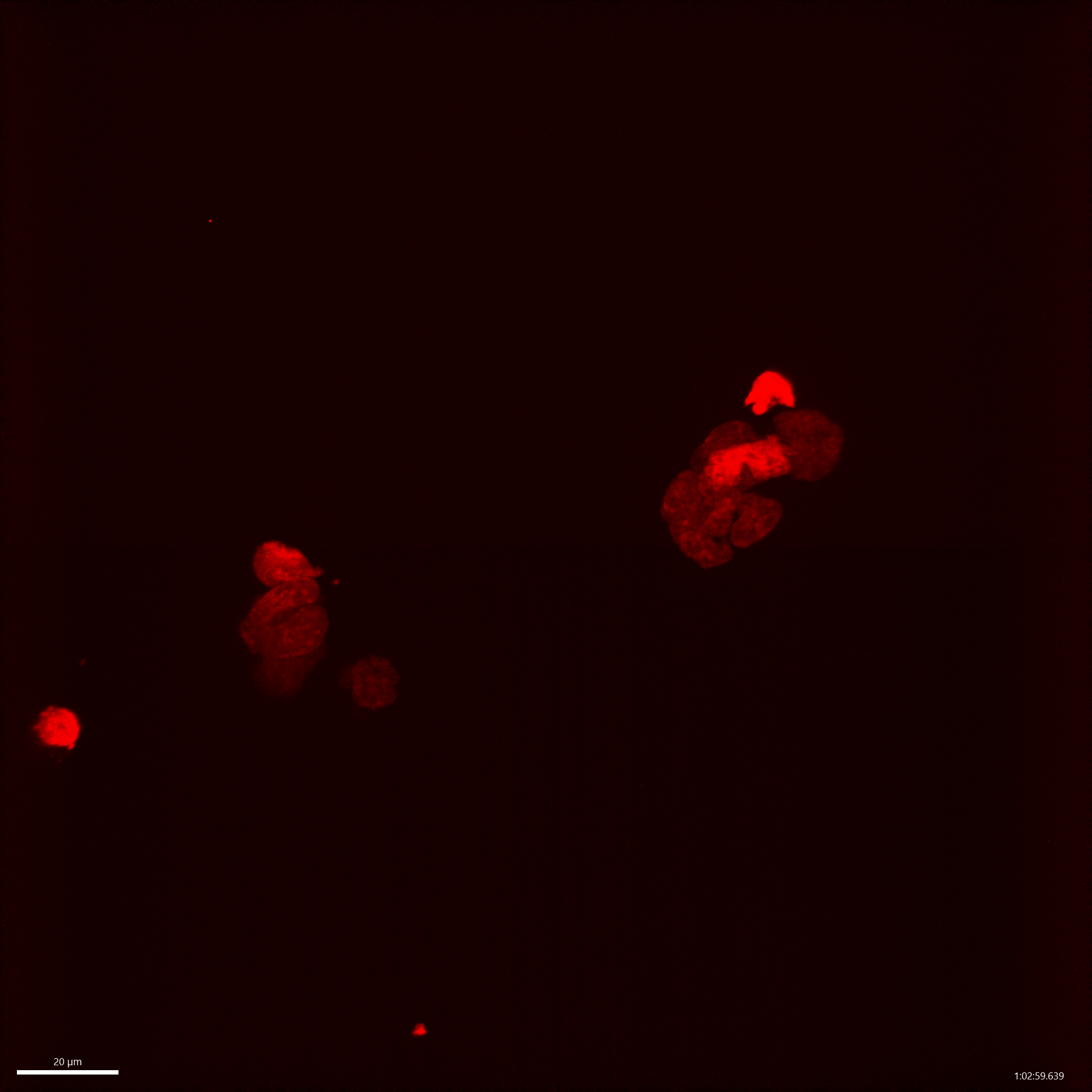

Supplement: Supplementary file 17 — Source data Fig. 3 [file 44320_2026_188_MOESM17_ESM.zip › Figure 3/3E/Live cell imaging Chr2+1 Aurora B-Inhibitor 15min.tif]

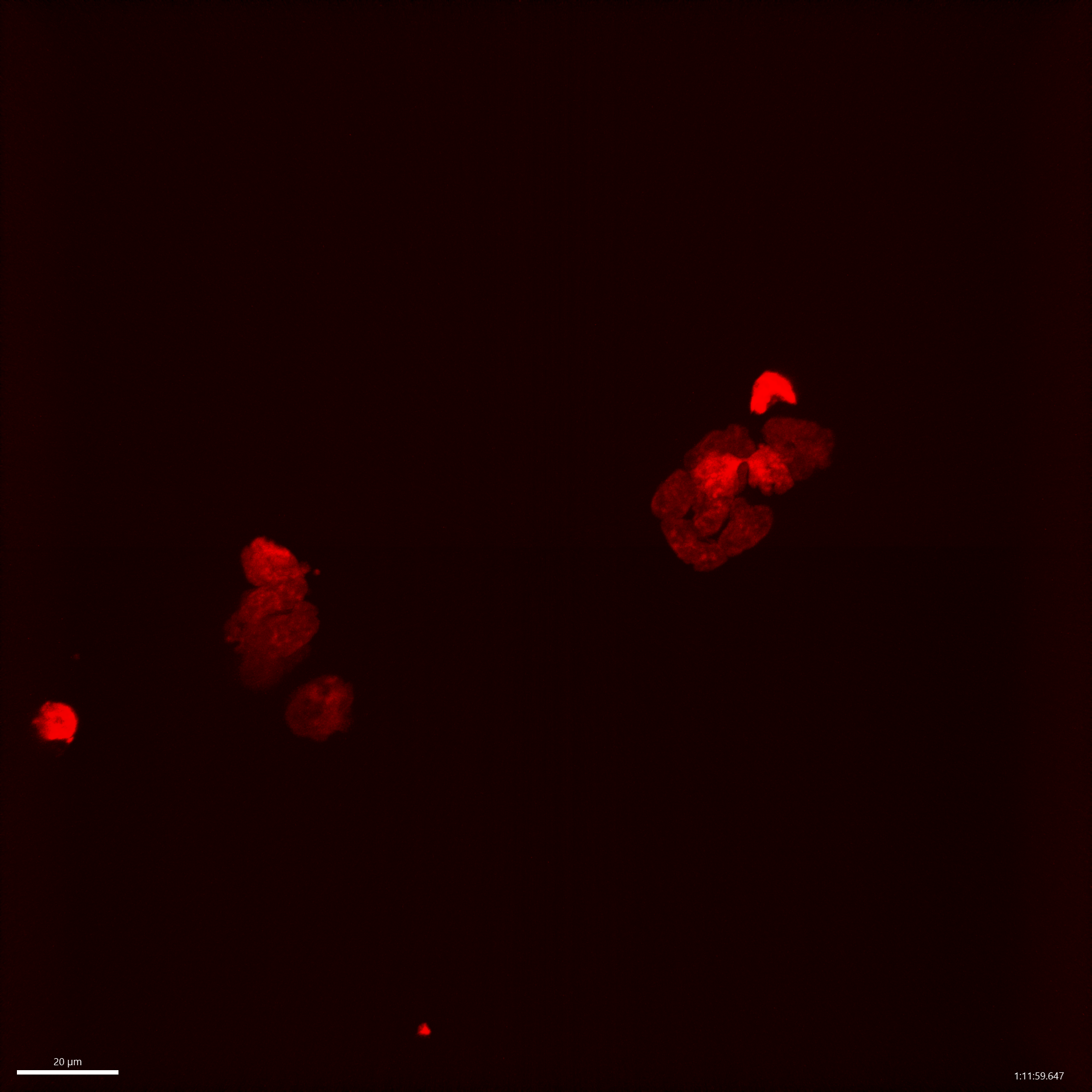

Supplement: Supplementary file 17 — Source data Fig. 3 [file 44320_2026_188_MOESM17_ESM.zip › Figure 3/3E/Live cell imaging Chr2+1 Aurora B-Inhibitor 24min.tif]

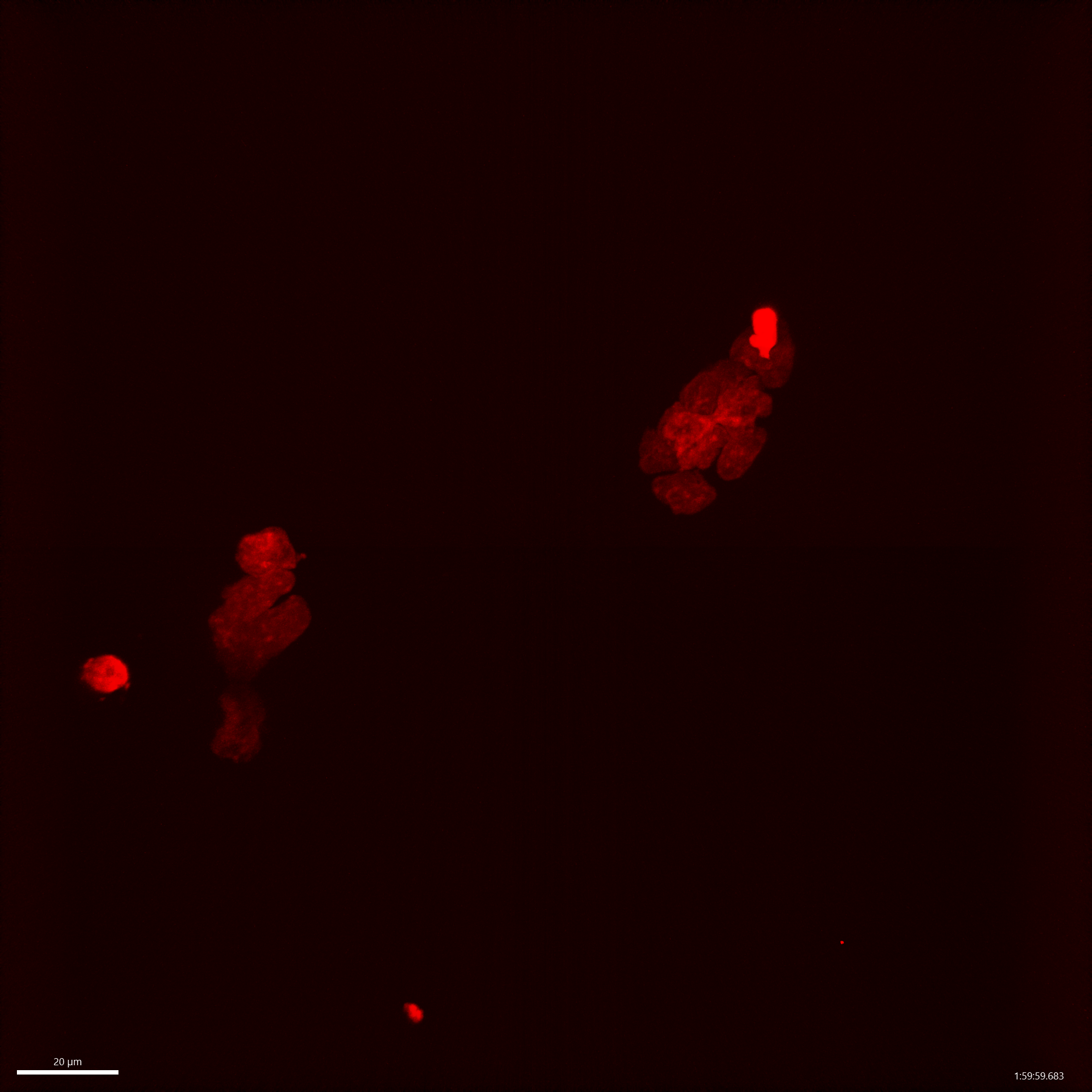

Supplement: Supplementary file 17 — Source data Fig. 3 [file 44320_2026_188_MOESM17_ESM.zip › Figure 3/3E/Live cell imaging Chr2+1 Aurora B-Inhibitor 48min.tif]

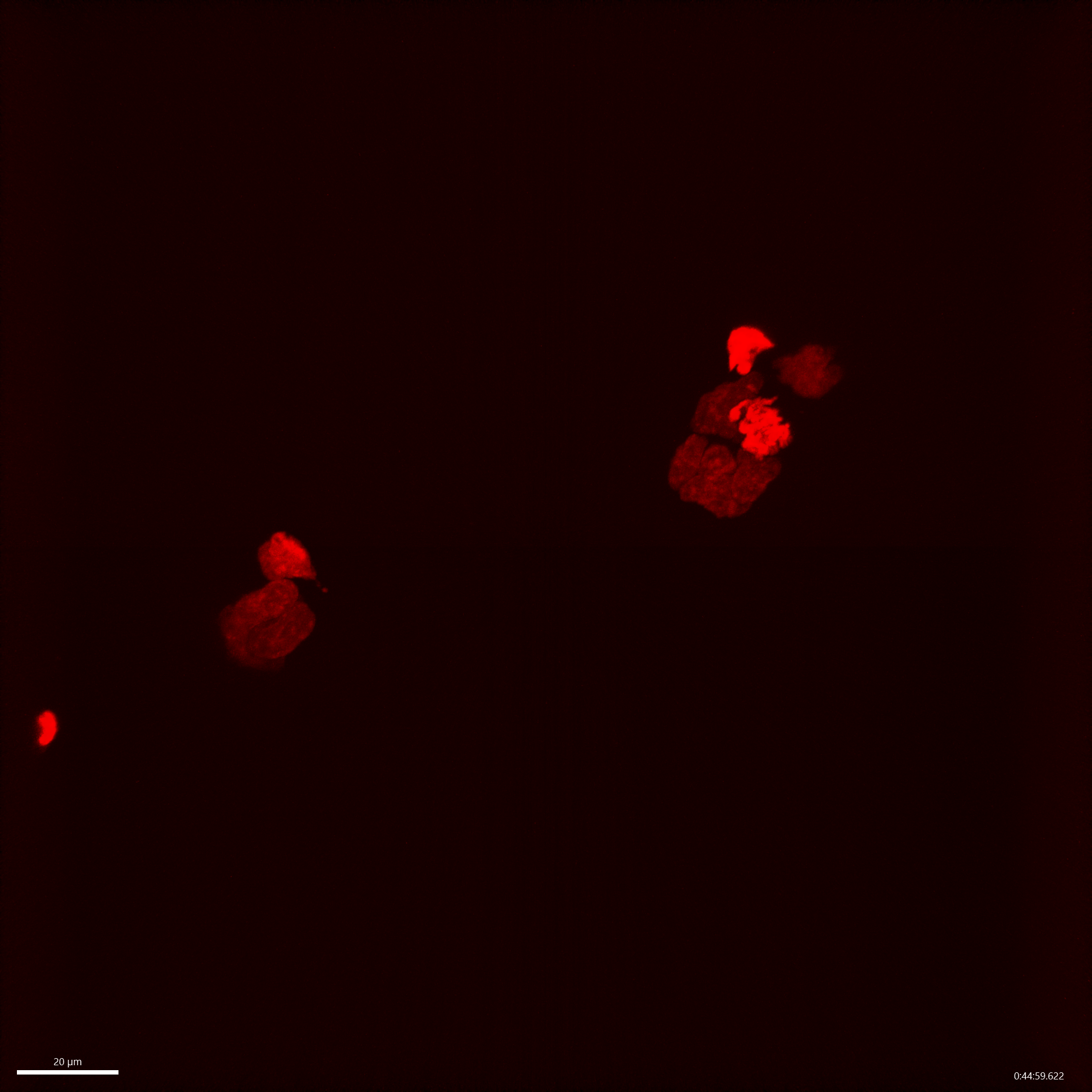

Supplement: Supplementary file 17 — Source data Fig. 3 [file 44320_2026_188_MOESM17_ESM.zip › Figure 3/3E/Live cell imaging Chr2+1 Aurora B-Inhibitor 6min.tif]

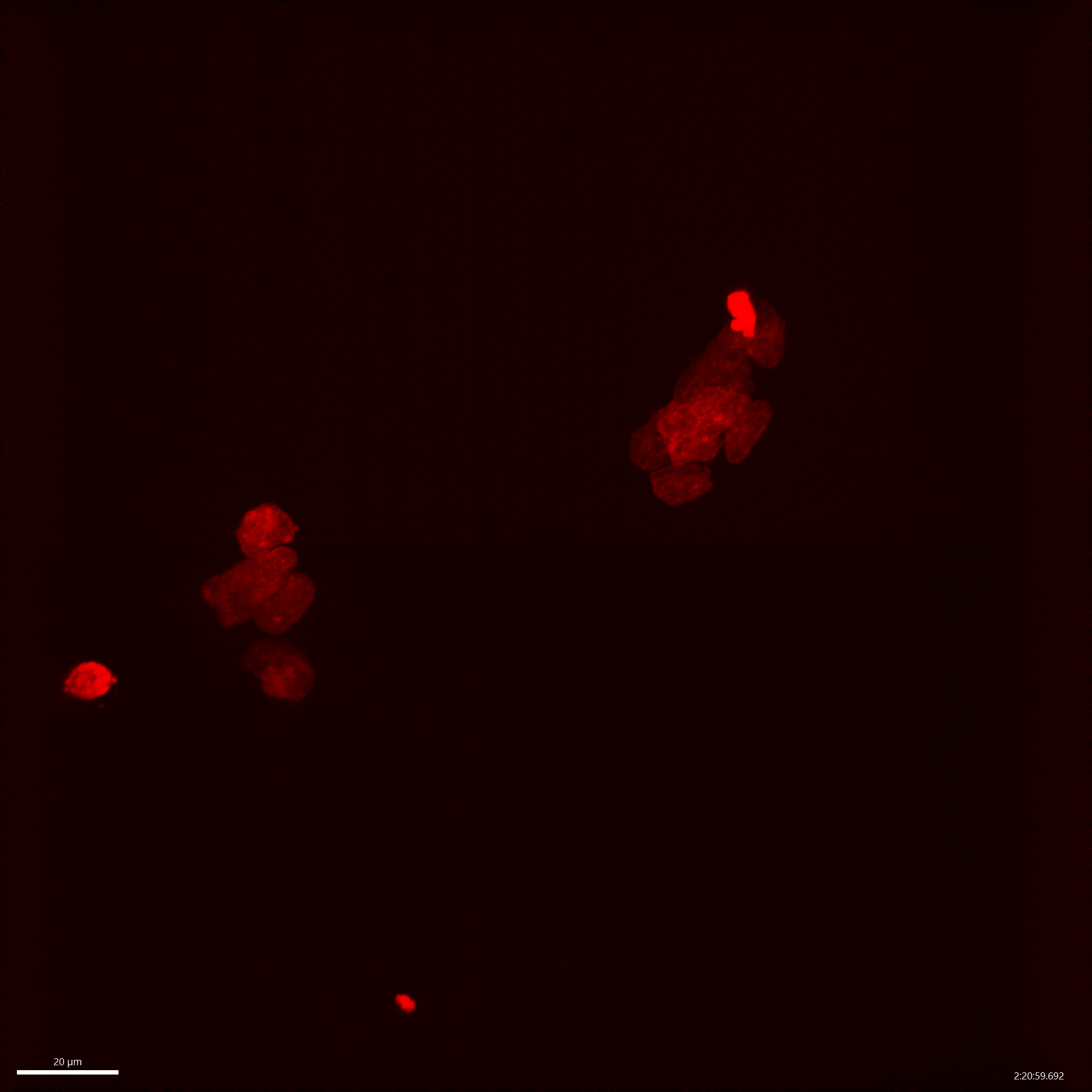

Supplement: Supplementary file 17 — Source data Fig. 3 [file 44320_2026_188_MOESM17_ESM.zip › Figure 3/3E/Live cell imaging Chr2+1 Aurora B-Inhibitor 72min.tif]

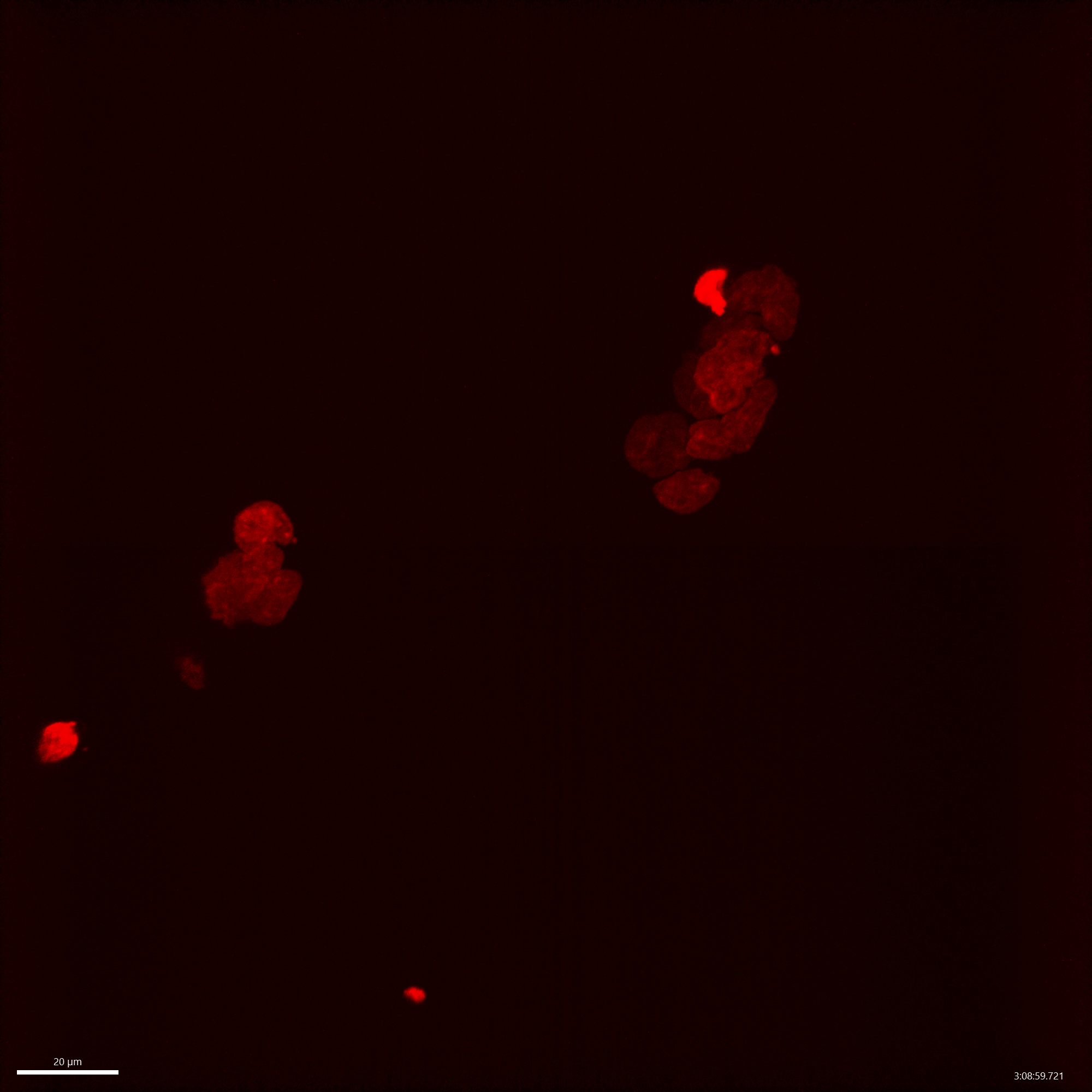

Supplement: Supplementary file 17 — Source data Fig. 3 [file 44320_2026_188_MOESM17_ESM.zip › Figure 3/3E/Live cell imaging Chr2+1 Aurora B-Inhibitor 96min.tif]

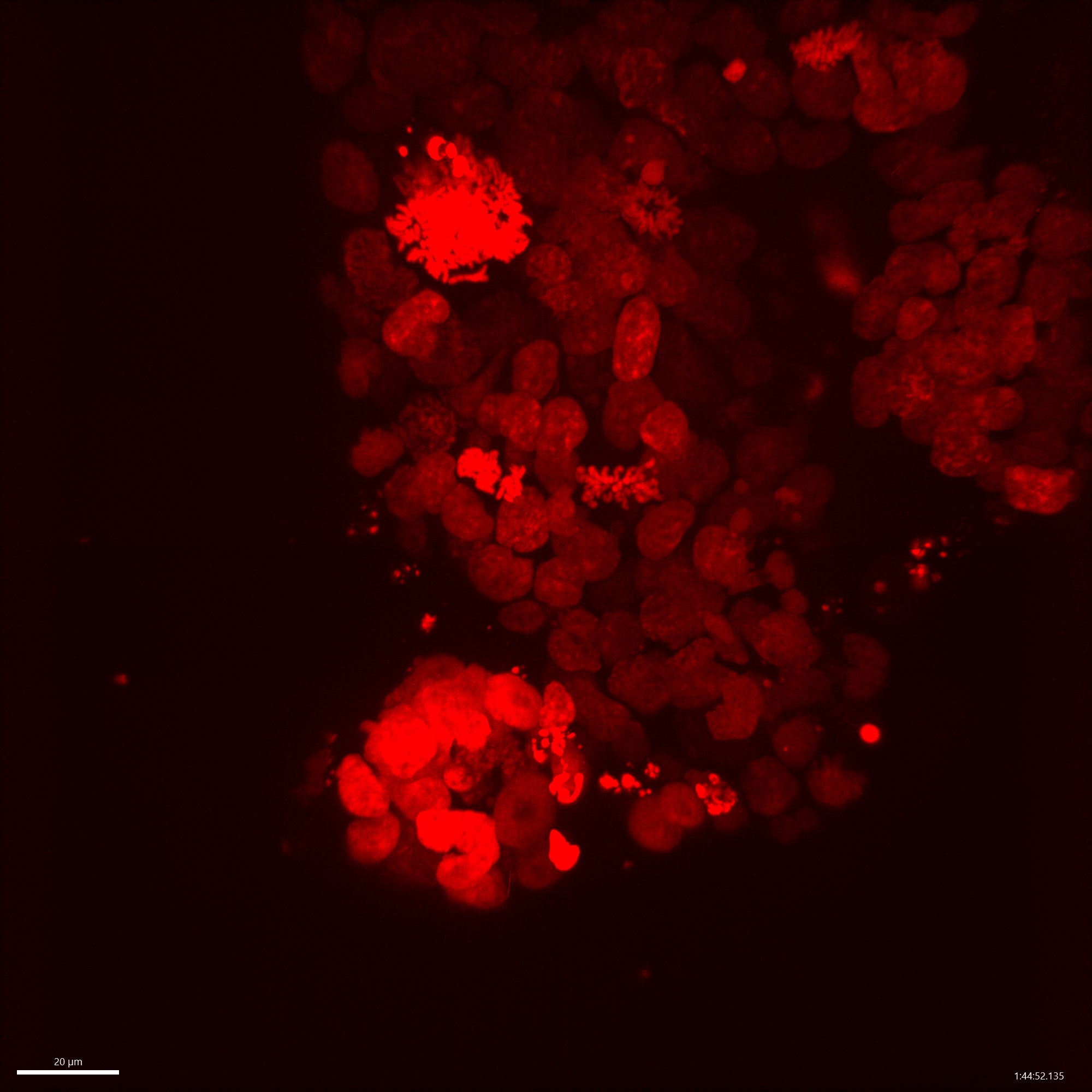

Supplement: Supplementary file 17 — Source data Fig. 3 [file 44320_2026_188_MOESM17_ESM.zip › Figure 3/3E/Live cell imaging Chr4+5 Aurora B-Inhibitor 0min.tif]

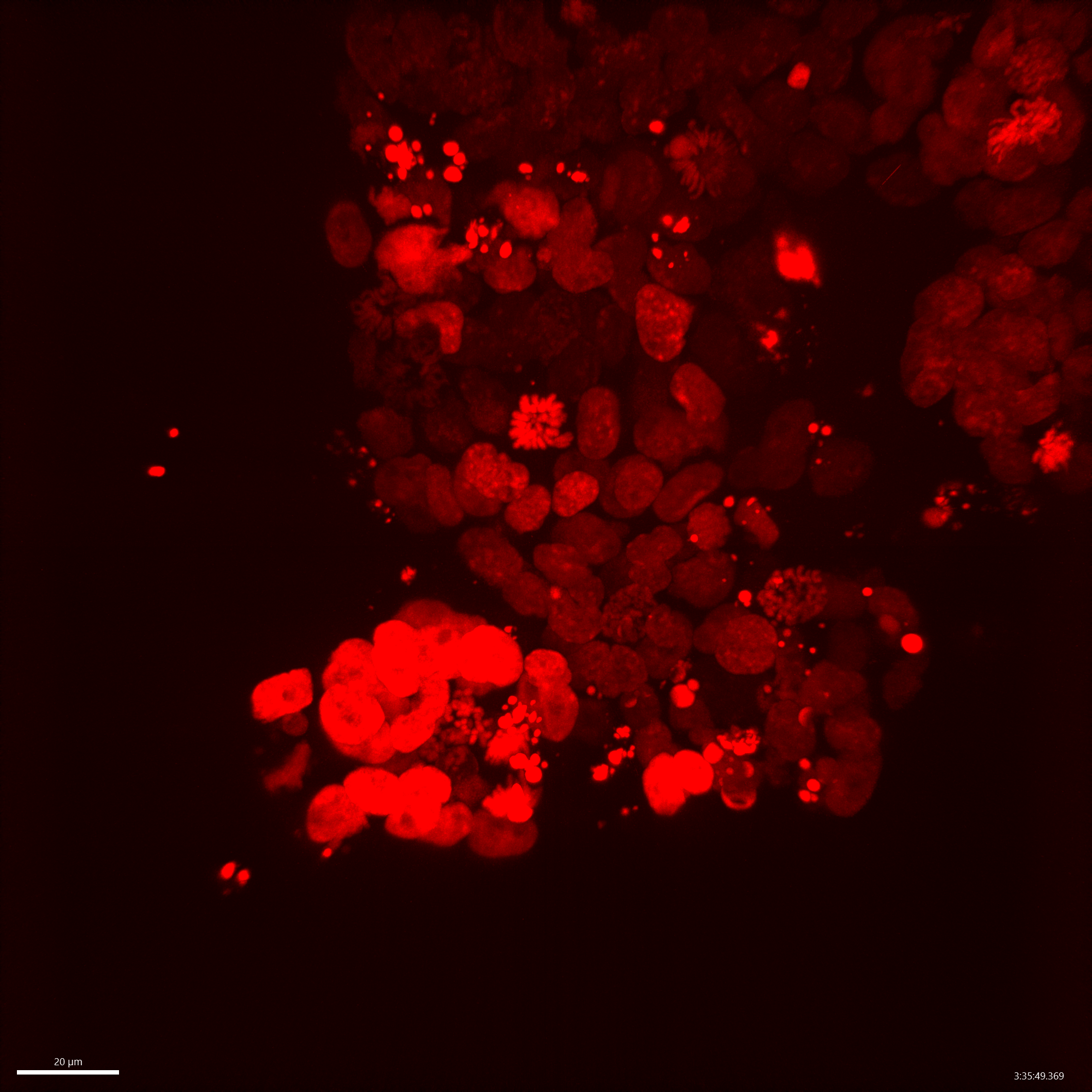

Supplement: Supplementary file 17 — Source data Fig. 3 [file 44320_2026_188_MOESM17_ESM.zip › Figure 3/3E/Live cell imaging Chr4+5 Aurora B-Inhibitor 104min.tif]

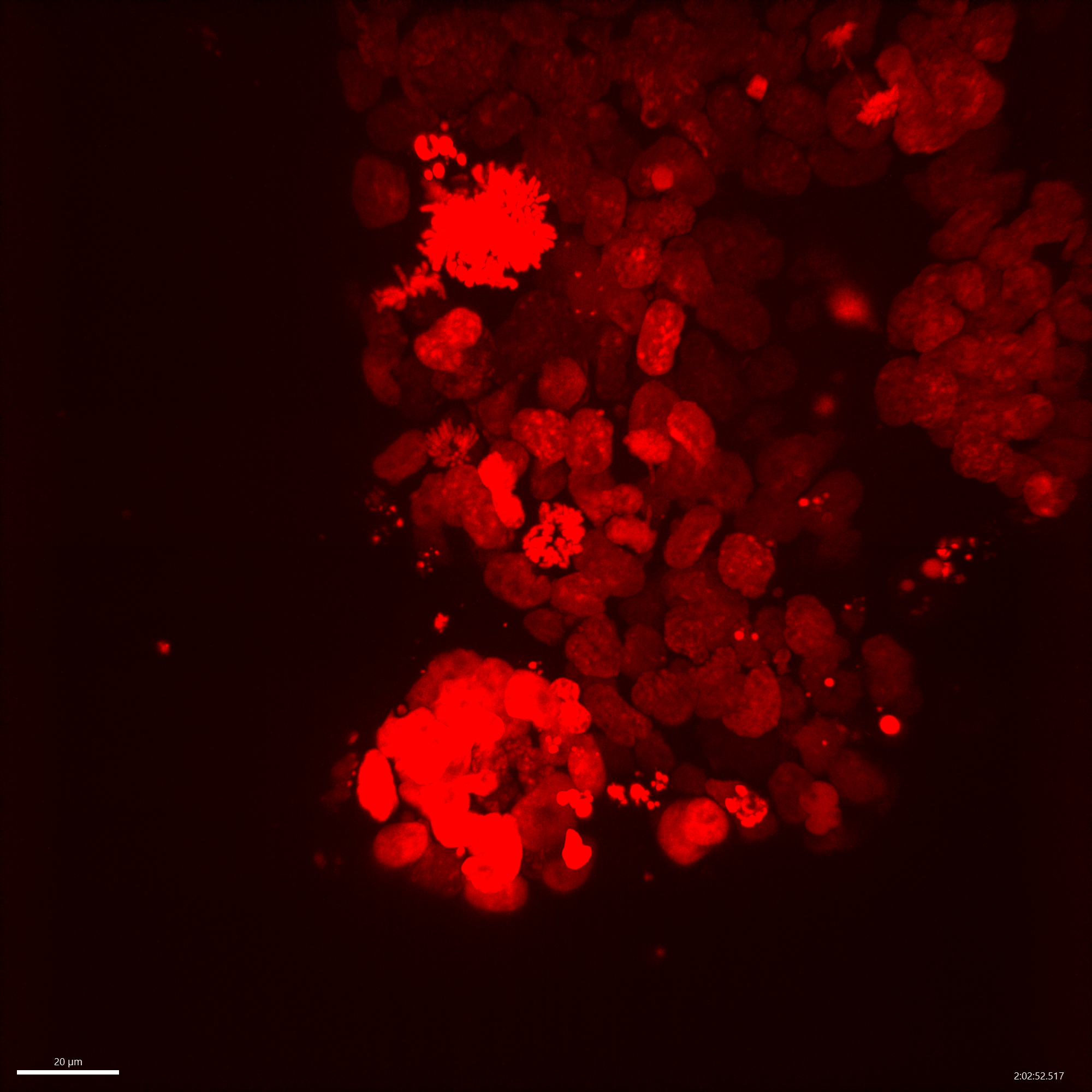

Supplement: Supplementary file 17 — Source data Fig. 3 [file 44320_2026_188_MOESM17_ESM.zip › Figure 3/3E/Live cell imaging Chr4+5 Aurora B-Inhibitor 18min.tif]

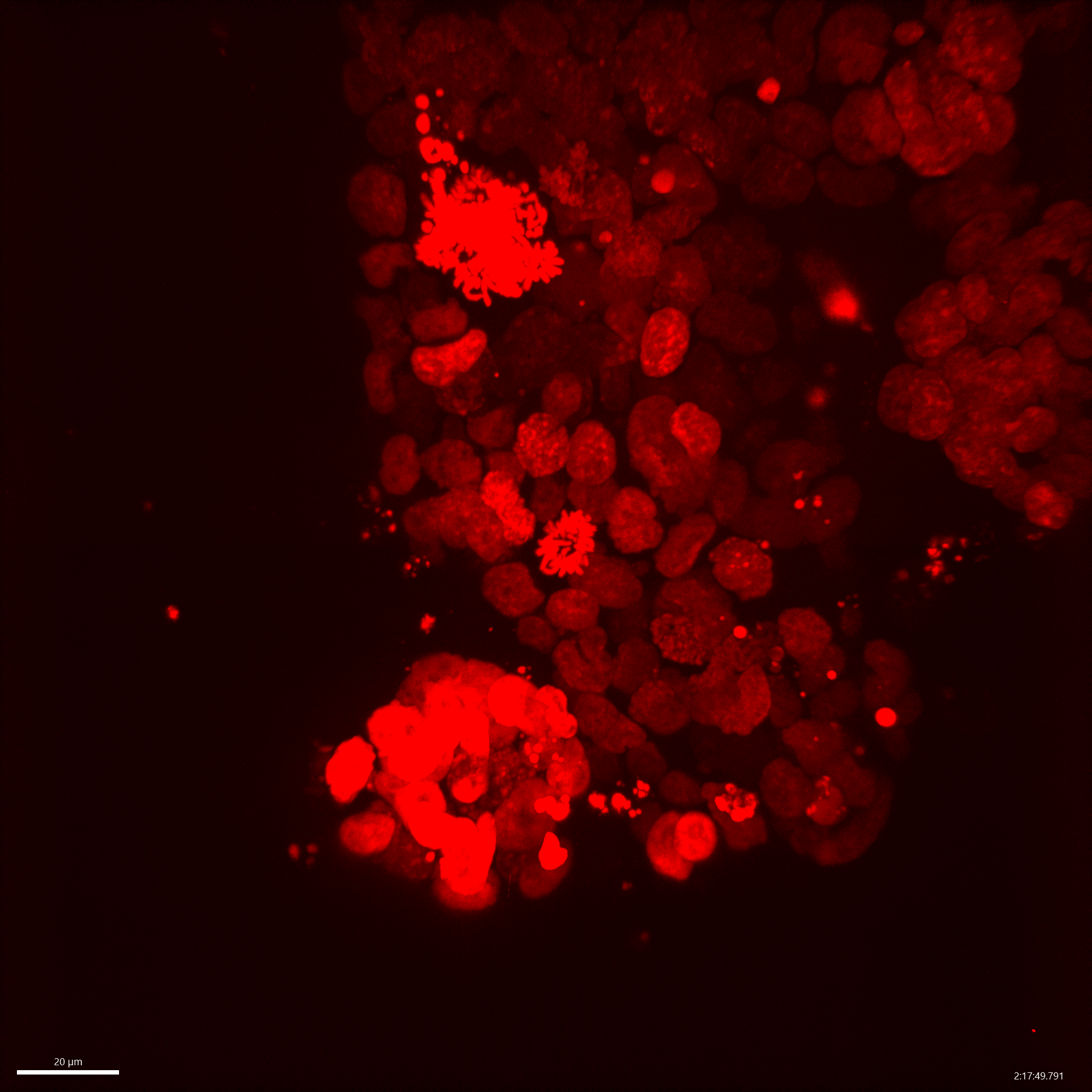

Supplement: Supplementary file 17 — Source data Fig. 3 [file 44320_2026_188_MOESM17_ESM.zip › Figure 3/3E/Live cell imaging Chr4+5 Aurora B-Inhibitor 33min.tif]

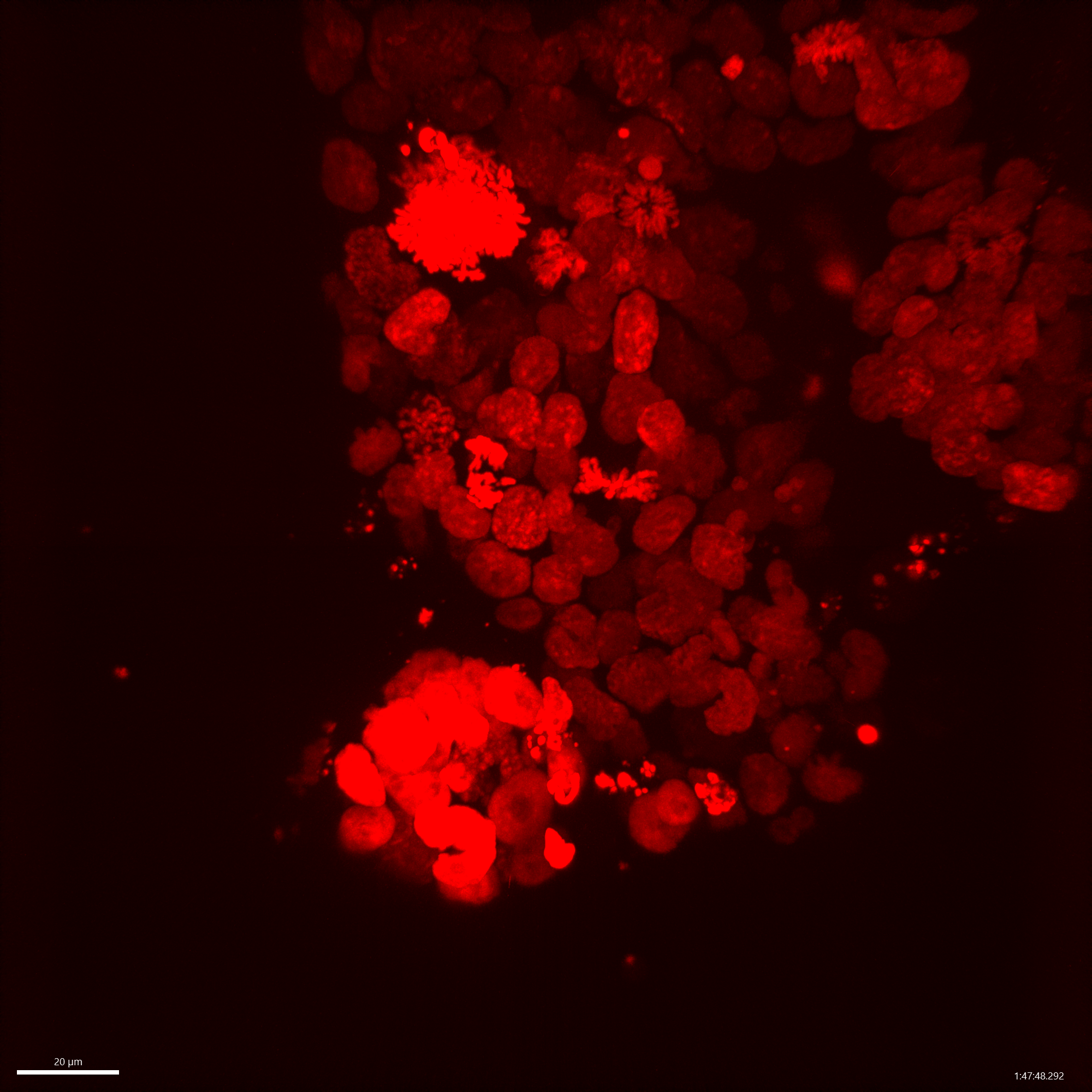

Supplement: Supplementary file 17 — Source data Fig. 3 [file 44320_2026_188_MOESM17_ESM.zip › Figure 3/3E/Live cell imaging Chr4+5 Aurora B-Inhibitor 3min.tif]

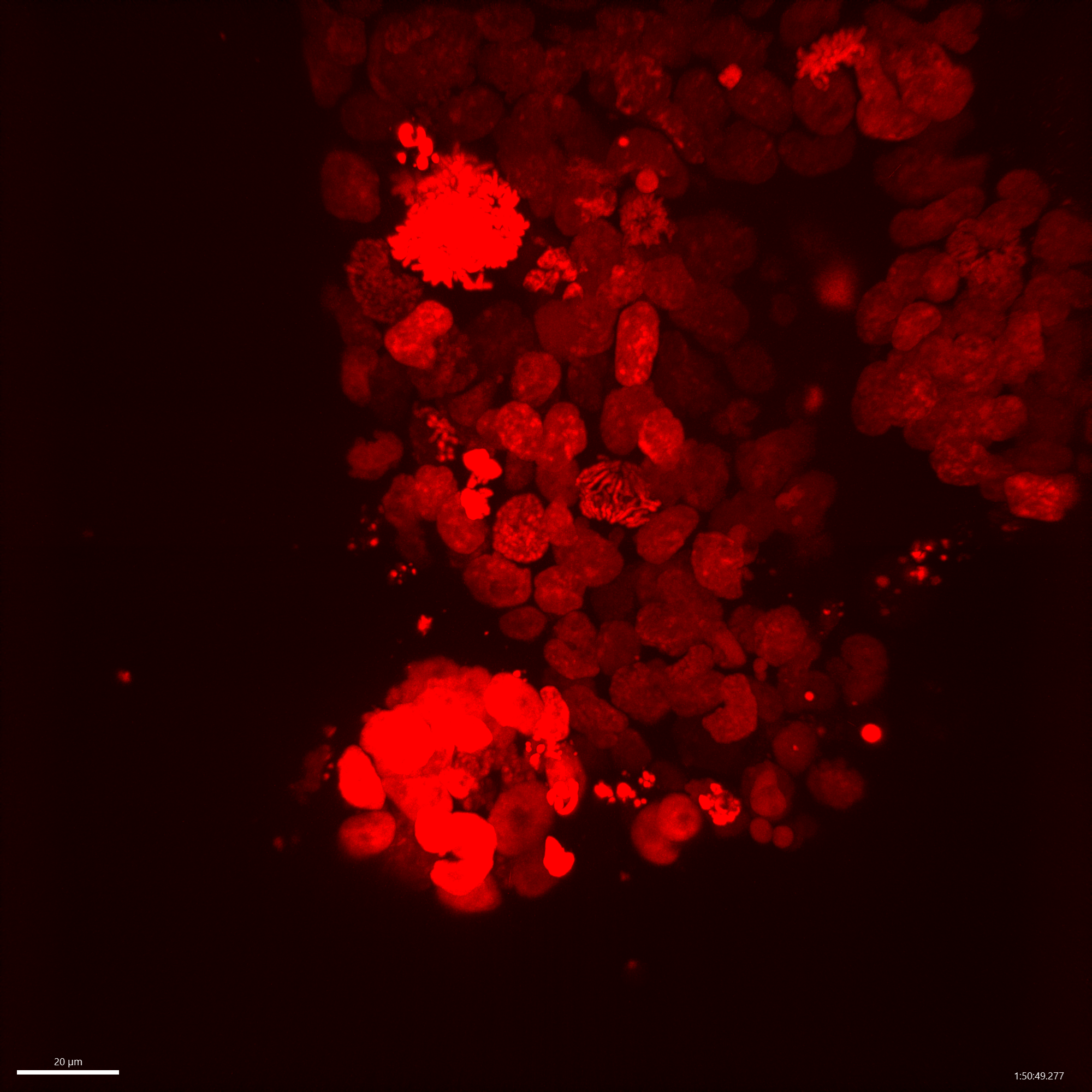

Supplement: Supplementary file 17 — Source data Fig. 3 [file 44320_2026_188_MOESM17_ESM.zip › Figure 3/3E/Live cell imaging Chr4+5 Aurora B-Inhibitor 6min.tif]

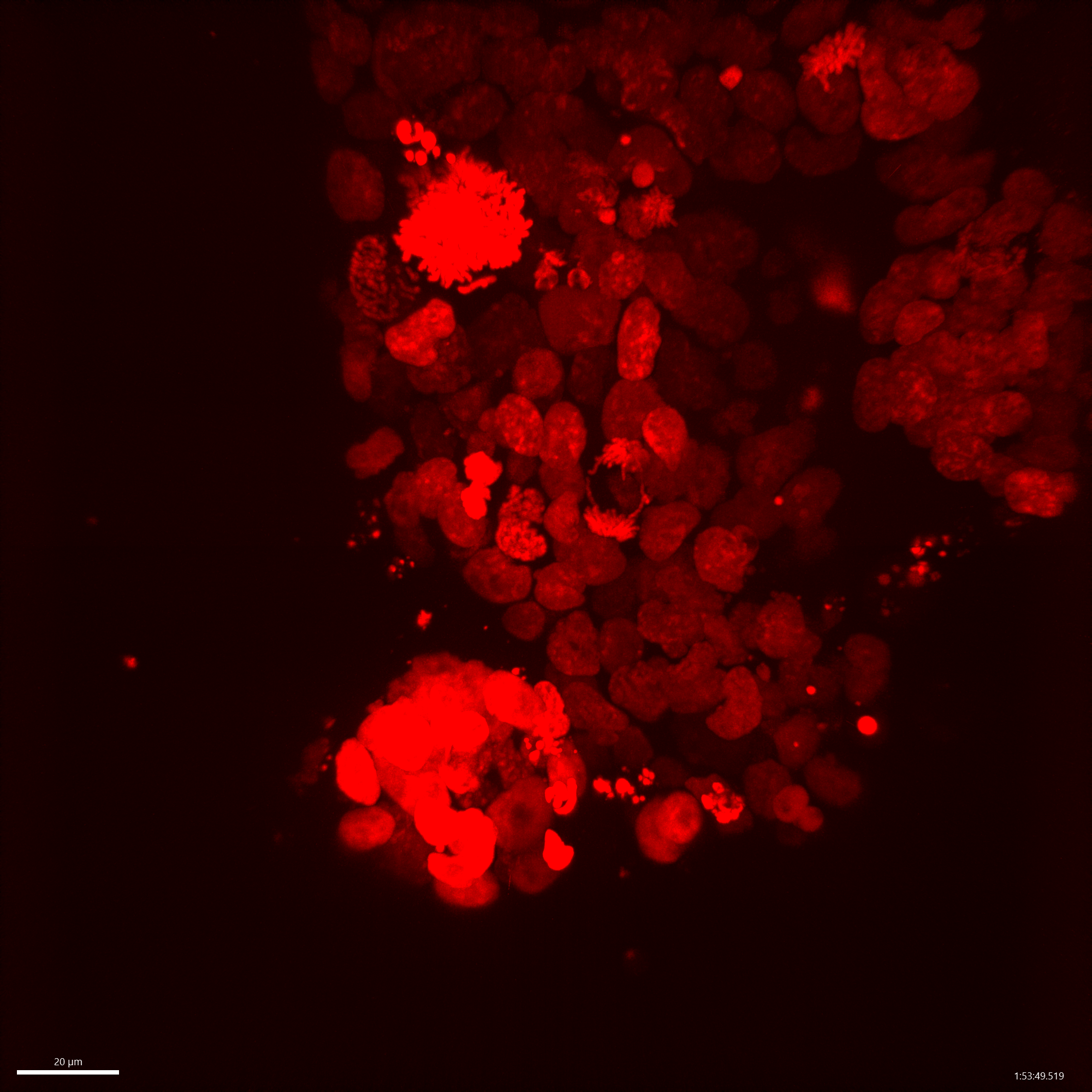

Supplement: Supplementary file 17 — Source data Fig. 3 [file 44320_2026_188_MOESM17_ESM.zip › Figure 3/3E/Live cell imaging Chr4+5 Aurora B-Inhibitor 9min.tif]

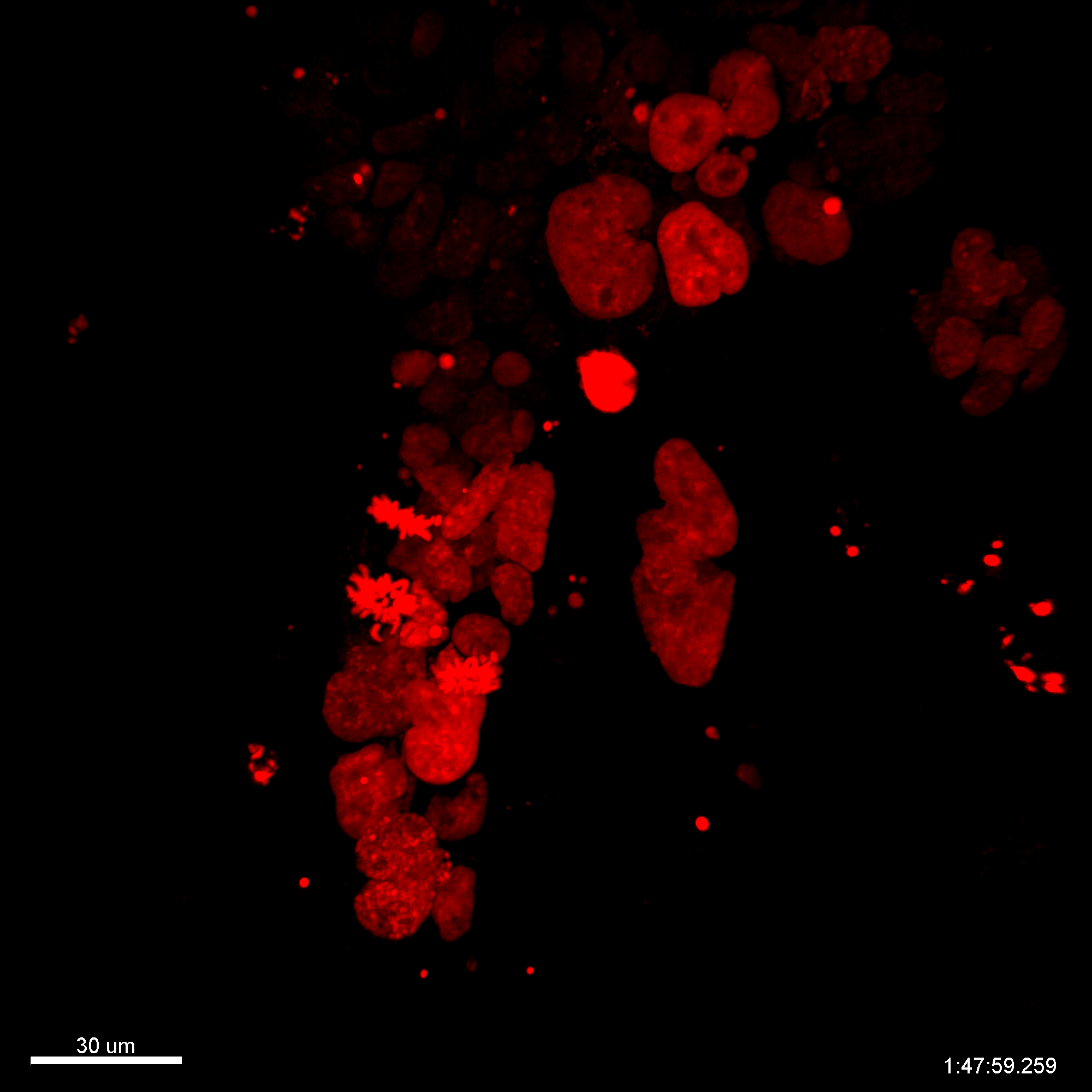

Supplement: Supplementary file 17 — Source data Fig. 3 [file 44320_2026_188_MOESM17_ESM.zip › Figure 3/3E/Live cell imaging WT Aurora B-Inhibitor 0min.tif]

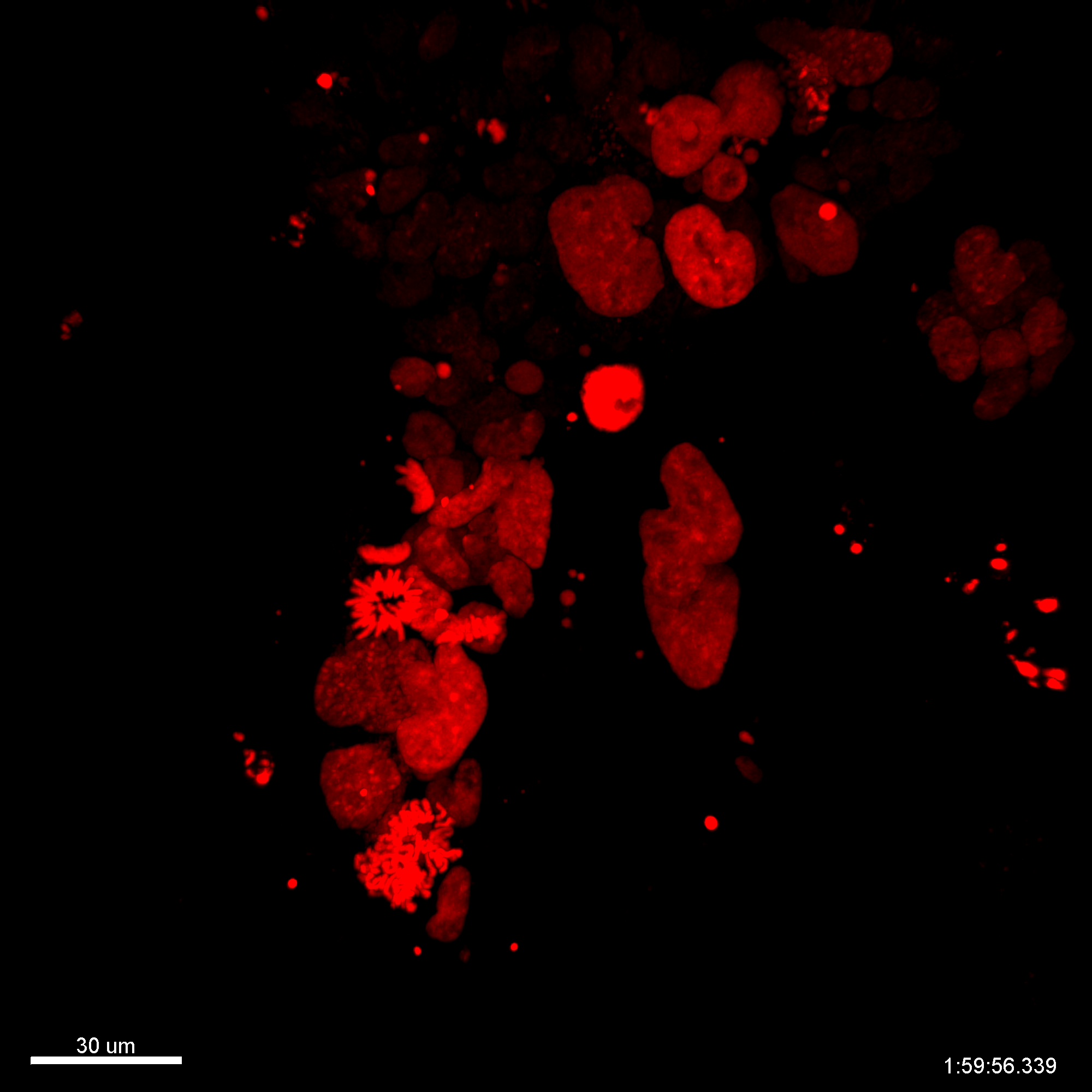

Supplement: Supplementary file 17 — Source data Fig. 3 [file 44320_2026_188_MOESM17_ESM.zip › Figure 3/3E/Live cell imaging WT Aurora B-Inhibitor 12min.tif]

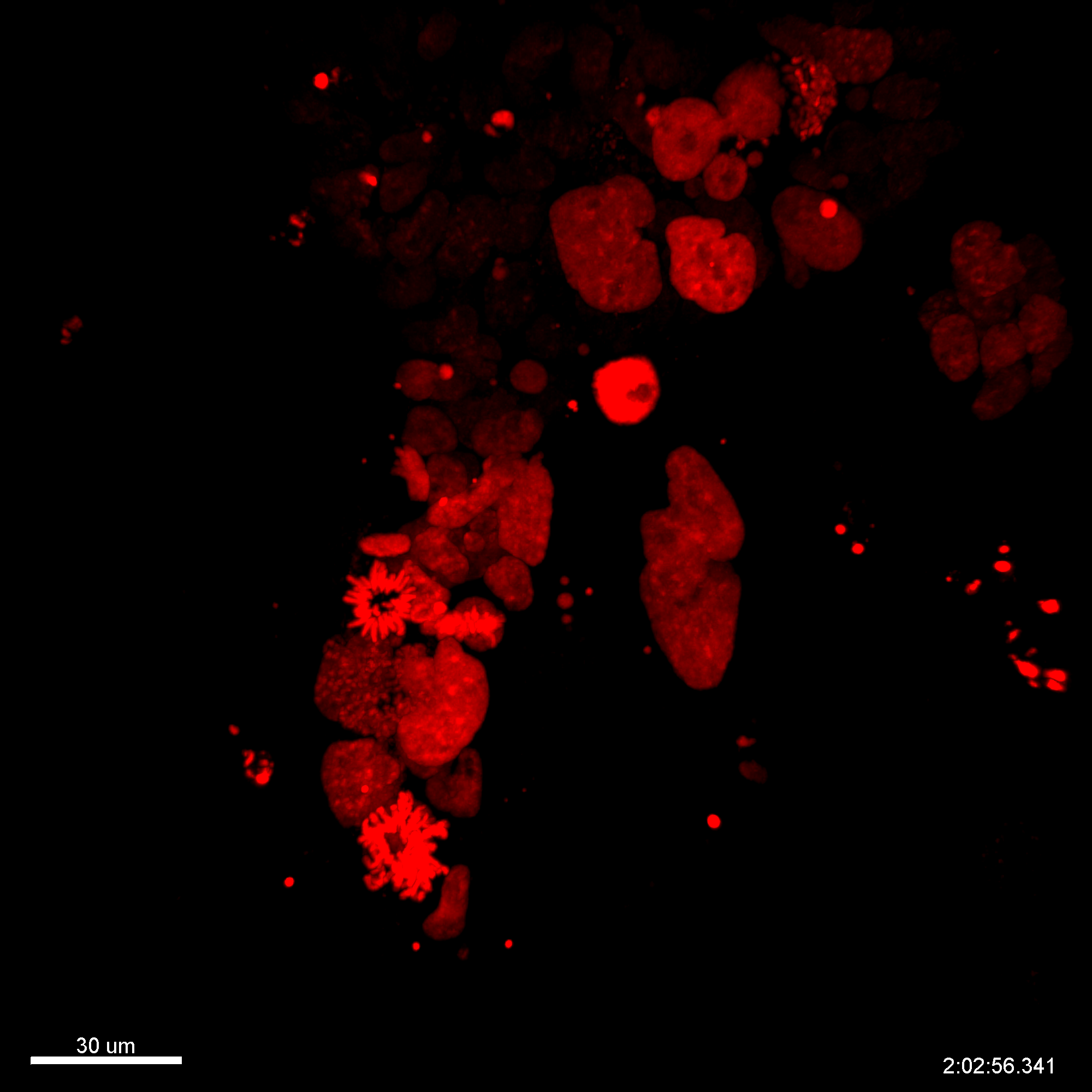

Supplement: Supplementary file 17 — Source data Fig. 3 [file 44320_2026_188_MOESM17_ESM.zip › Figure 3/3E/Live cell imaging WT Aurora B-Inhibitor 15min.tif]

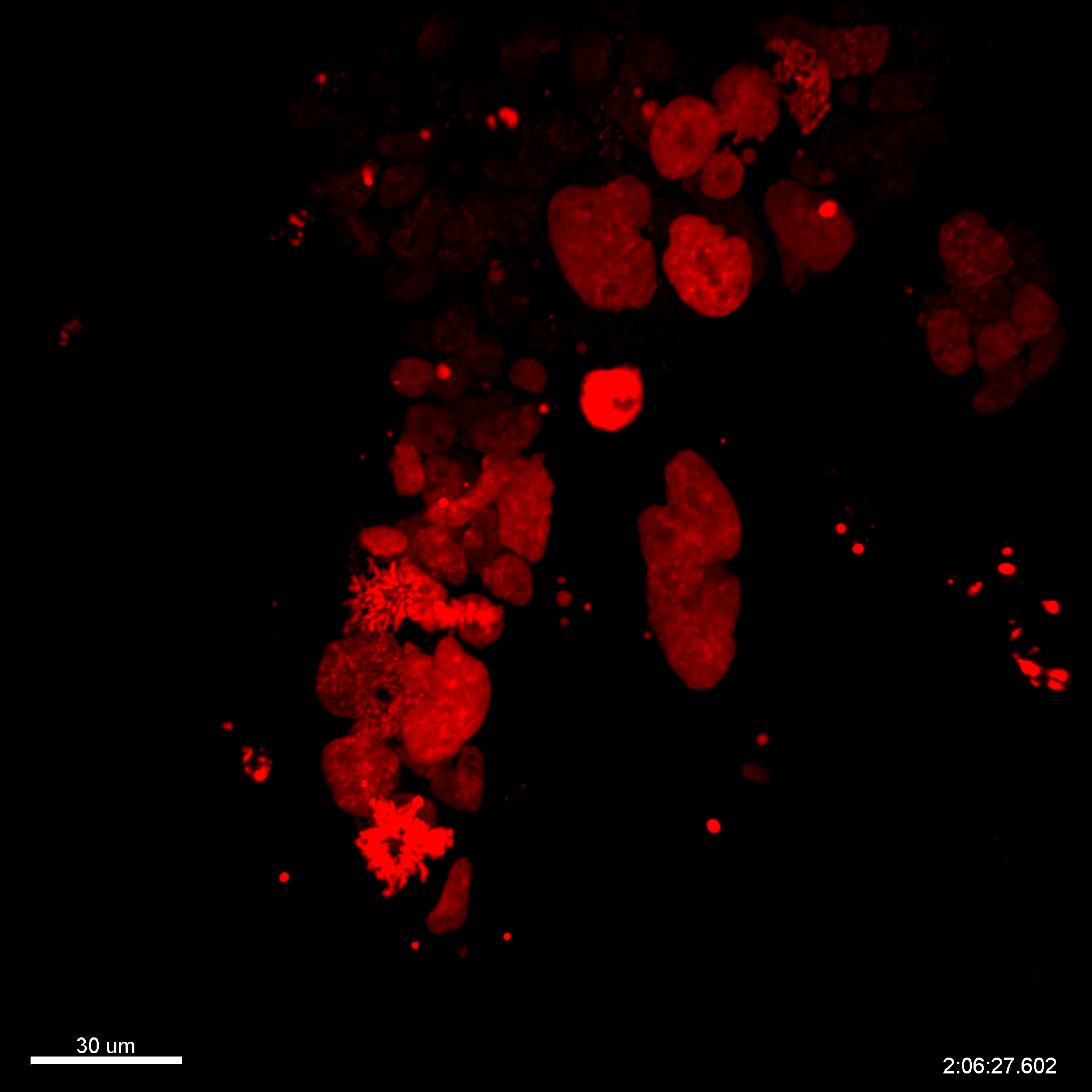

Supplement: Supplementary file 17 — Source data Fig. 3 [file 44320_2026_188_MOESM17_ESM.zip › Figure 3/3E/Live cell imaging WT Aurora B-Inhibitor 18min.tif]

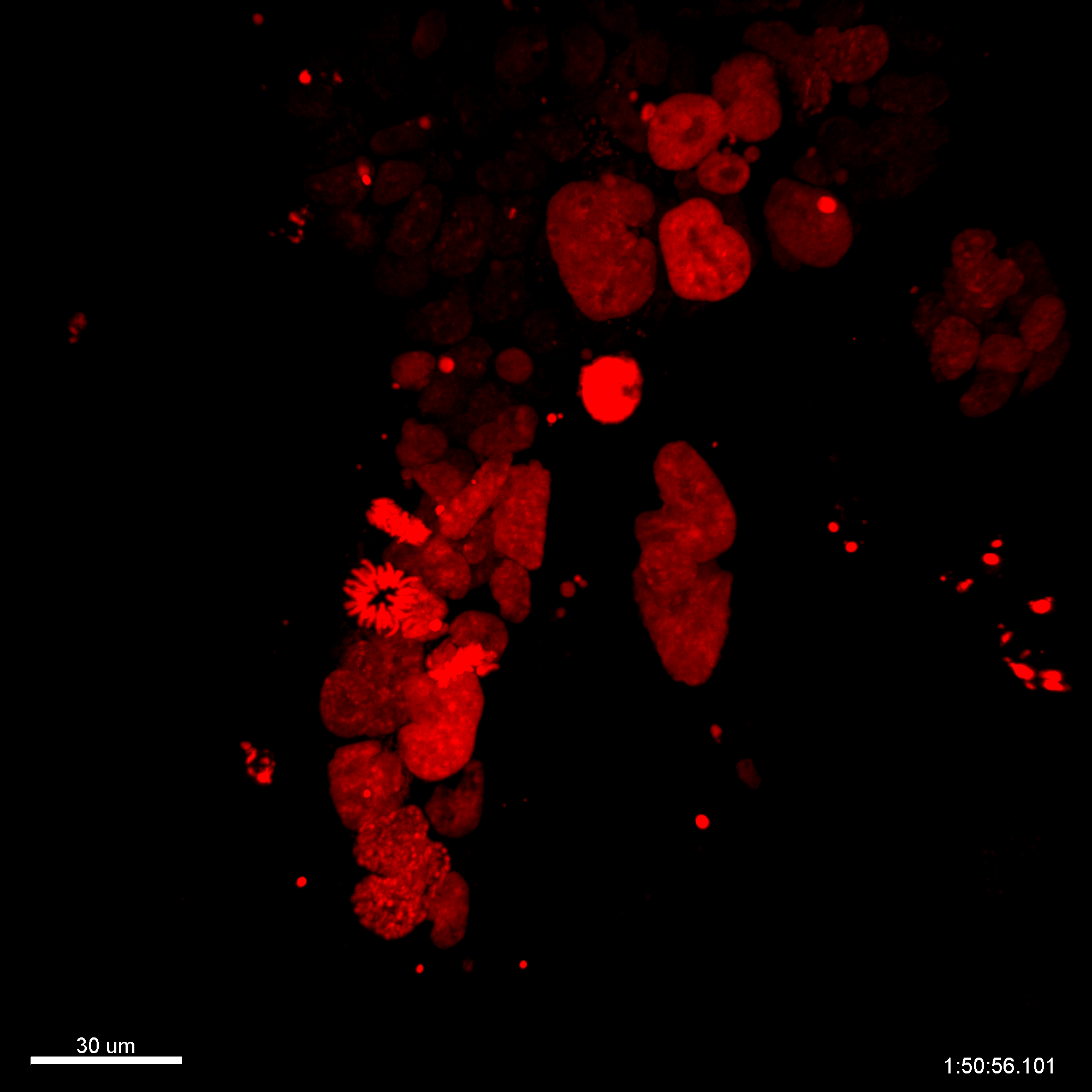

Supplement: Supplementary file 17 — Source data Fig. 3 [file 44320_2026_188_MOESM17_ESM.zip › Figure 3/3E/Live cell imaging WT Aurora B-Inhibitor 3min.tif]

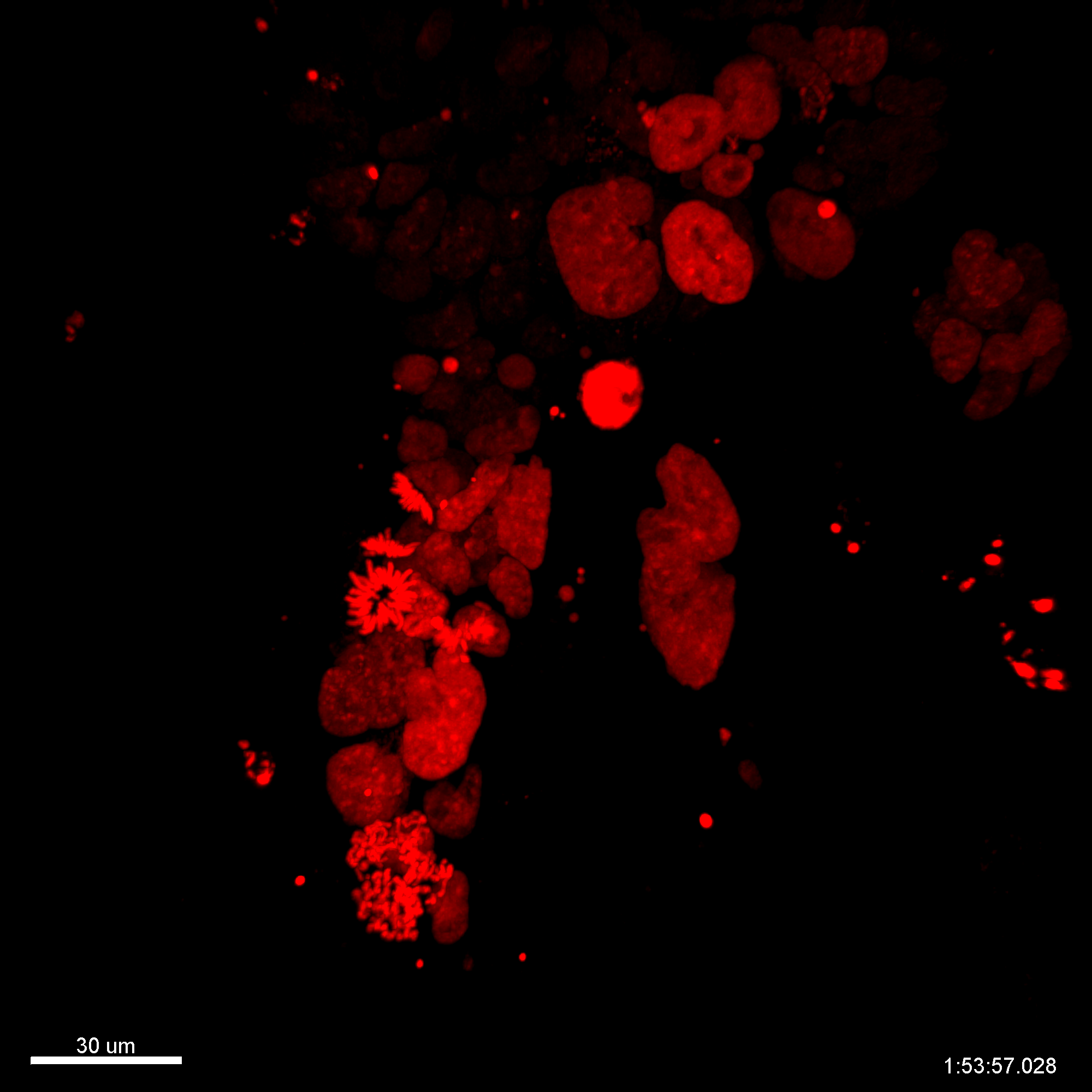

Supplement: Supplementary file 17 — Source data Fig. 3 [file 44320_2026_188_MOESM17_ESM.zip › Figure 3/3E/Live cell imaging WT Aurora B-Inhibitor 6min.tif]

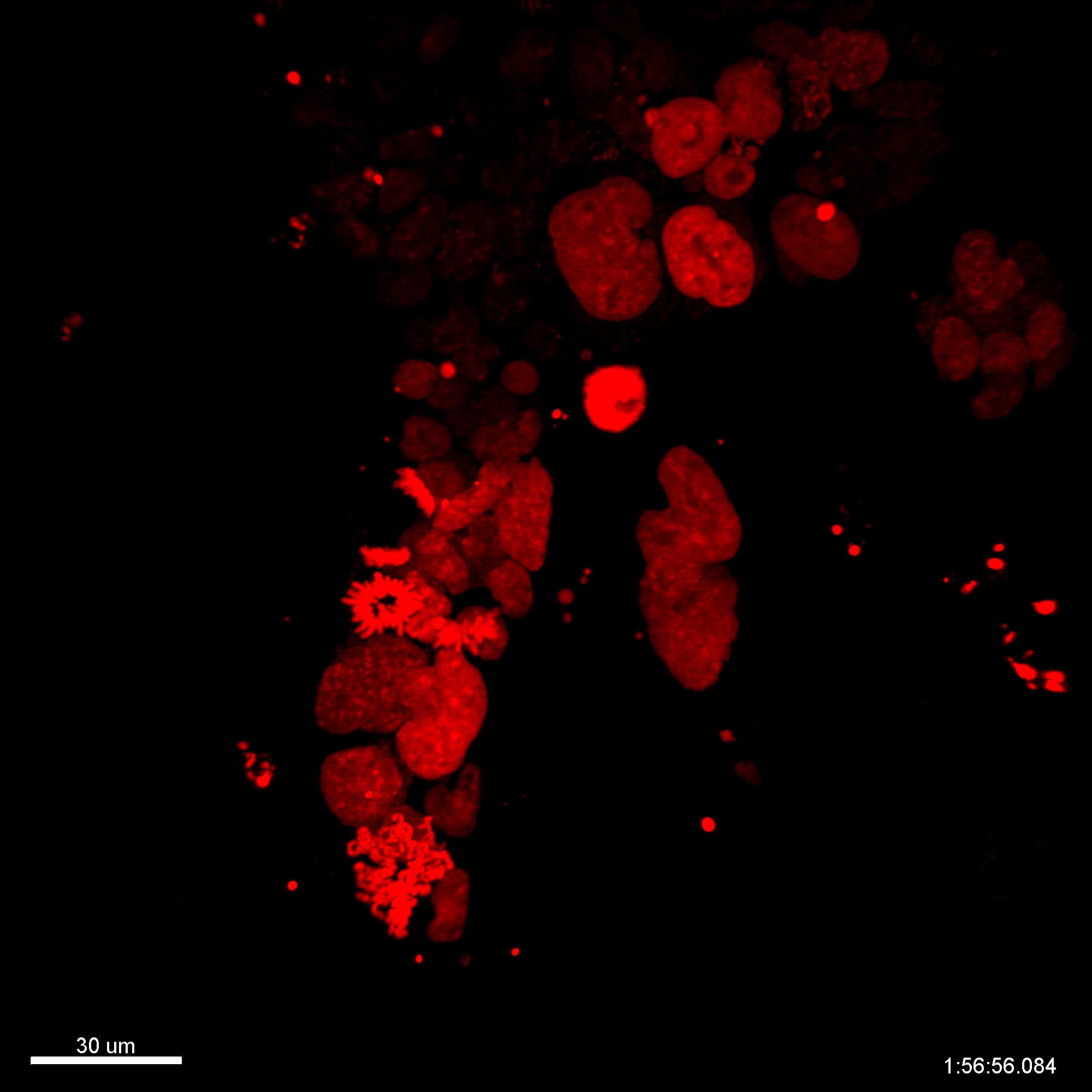

Supplement: Supplementary file 17 — Source data Fig. 3 [file 44320_2026_188_MOESM17_ESM.zip › Figure 3/3E/Live cell imaging WT Aurora B-Inhibitor 9min.tif]

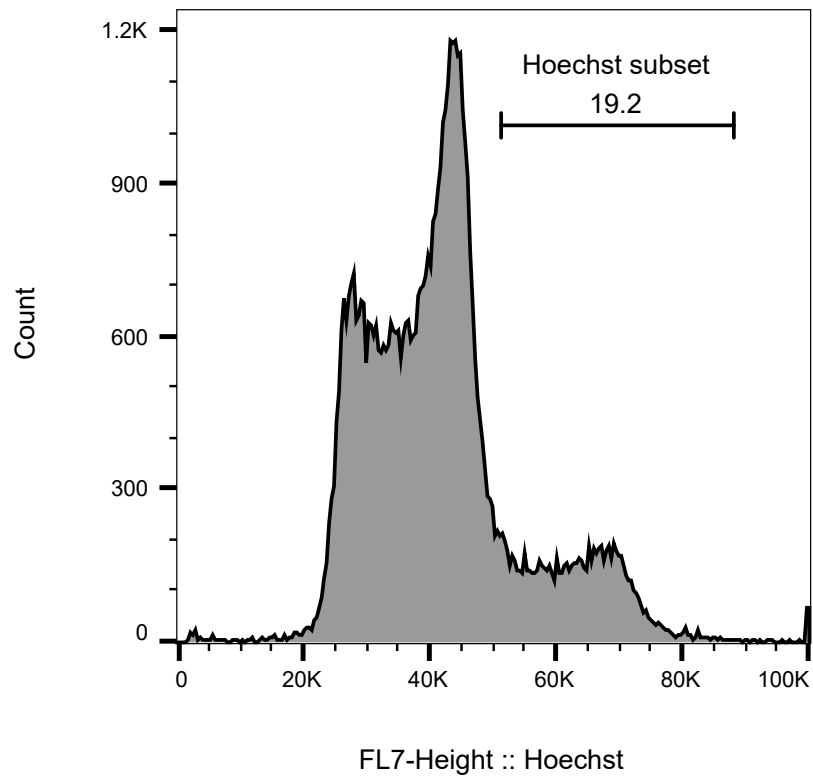

Chr2+1-Aurora B Inhibitor-D18

Supplement: Supplementary file 17 — Source data Fig. 3 [file 44320_2026_188_MOESM17_ESM.zip › Figure 3/3F/FACS Chr2+1 Aurora B-Inhibitor D18.pdf]

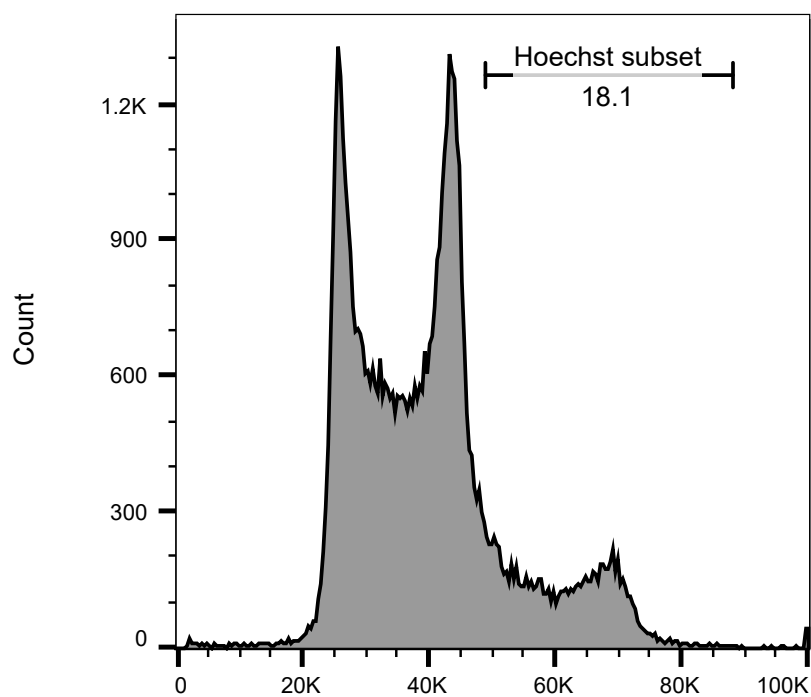

FL7-Height :: Hoechst

Chr2+1-D0

Supplement: Supplementary file 17 — Source data Fig. 3 [file 44320_2026_188_MOESM17_ESM.zip › Figure 3/3F/FASC Chr2+1 D0.pdf]

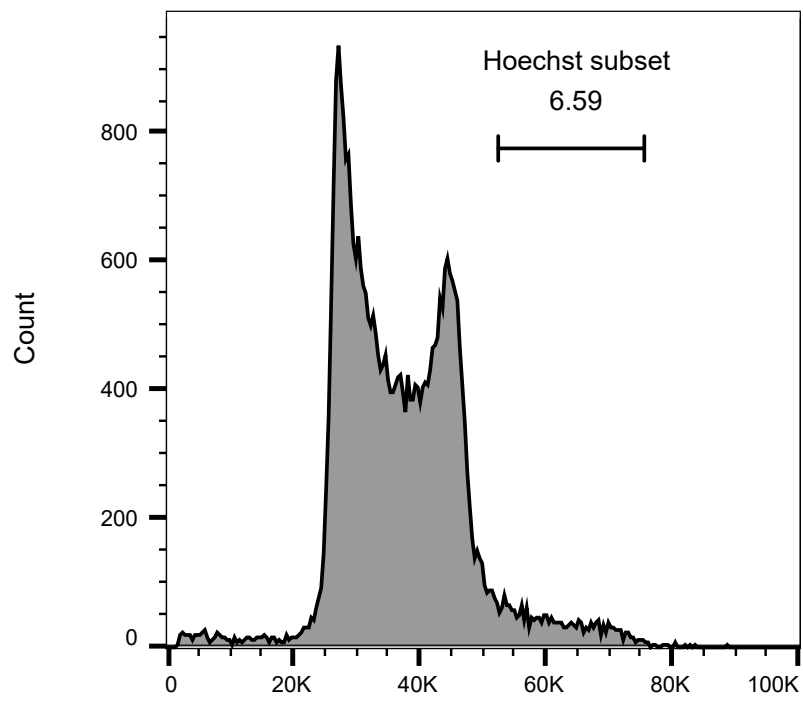

FL7-Height :: Hoechst

Chr2+1-D18

Supplement: Supplementary file 17 — Source data Fig. 3 [file 44320_2026_188_MOESM17_ESM.zip › Figure 3/3F/FASC Chr2+1 D18.pdf]

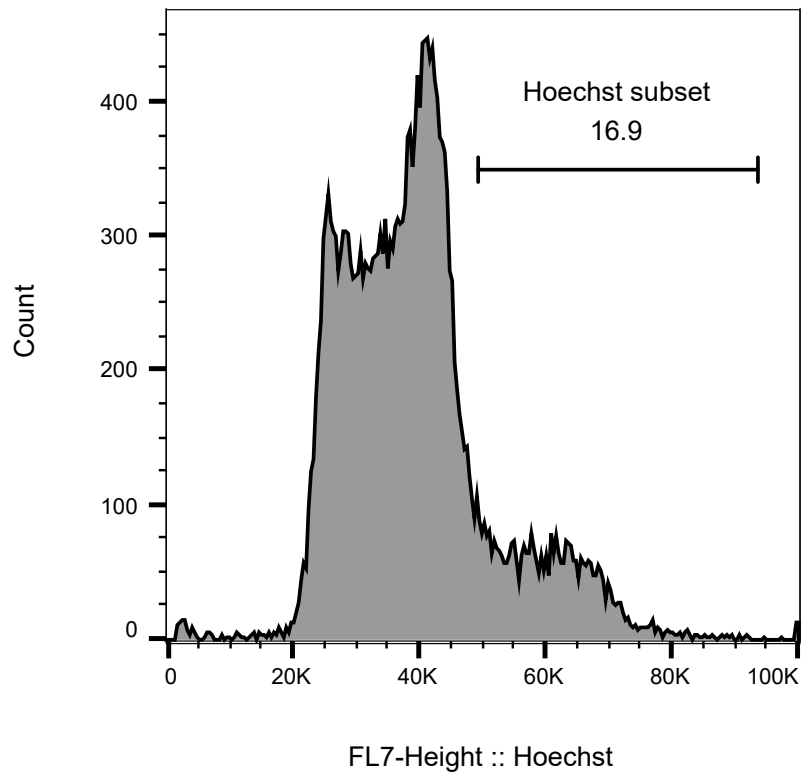

Chr4+5-Aurora B Inhibitor-D18

Supplement: Supplementary file 17 — Source data Fig. 3 [file 44320_2026_188_MOESM17_ESM.zip › Figure 3/3F/FASC Chr4+5 Aurora B-Inhibitor D18.pdf]
