# Supplementary figures and images for: Chromosome length is constrained by spindle scaling to ensure faithful mitosis in mammals (part 2 of 2)
Source: Mol Syst Biol. 2026 Jan 15;22(4):480–96. doi: 10.1038/s44320-026-00188-8 (PMC13047055; doi:10.1038/s44320-026-00188-8)

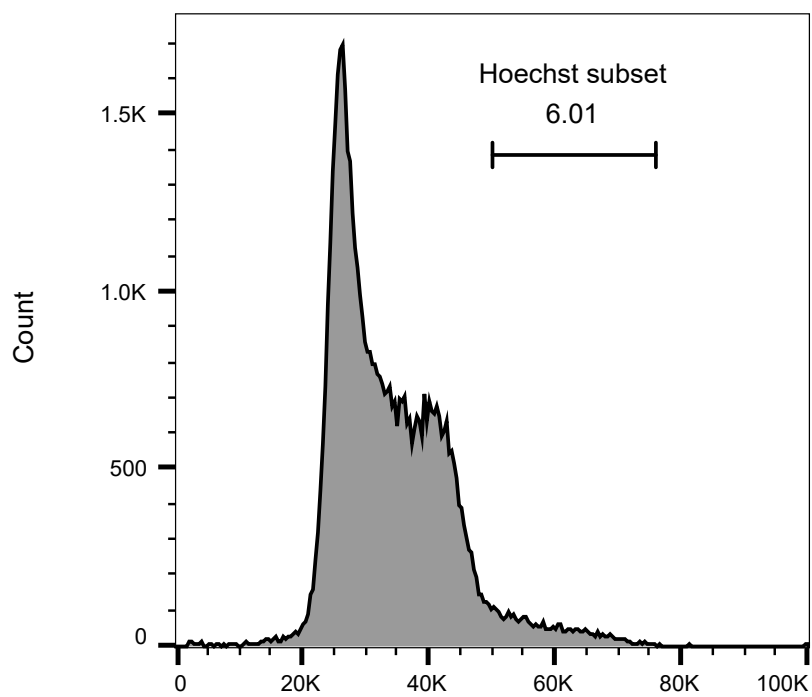

FL7-Height :: Hoechst

Chr4+5-D0

Supplement: Supplementary file 17 — Source data Fig. 3 [file 44320_2026_188_MOESM17_ESM.zip › Figure 3/3F/FASC Chr4+5 D0.pdf]

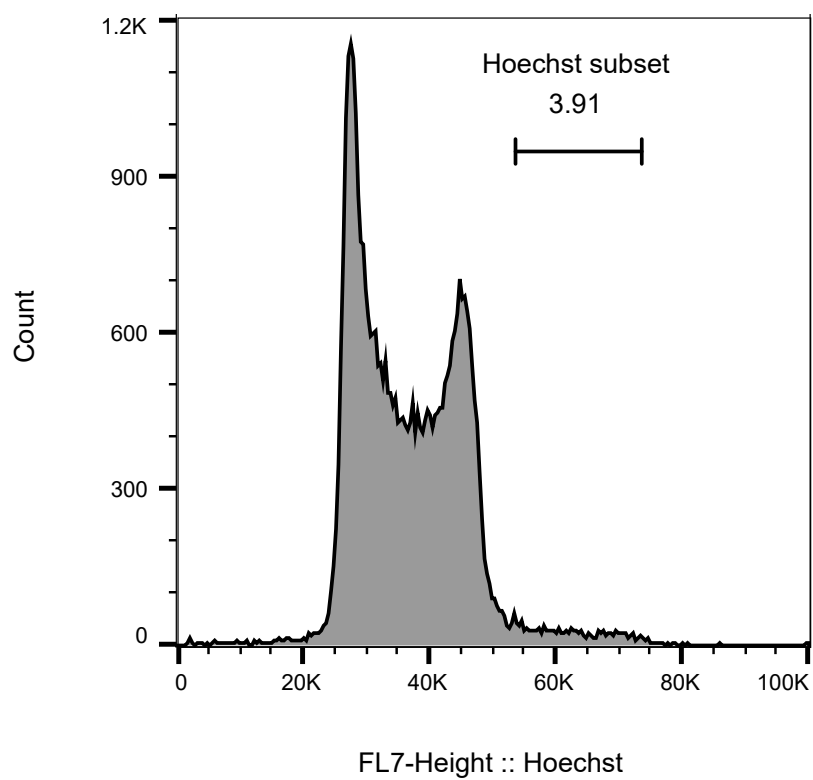

Chr4+5-D18

Supplement: Supplementary file 17 — Source data Fig. 3 [file 44320_2026_188_MOESM17_ESM.zip › Figure 3/3F/FASC Chr4+5 D18.pdf]

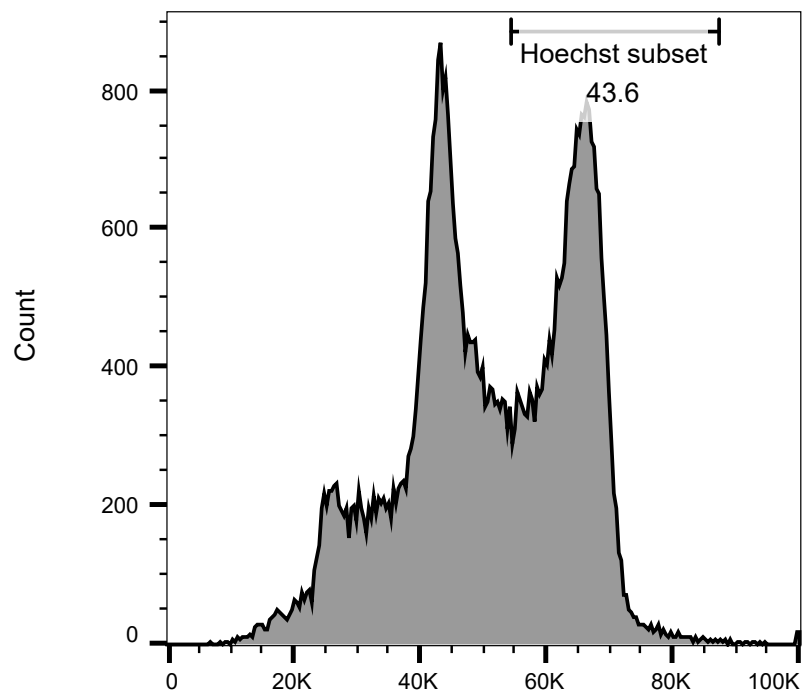

FL7-Height :: Hoechst

WT-4N

Supplement: Supplementary file 17 — Source data Fig. 3 [file 44320_2026_188_MOESM17_ESM.zip › Figure 3/3F/FASC WT 4N.pdf]

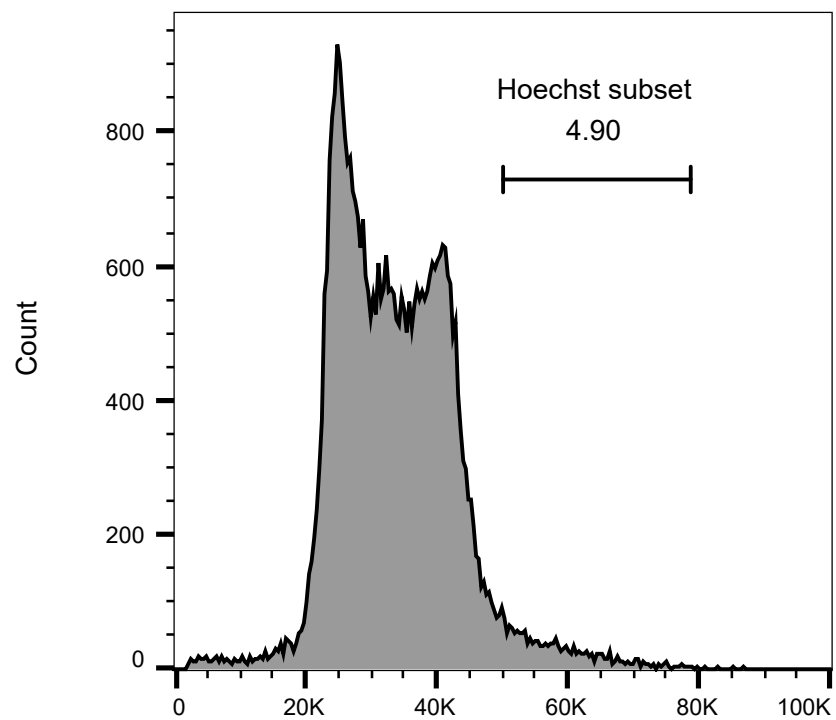

FL7-Height :: Hoechst

WT-Aurora B Inhibitor-D18

Supplement: Supplementary file 17 — Source data Fig. 3 [file 44320_2026_188_MOESM17_ESM.zip › Figure 3/3F/FASC WT Aurora B-Inhibitor D18.pdf]

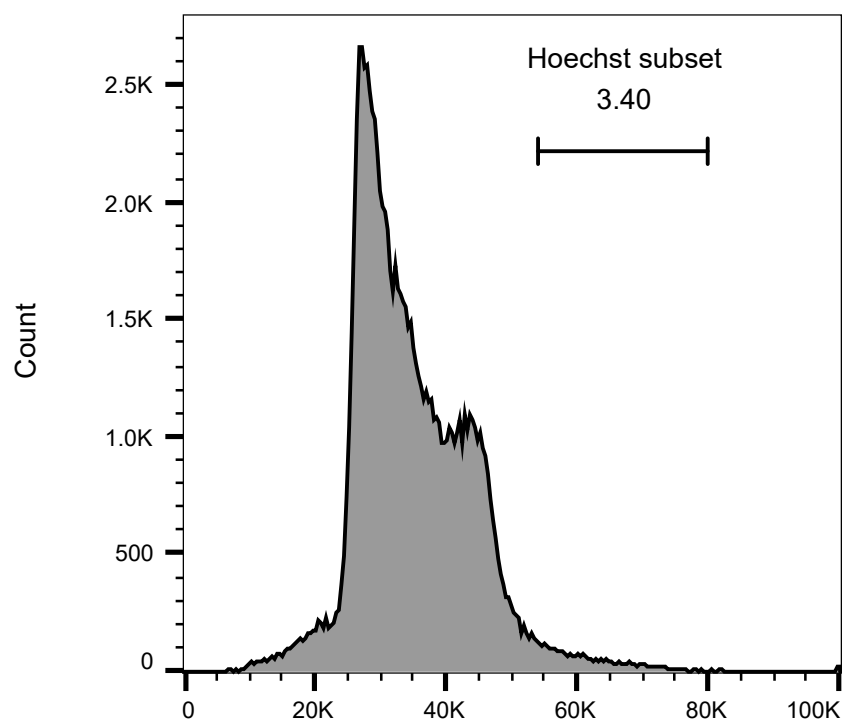

FL7-Height :: Hoechst

WT-D0

Supplement: Supplementary file 17 — Source data Fig. 3 [file 44320_2026_188_MOESM17_ESM.zip › Figure 3/3F/FASC WT D0.pdf]

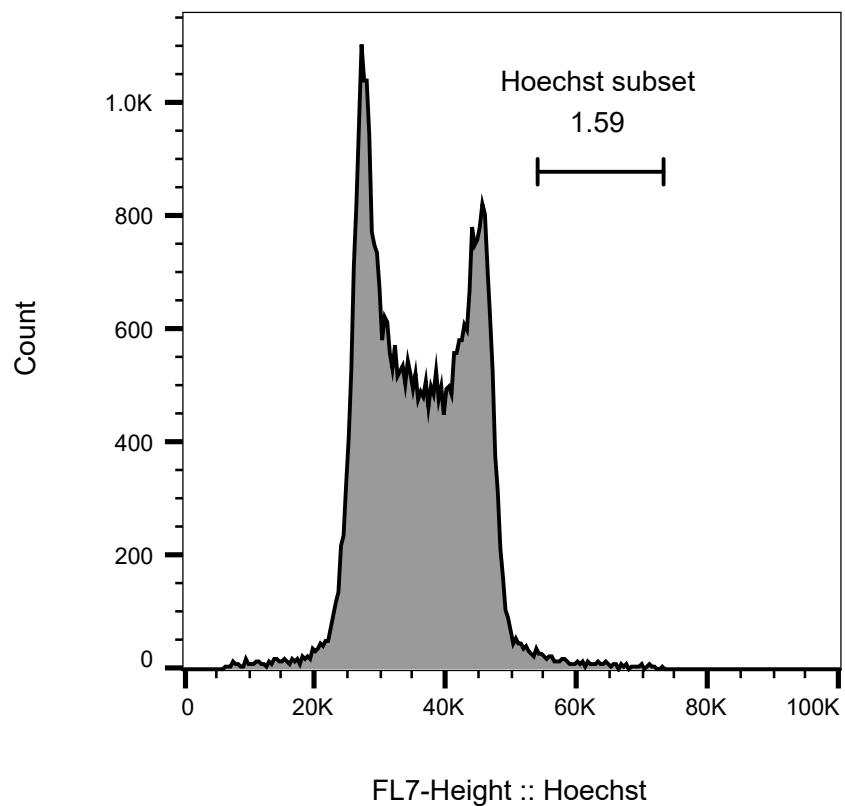

WT-D18

Supplement: Supplementary file 17 — Source data Fig. 3 [file 44320_2026_188_MOESM17_ESM.zip › Figure 3/3F/FASC WT D18.pdf]

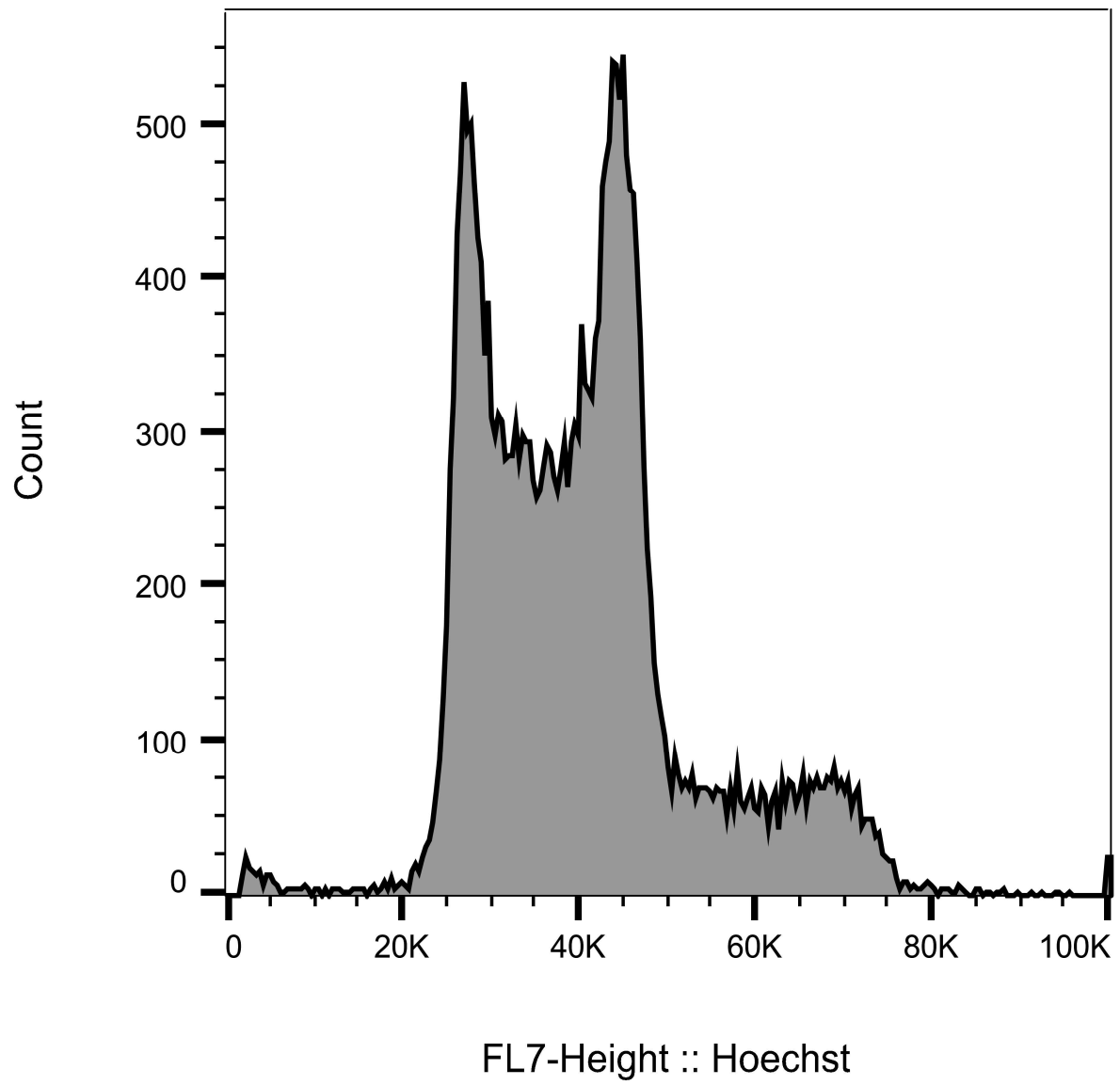

Supplement: Supplementary file 18 — Source data Fig. 4 [file 44320_2026_188_MOESM18_ESM.zip › Figure 4/4A/FACS Chr2+1 ESCs.pdf]

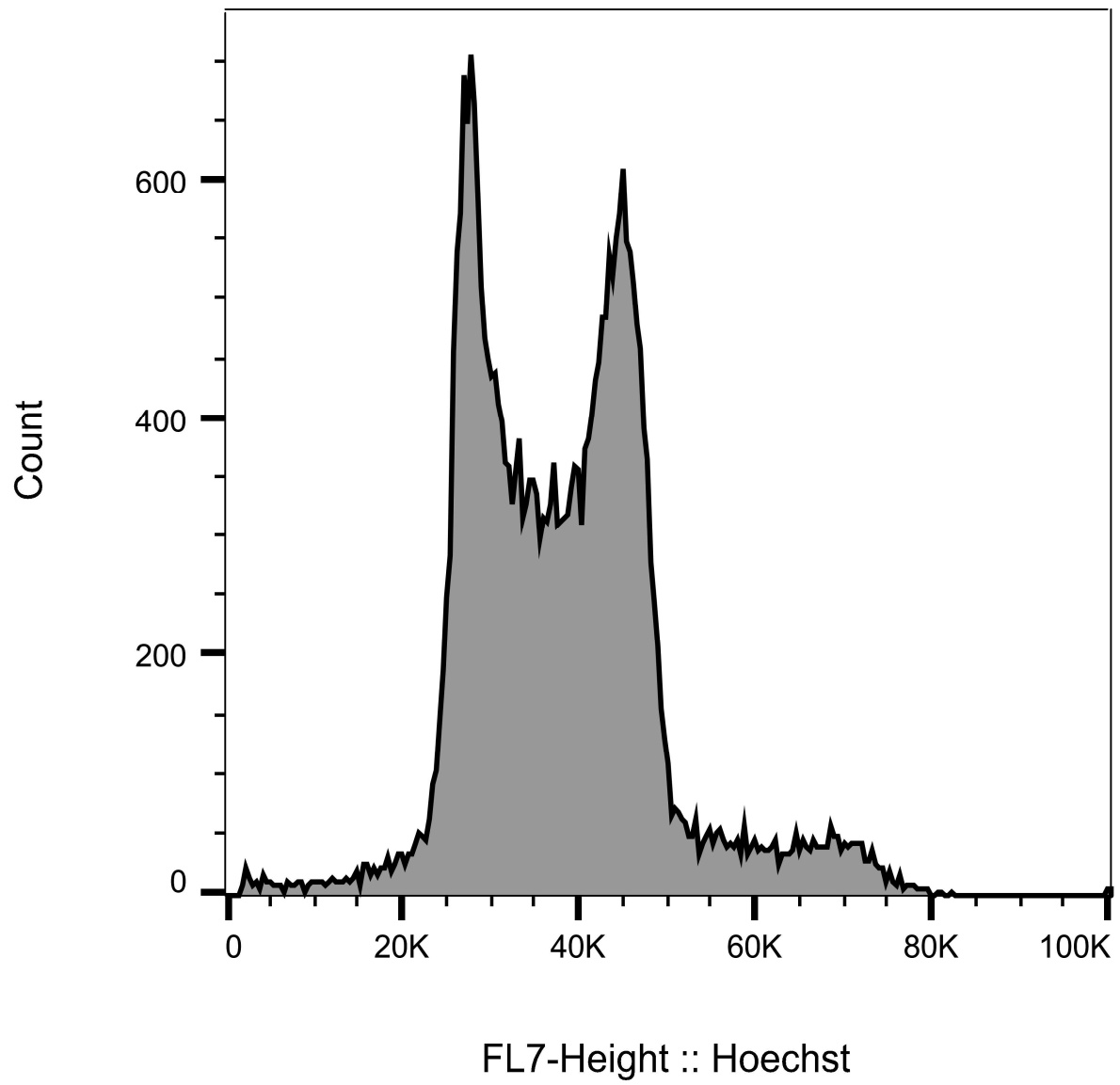

Supplement: Supplementary file 18 — Source data Fig. 4 [file 44320_2026_188_MOESM18_ESM.zip › Figure 4/4A/FACS Chr2+3 ESCs.pdf]

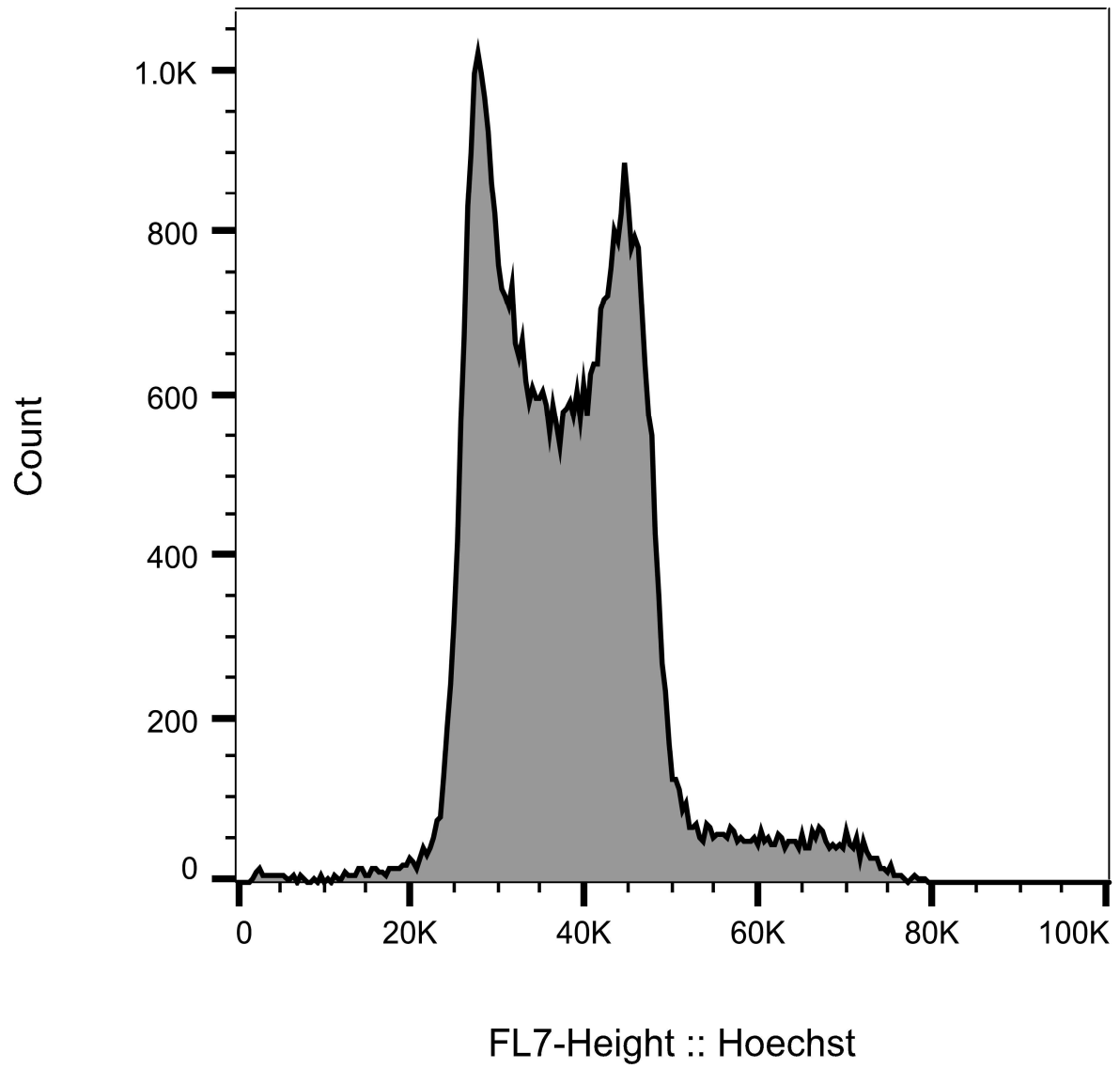

Supplement: Supplementary file 18 — Source data Fig. 4 [file 44320_2026_188_MOESM18_ESM.zip › Figure 4/4A/FACS Chr4+5 ESCs.pdf]

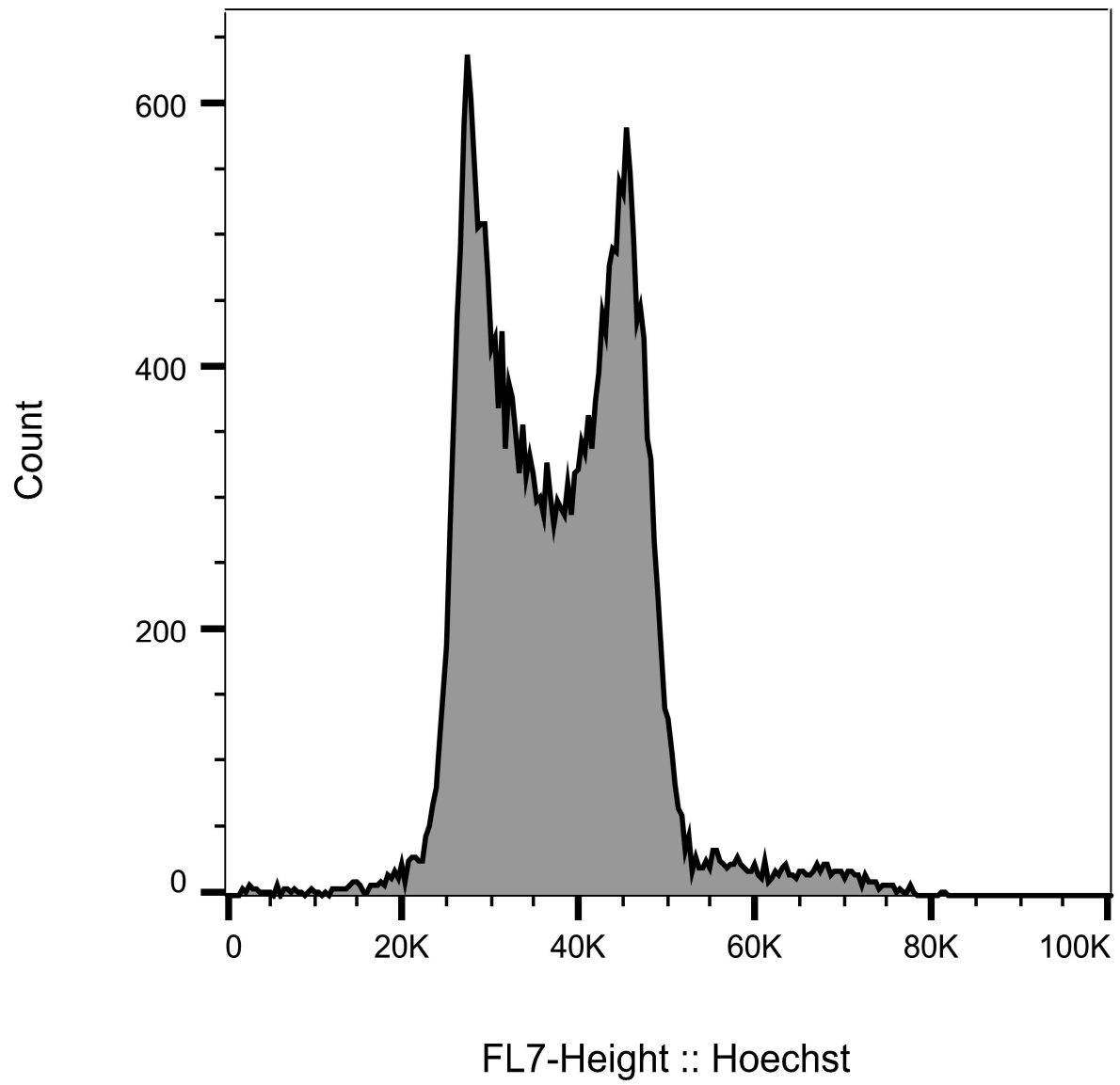

Supplement: Supplementary file 18 — Source data Fig. 4 [file 44320_2026_188_MOESM18_ESM.zip › Figure 4/4A/FACS WT ESCs.pdf]

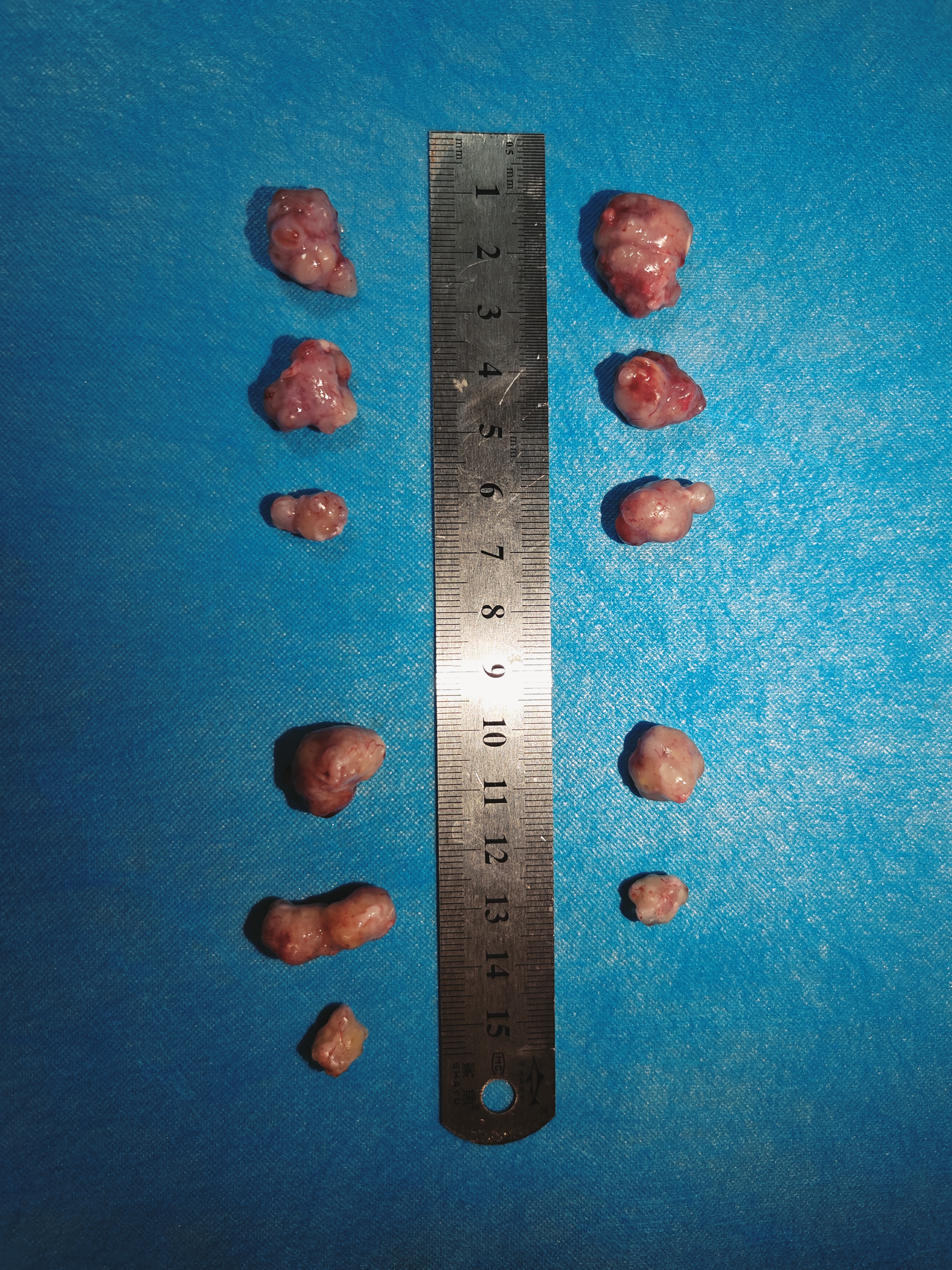

Supplement: Supplementary file 18 — Source data Fig. 4 [file 44320_2026_188_MOESM18_ESM.zip › Figure 4/4A/Teratoma all cell lines.jpg]

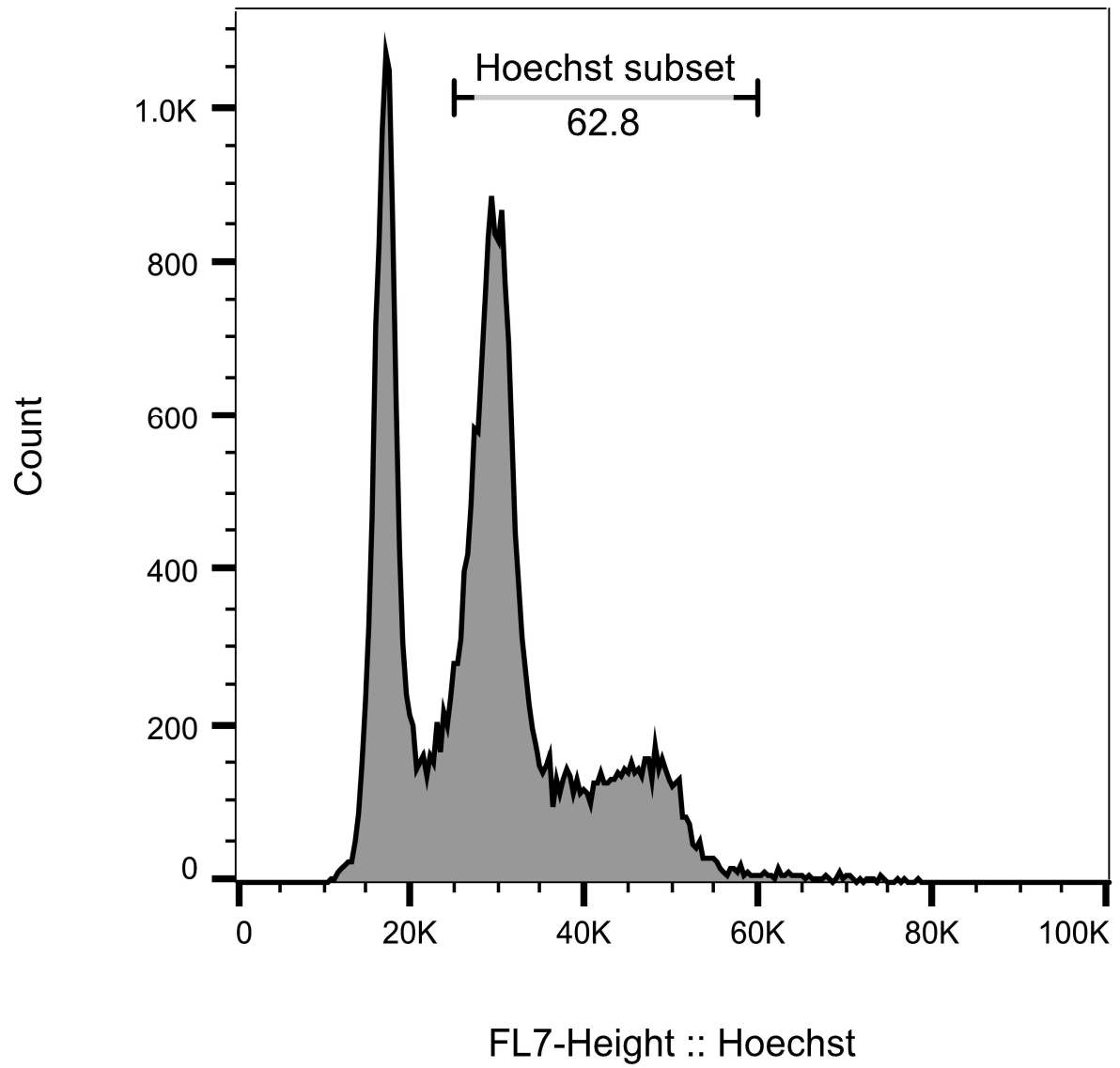

Supplement: Supplementary file 18 — Source data Fig. 4 [file 44320_2026_188_MOESM18_ESM.zip › Figure 4/4C/FACS Chr2+1 teratoma.pdf]

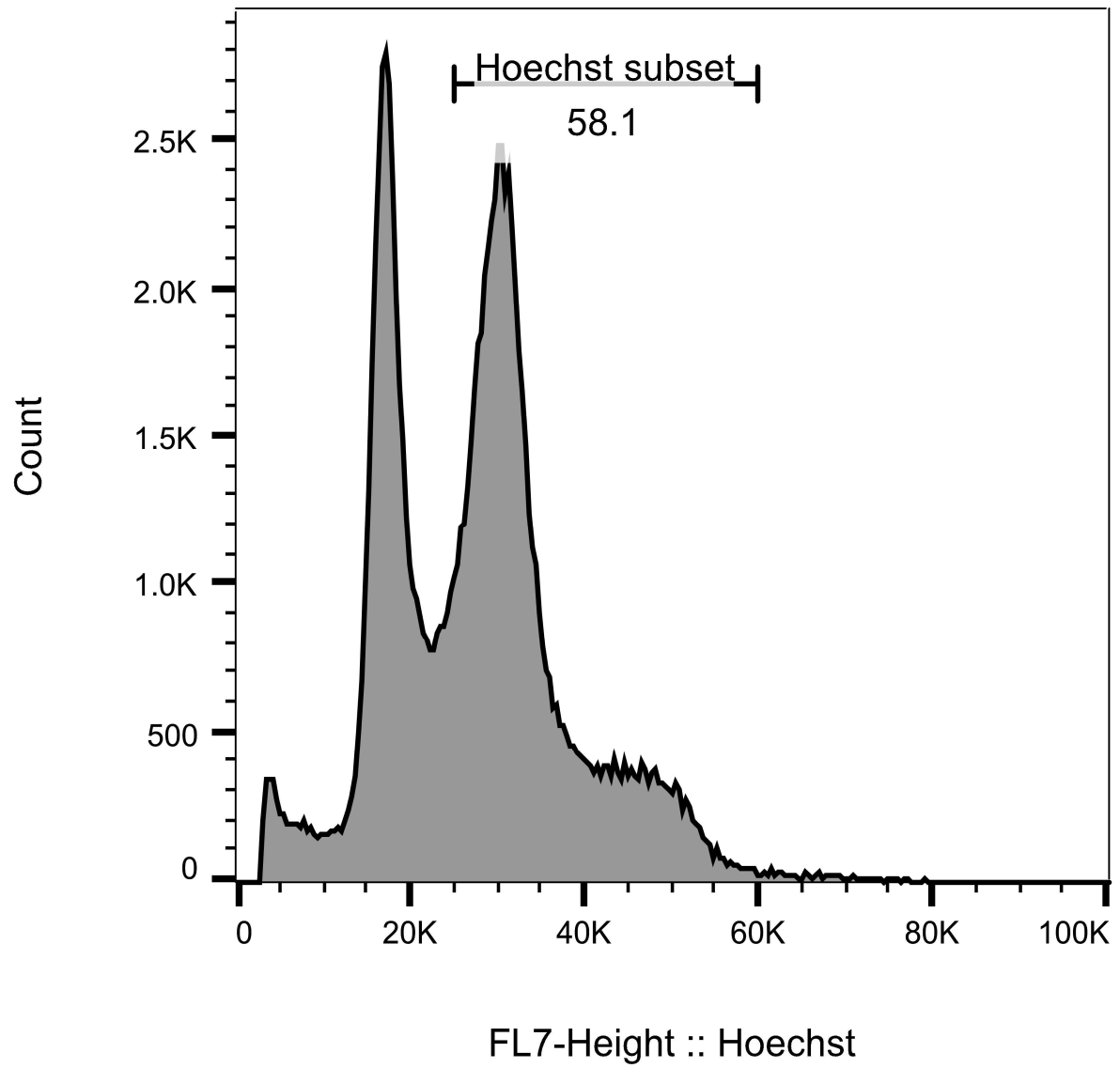

Supplement: Supplementary file 18 — Source data Fig. 4 [file 44320_2026_188_MOESM18_ESM.zip › Figure 4/4C/FACS Chr2+3 teratoma.pdf]

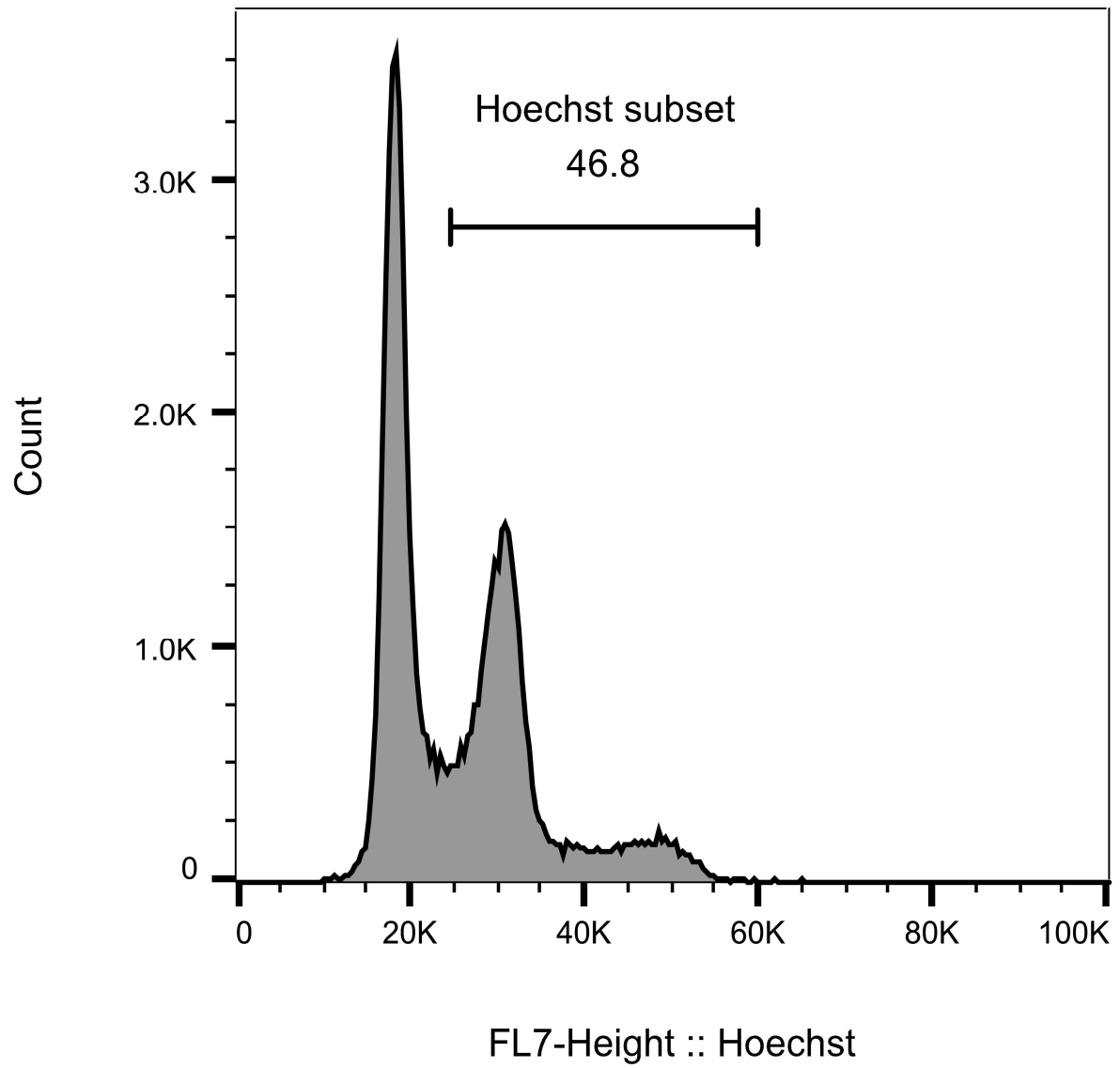

Supplement: Supplementary file 18 — Source data Fig. 4 [file 44320_2026_188_MOESM18_ESM.zip › Figure 4/4C/FACS Chr4+5 teratoma.pdf]

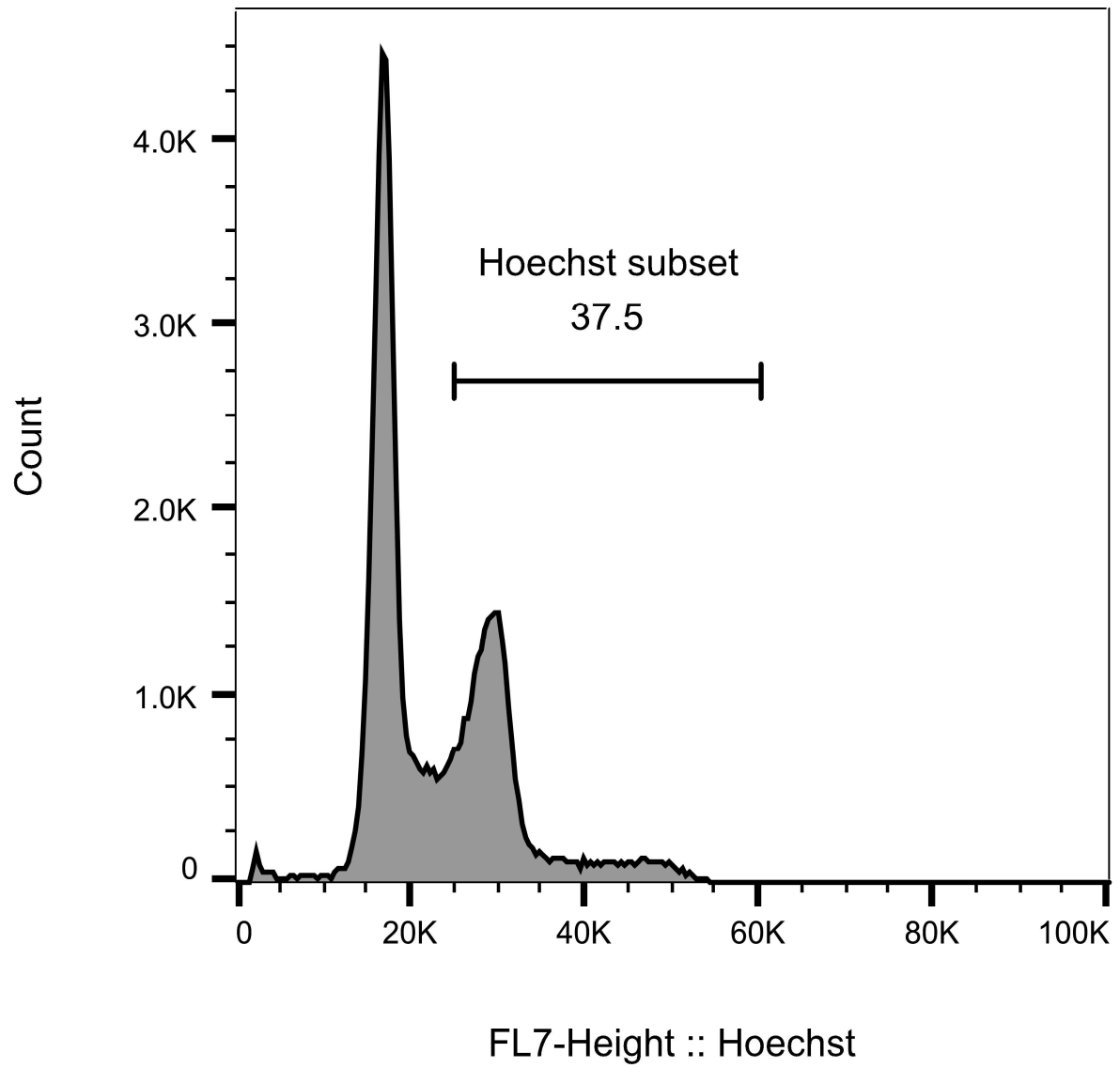

Supplement: Supplementary file 18 — Source data Fig. 4 [file 44320_2026_188_MOESM18_ESM.zip › Figure 4/4C/FACS WT teratoma.pdf]

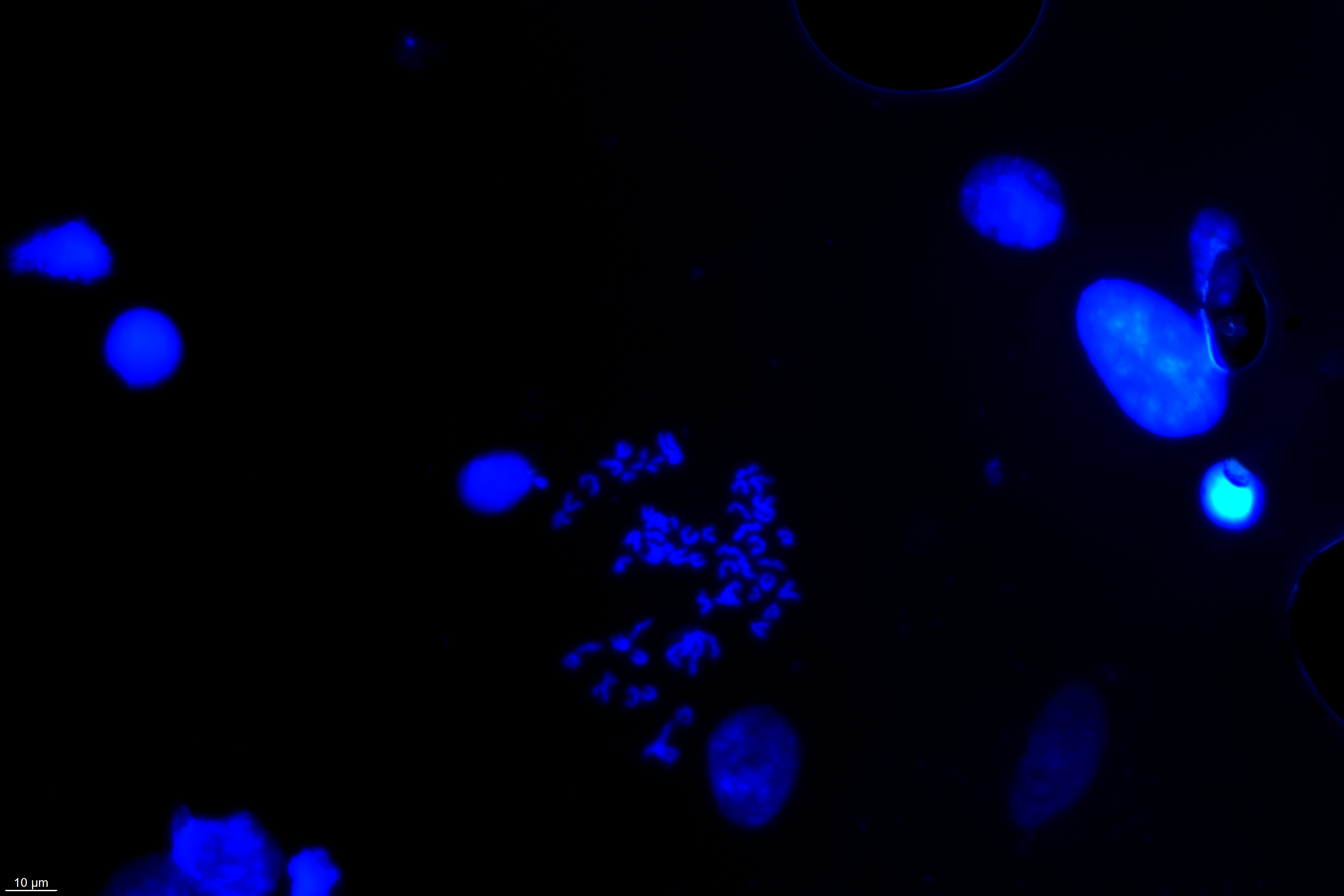

Supplement: Supplementary file 18 — Source data Fig. 4 [file 44320_2026_188_MOESM18_ESM.zip › Figure 4/4D/Chromosome spread Chr2+1.tif]

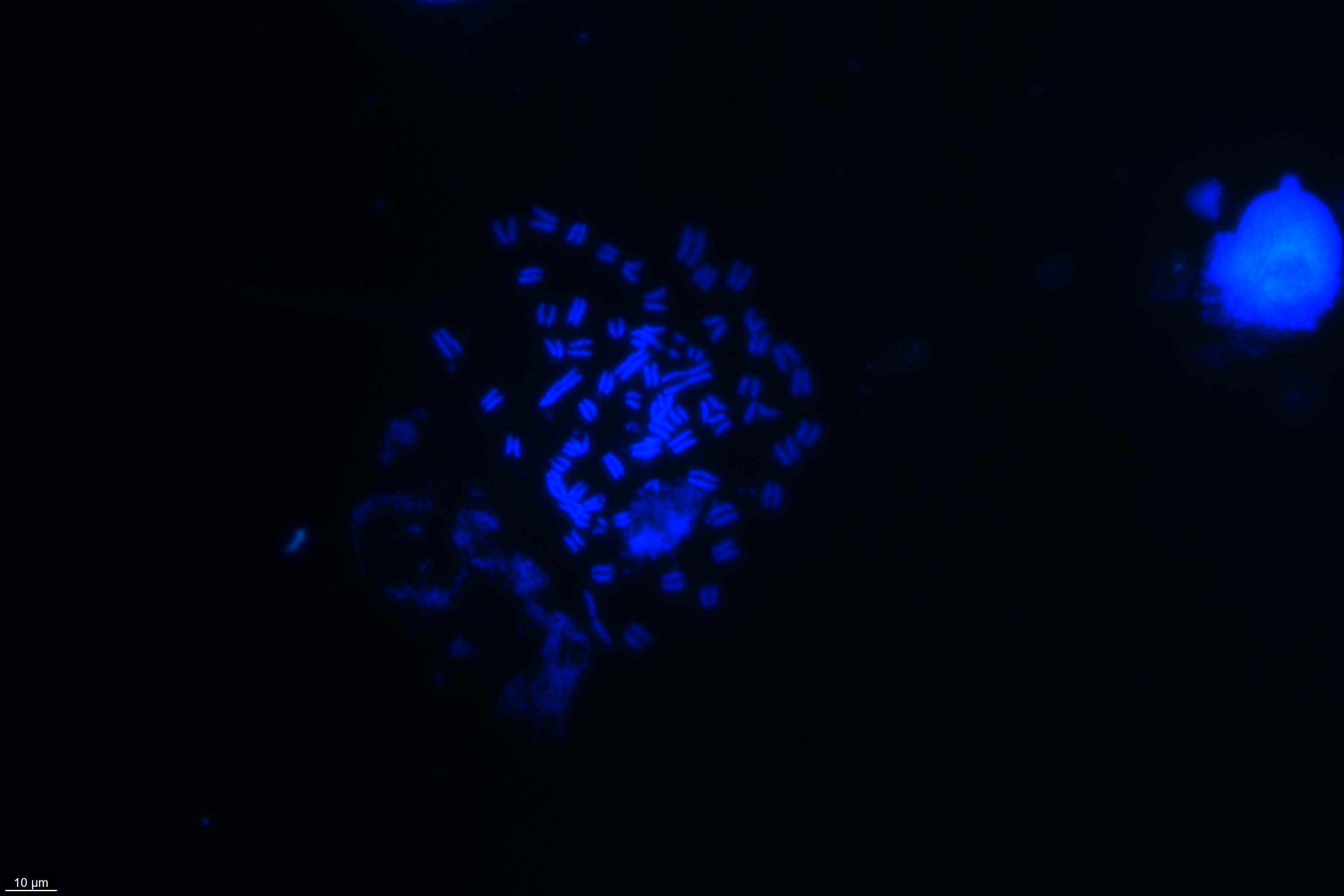

Supplement: Supplementary file 18 — Source data Fig. 4 [file 44320_2026_188_MOESM18_ESM.zip › Figure 4/4D/Chromosome spread Chr2+3.tif]

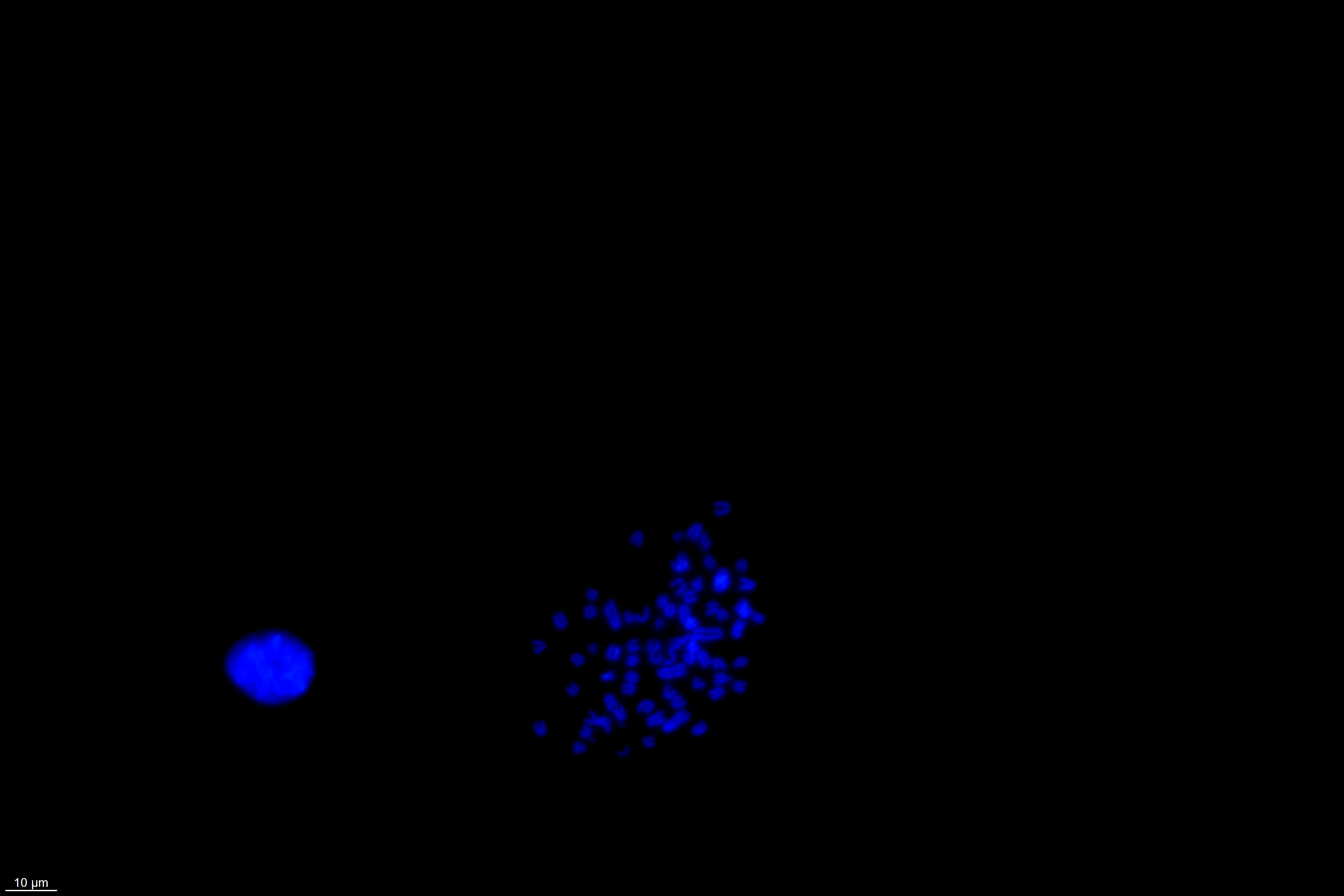

Supplement: Supplementary file 18 — Source data Fig. 4 [file 44320_2026_188_MOESM18_ESM.zip › Figure 4/4D/Chromosome spread Chr4+5.tif]

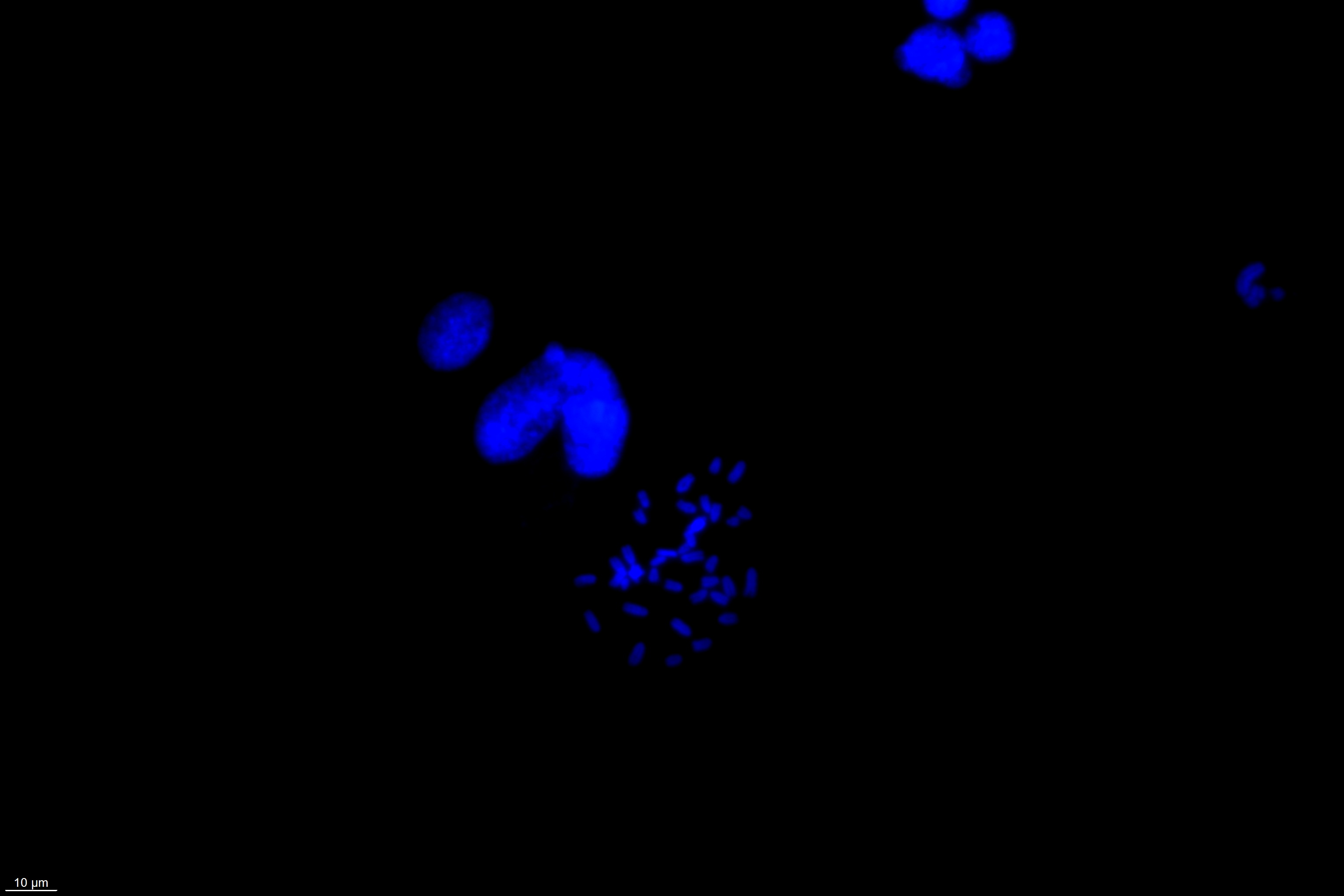

Supplement: Supplementary file 18 — Source data Fig. 4 [file 44320_2026_188_MOESM18_ESM.zip › Figure 4/4D/Chromosome spread WT.tif]

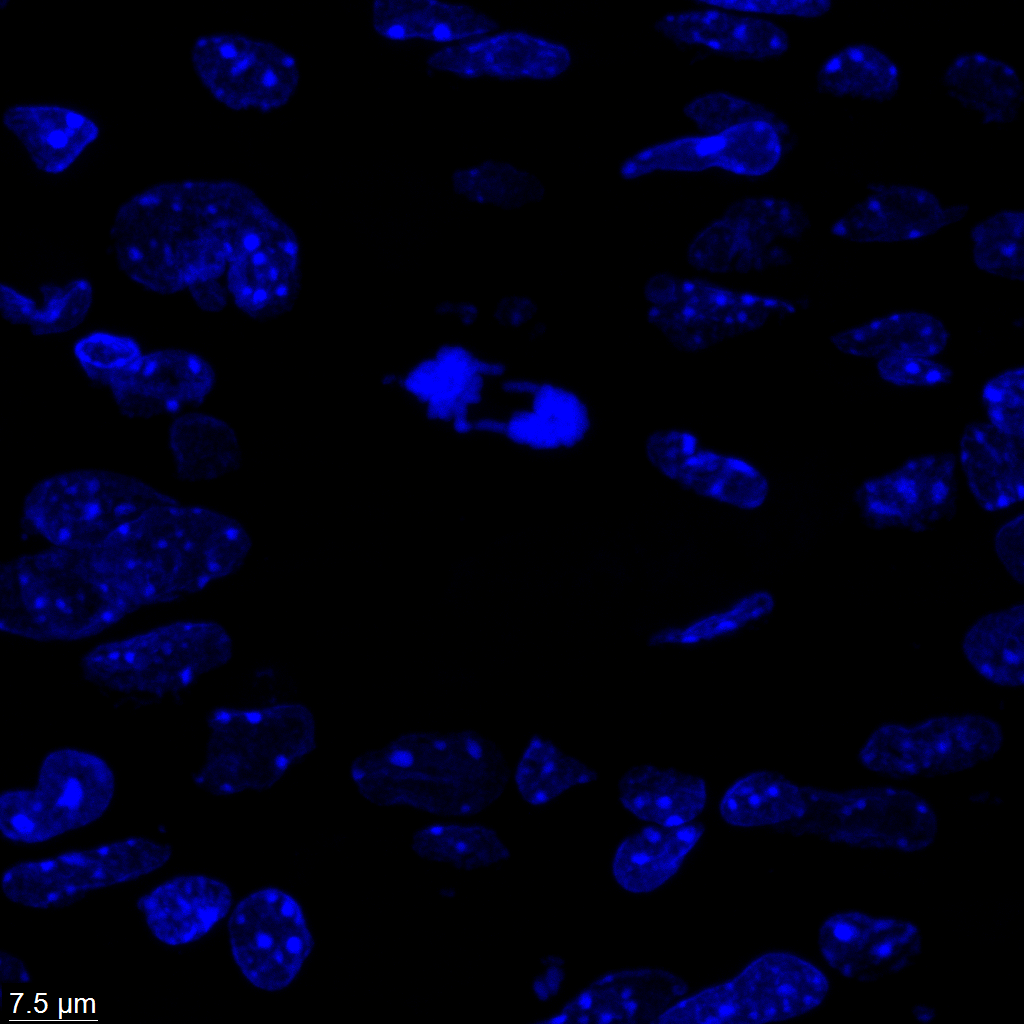

Supplement: Supplementary file 18 — Source data Fig. 4 [file 44320_2026_188_MOESM18_ESM.zip › Figure 4/4F/Confocal Chr2+1.tif]

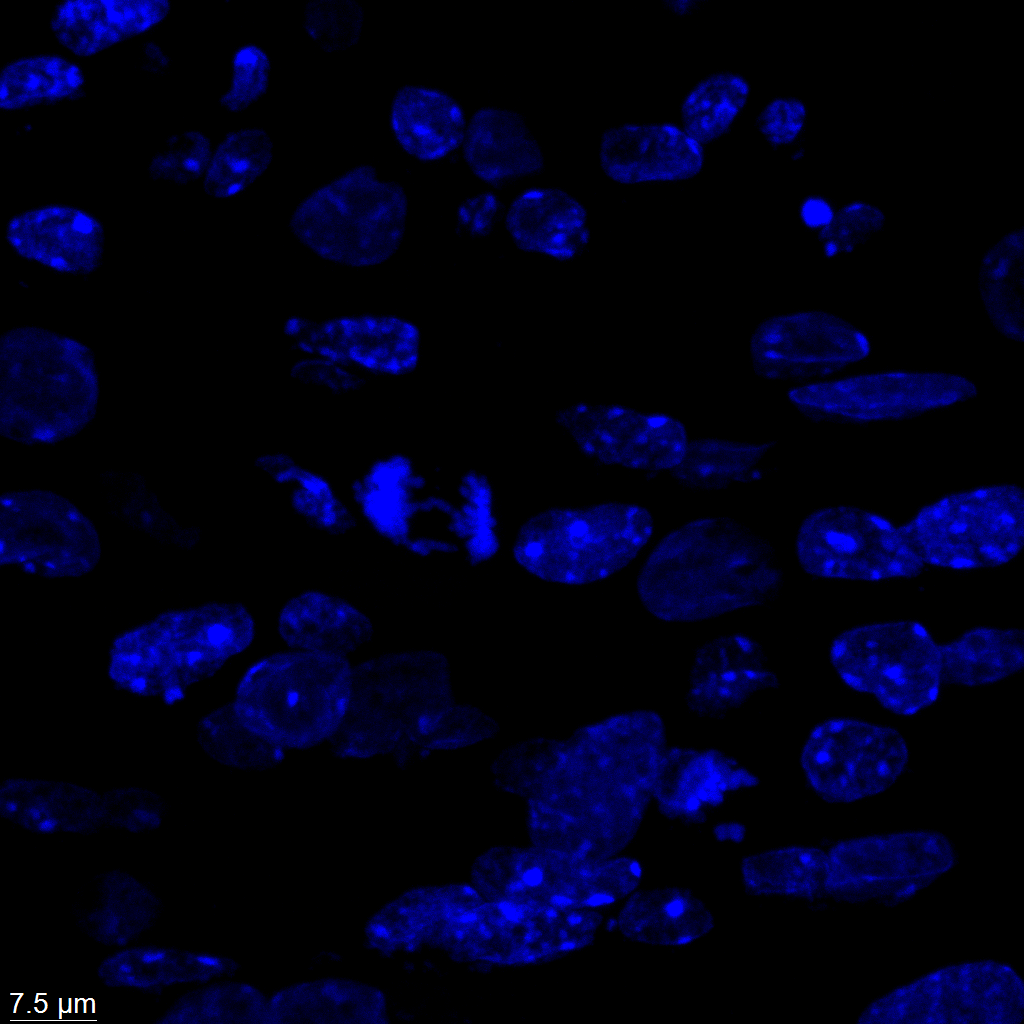

Supplement: Supplementary file 18 — Source data Fig. 4 [file 44320_2026_188_MOESM18_ESM.zip › Figure 4/4F/Confocal Chr2+3.tif]

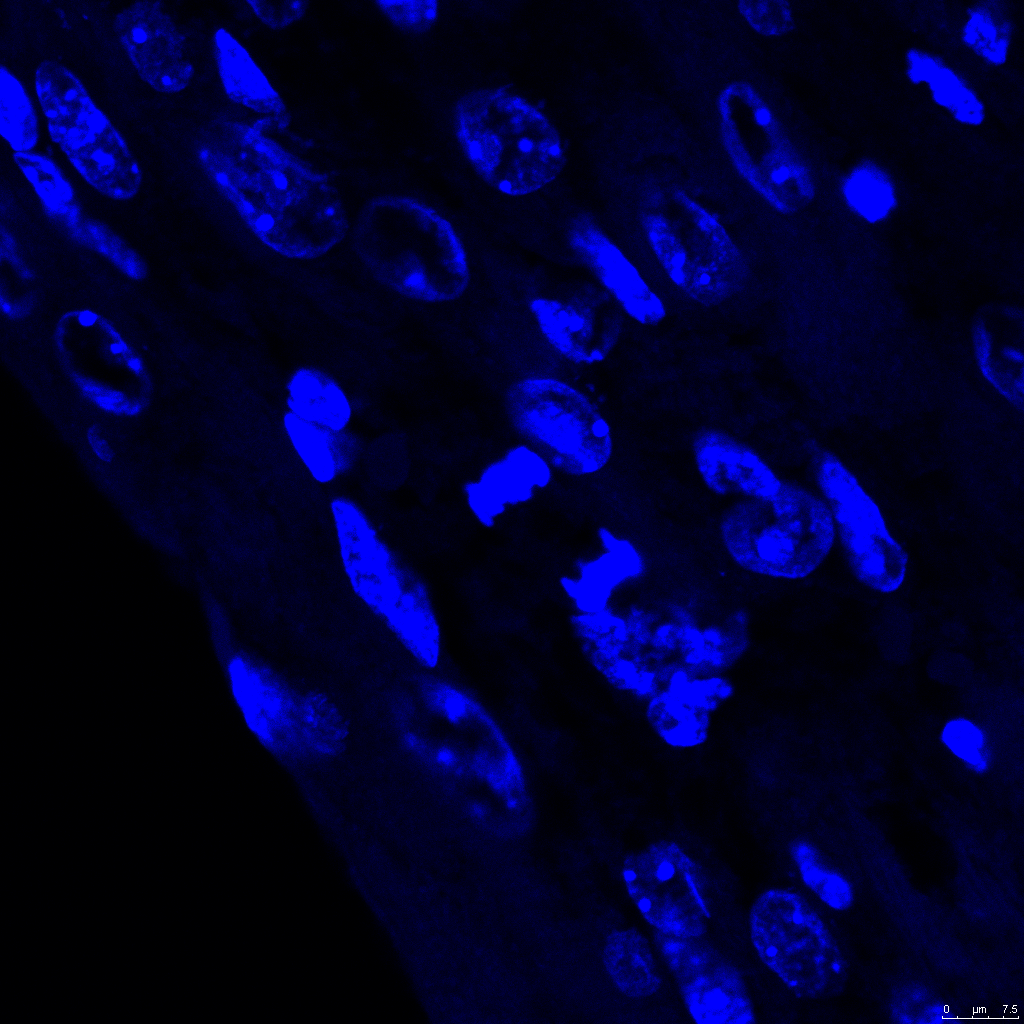

Supplement: Supplementary file 18 — Source data Fig. 4 [file 44320_2026_188_MOESM18_ESM.zip › Figure 4/4F/Confocal Chr4+5.tif]

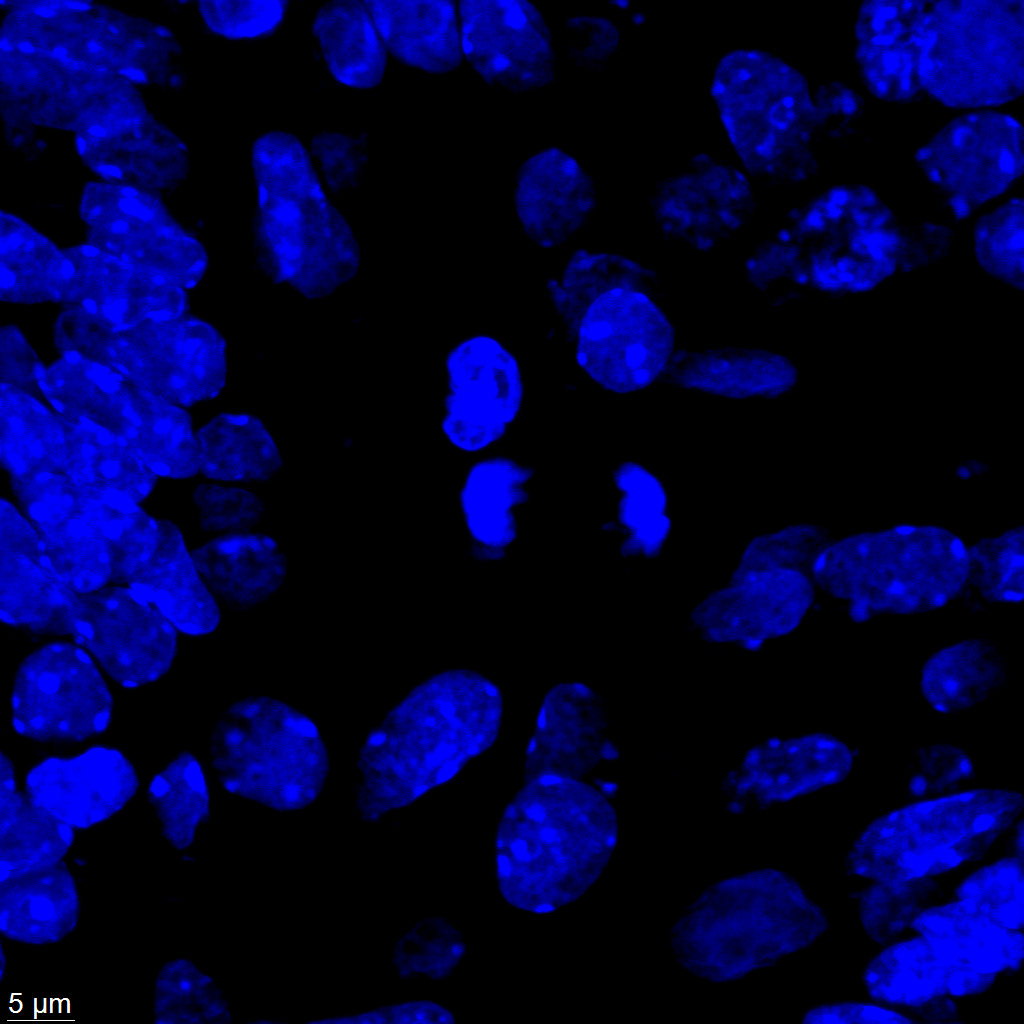

Supplement: Supplementary file 18 — Source data Fig. 4 [file 44320_2026_188_MOESM18_ESM.zip › Figure 4/4F/Confocal WT.tif]
