# Supplementary material for: Chromosome length is constrained by spindle scaling to ensure faithful mitosis in mammals
Source: Mol Syst Biol. 2026 Jan 15;22(4):480–96. doi: 10.1038/s44320-026-00188-8 (PMC13047055; doi:10.1038/s44320-026-00188-8)
Supplement: Supplementary file 19 — Expanded View Figures [file 44320_2026_188_MOESM19_ESM.pdf]

Expanded View Figures

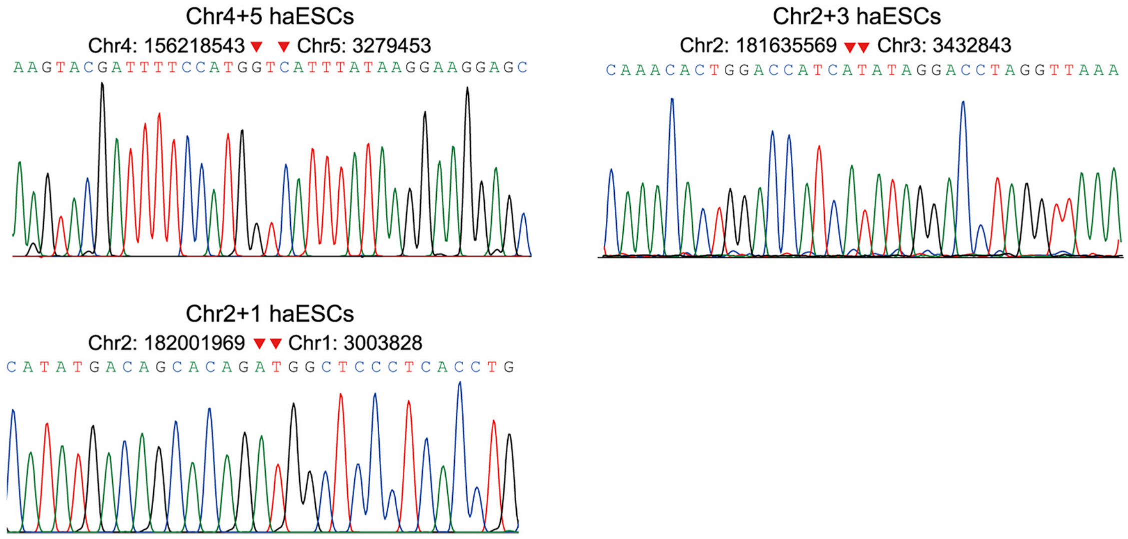

Figure EV1. Validation of chromosomal translocations in haploid embryonic stem cells.

Sanger sequencing verification of translocation breakpoint junctions. Red triangles denote fusion-site nucleotides at derivative chromosome termini.

 © The Author(s)

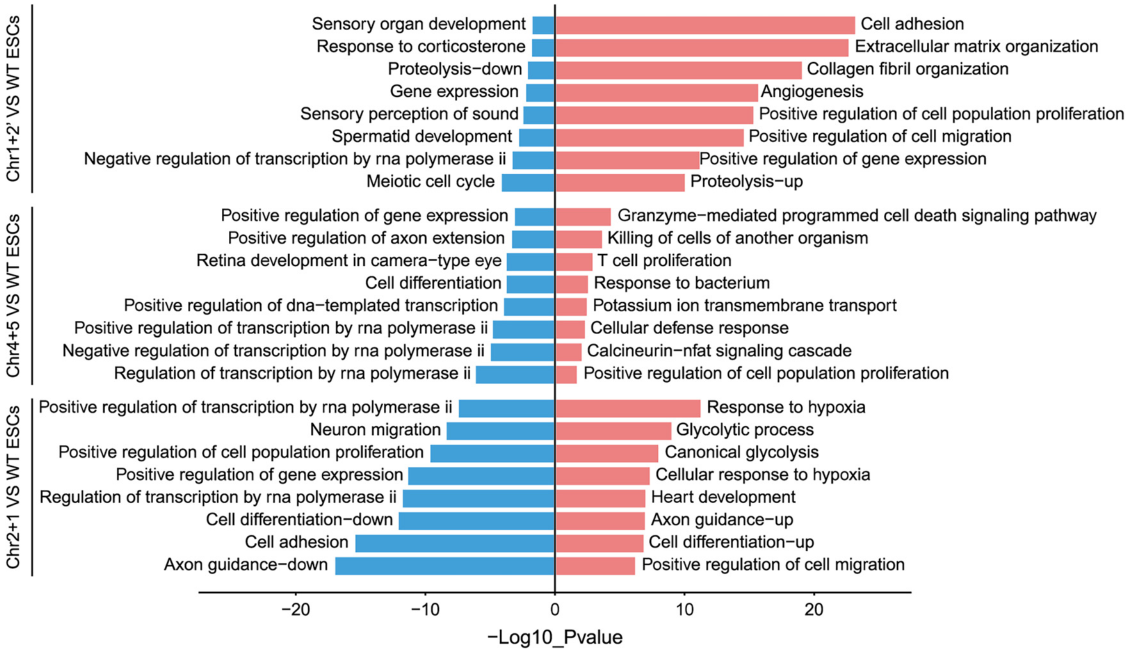

Figure EV2. Gene Ontology enrichment analysis of differentially expressed genes.

Gene Ontology enrichment analysis of differentially expressed genes in chromosome-translocated diESCs (Chr1 + 2′, Chr4 + 5, and Chr2 + 1) compared to WT diESCs. The *x* axis indicates the log_{10}-transformed FDR values, and the *y* axis lists the enriched pathway terms. Red bars represent upregulated pathways, and blue bars represent downregulated pathways. Enrichment analysis was calculated by using Fisher's exact test.

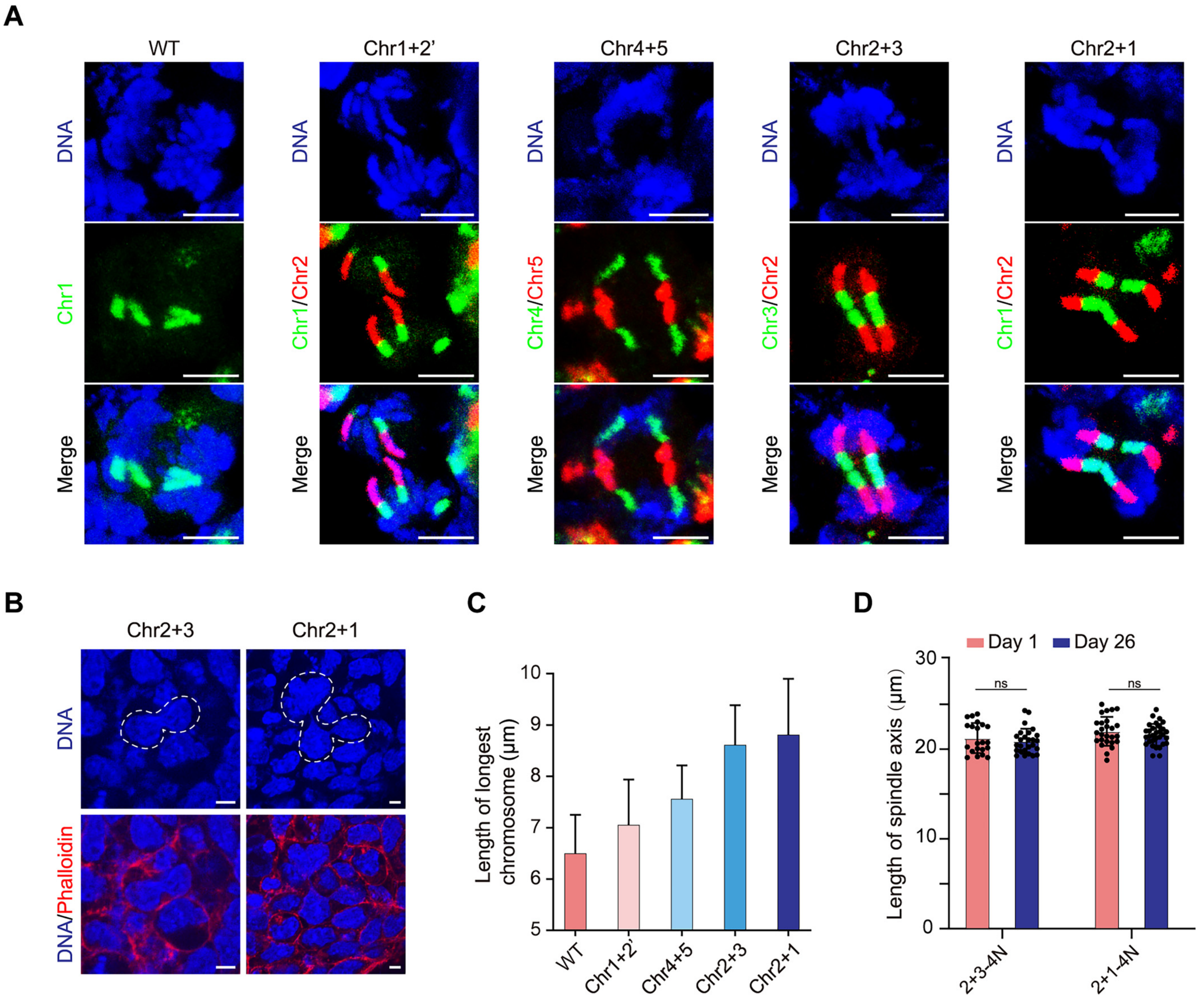

Figure EV3. Observations and measurements of translocated chromosomes and spindles at anaphase.

(**A**) FISH detection of lagging chromosomes at anaphase. Scale bar = 5 μm. (**B**) Immunofluorescence staining of Chr2 + 1 and Chr2 + 3 diESCs. Phalloidin staining indicates the spread of cytoskeleton, and white dotted circles label cells with binucleate and trinucleate. Scale bar = 5 μm. (**C**) Measurements of the longest chromosomes observed in live-cell imaging at the end of anaphase. WT: $n = 108$; Chr1 + 2′: $n = 44$; Chr4 + 5: $n = 45$; Chr2 + 3: $n = 58$; Chr2 + 1: $n = 76$. Data are presented as mean ± SD. (**D**) Comparison of spindle axis in tetraploid cells on day 1 and day 26. Day 1 Chr2 + 3-4 N: $n = 21$; Day 26 Chr2 + 3-4 N: $n = 26$; Day1 Chr2 + 1-4 N: $n = 26$; Day 26 Chr2 + 1-4 N: $n = 31$. Each data point represents one spindle axis. Statistical analysis was performed using unpaired *t* test. Data are presented as mean ± SD, with ns *P* value of 0.6598 and 0.3701 rightward correspondingly.

 © The Author(s)

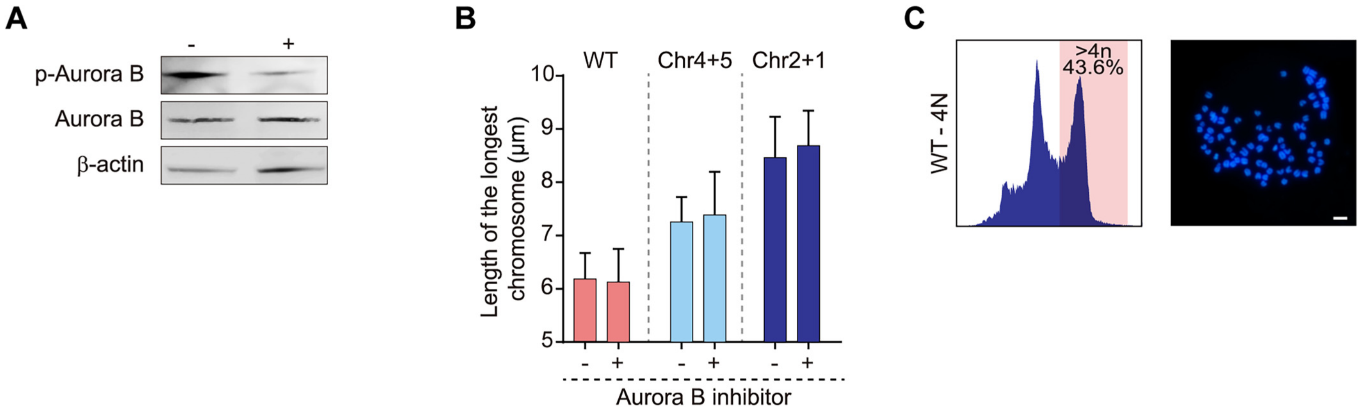

Figure EV4. Chromosome length and ploidy in WT diESCs following Aurora B inhibition.

(**A**) Immunoblot analysis of Aurora B autophosphorylation in WT diESCs with and without Aurora B inhibitor. β-actin serves as loading control. (**B**) Length comparison of the longest chromosomes in WT, Chr4 + 5 and Chr2 + 1 diESCs with and without Aurora B inhibitor. WT + Vehicle: $n = 59$; WT + Aurora B inhibitor: $n = 58$; Chr4 + 5 + Vehicle: $n = 57$; Chr4 + 5 + Aurora B inhibitor: $n = 59$; Chr2 + 1 + Vehicle: $n = 59$; Chr2 + 1 + Aurora B inhibitor: $n = 27$. Data are presented as mean ± SD. (**C**) FACS and karyotyping analysis of tetraploid WT ESCs. Tetraploid cells were isolated from WT diESCs following 18 days of Aurora B inhibition. The pink rectangle highlights the population with a DNA content >4n. Scale bar = 5 μm.
